# Our business, not the robot’s: family conversations about privacy with social robots in the home

**DOI:** 10.3389/frobt.2024.1331347

**Published:** 2024-03-21

**Authors:** Leigh Levinson, Jessica McKinney, Christena Nippert-Eng, Randy Gomez, Selma Šabanović

**Affiliations:** ^1^ Department of Informatics, Indiana University Bloomington, Bloomington, IN, United States; ^2^ WonderLab Museum Bloomington, Bloomington, IN, United States; ^3^ Honda Research Institute Japan, Wako, Japan

**Keywords:** privacy, family-centered design, co-learning, boundary management, contextual integrity, child-centered design

## Abstract

The targeted use of social robots for the family demands a better understanding of multiple stakeholders’ privacy concerns, including those of parents and children. Through a co-learning workshop which introduced families to the functions and hypothetical use of social robots in the home, we present preliminary evidence from 6 families that exhibits how parents and children have different comfort levels with robots collecting and sharing information across different use contexts. Conversations and booklet answers reveal that parents adopted their child’s decision in scenarios where they expect children to have more agency, such as in cases of homework completion or cleaning up toys, and when children proposed what their parents found to be acceptable reasoning for their decisions. Families expressed relief when they shared the same reasoning when coming to conclusive decisions, signifying an agreement of boundary management between the robot and the family. In cases where parents and children did not agree, they rejected a binary, either-or decision and opted for a third type of response, reflecting skepticism, uncertainty and/or compromise. Our work highlights the benefits of involving parents and children in child- and family-centered research, including parental abilities to provide cognitive scaffolding and personalize hypothetical scenarios for their children.

## 1 Introduction

Introducing intelligent and interactive technologies such as social robots into the personal context of the home poses unique challenges to the boundary between public and private life. The dynamic, multi-user context of the home means that designers must consider different use cases and perspectives of diverse family members, including young users from infancy into adolescence. A review of longitudinal research with robots in spaces like the home recommends designing robots that strategically recall previous activities and self-disclosure, may understand a user’s emotions and react in contextually appropriate ways, and use information about users to personalize interactions (Leite et al., 2013). Such demands make robots inseparable from the privacy concerns that come with data collection in the child’s home. While privacy with social robots in the home is understudied, research with families and robots in the home identifies that family member privacy is frequently brought up as a future concern to be addressed (Cagiltay et al., 2020; Garg and Sengupta, 2020). Children between the ages of 6–12 are particularly vulnerable, as they may not fully grasp the data privacy concerns, or access that third parties like parents have to information, with artificially intelligent agents (Andries and Robertson, 2023). There are also ethical concerns about social robots that take on caregiver roles and their potentially harmful consequences on psychological and emotional wellbeing of children (Sharkey and Sharkey, 2010).

Further risk is imposed with embodied social robots which elicit more sympathy and trust (Luger and Sellen, 2016) and self disclosure (Calo et al., 2010) than with other smart devices. In fact, the functional and emotional benefits that social robots can provide compounded with their social influence and autonomy will also impact uses and roles for robots, heightening concerns surrounding physical, psychological, and social privacy (Lutz et al., 2019; Lutz and Tamó-Larrieux, 2020). A family member’s different relationship and perception of the robot may further exacerbate differences in concerns. Often, privacy research which considers privacy concerns of family members considers their concerns separately (Levinson et al., 2023). However, their mutual influence and ownership over information in the home demands a more nuanced understanding of familial privacy concerns. Neglecting to understand how different stakeholders in a family negotiate the use of social robots as beneficial while simultaneously endangering their privacy constitutes a missed opportunity for identifying the most desirable design features and use contexts for future robots. The shift to studying child-robot interactions from a family lens can better consider the family stakeholders’ involvement, expectations and concerns when introducing a robot into a home, as well as account for how the robot may affect family dynamics (Cagiltay et al., 2023).

In this study, we present conversations between parents and children surrounding hypothetical scenarios with a robot in a family home. We find that parents and children readily engage with privacy related questions as conversational partners and will adopt the other’s decision according to their level of asserted agency and ownership of the information relevant in that scenario. Families were also relieved when they shared the same reasoning, signaling a mutually identified boundary for the role and responsibility of the robot. In cases of uncertainty or disagreement, families also created their own answer category, reflecting the nuance required for privacy boundary management with robots in the home. Together, we argue that studies that incorporate family negotiations better define the role and responsibilities of family-centered robots.

## 2 Background

### 2.1 Social robot research with families

As more robots are being designed and introduced in the home, researchers are finding ways to understand how they fit into family life. Often, family research with robots is conducted through cohabitation, where a robot stays in the home for a longer period. Such experiences allow researchers to learn about family member’s expectations (Fernaeus et al., 2010), habits (Forlizzi, 2007), and perceptions (Levinson et al., 2023) of robots in the home. However, researchers do not need to rely on established platforms to be in the home. Other methods which do not necessarily require longitudinal data or a prototype in the home include workshops or participatory design sessions.

Co-learning is a method in which caregivers are involved in the education of robots and coding with their children, promoting better understanding and confidence in STEM (Chung and Santos, 2018; Govind et al., 2020). Some co-learning experiences with robots in the home also include learning modules about data security and privacy-sensitive robotics (Ahtinen et al., 2023). We utilize co-learning in a museum setting to encourage family participation in discussing privacy with roots in the home.

Part of learning together can involve explicit co-designing with researchers through participatory design. Researchers can elevate participant imagination by combining interview and discussions with fiction based play, including dollhouses and figurines which families used to demonstrate how they could add a robot into their family life (Cagiltay et al., 2020). These workshops found that family members differ in choice roles and interactions with the robot which range from companionship to assistance (Cagiltay et al., 2020).

Within these methods, researchers often use prototypes or design artifacts that trigger discussions to engage with families (Mogensen and Trigg, 1992). For example, Christensen et al. (2019) use a blank, shared calendar as a starting point for families reflect on their technology use. They also use social drawing where caregivers and children draw together as a means of reflection (Christensen et al., 2019). In our workshop, we take inspiration from these co-learning and design methods in leading a workshop for families featuring a novel stimuli booklet, drawing activities, embodied play, and family discussion to gain a better understanding of the ways parents and children talk about privacy when it comes to robots in the home.

### 2.2 Family communication privacy management

Boundary management and the implicit lines around privacy, power, and responsibility are fundamental aspects of Bowen’s family systems theory (Minuchin, 1985; Bowen, 1993; Haefner, 2014) which has been adopted across many disciplines. Communication privacy management, in particular, posits that individuals view themselves as owners of their own information and that sharing information with others grants them co-ownership to that information (Petronio, 2017). In this “procedure”, there are certain rules that co-owners will follow based on mutually agreed expectations, and any sharing beyond this is considered a violation of privacy. This is particularly relevant in the case of families who are constantly needing to establish these boundaries as co-owners of information. Boundary turbulence further occurs when co-owners do not have the same understanding of privacy rules (Petronio, 2002). In the family context, these boundaries and the turbulence around them are highly related to power dynamics and emotions (Petronio, 2010) and changing contexts render boundaries dynamic, often requiring rules to be negotiated and boundaries to be re-drawn (Petronio and Child, 2020).

In our case, this framework applies not only to the interpersonal communication and privacy strategies used between family members, but also with the robot and third parties. Boundary management also becomes complicated in information sharing with social robots who may not be able to navigate social norms or expectations surrounding privacy. This is further elevated if users are unaware when and if the robot is collecting and sharing information. Increased social cues and anthropomorphism may increase trust (Darling, 2015; De Visser et al., 2016), and particularly for child users, could establish false senses of security (Zhao et al., 2019). Our workshop opens up opportunities for families to begin navigating their boundaries with robots in the home and recognizes the ways families will need to decide when and if robots can be involved in co-ownership of their information.

### 2.3 Technology can shift family dynamics

Rather than viewing power as an individual attribute, a dynamic understanding recognizes the reciprocal and situation-specific nature of power in parent-child relationships (Beckman-Brindley and Tavormina, 1978; Maccoby, 1984). Through viewing children as actors and agents in their relationships, parents become more receptive to their child’s influence over time (Prout, 2002; Kuczynski et al., 2016). We can further understand social robots in the home as becoming part of a family system (Cagiltay et al., 2023), participating as an agent in subsystems and influencing power dynamics in the family.

The primary focus of family socialization and discipline literature focuses on early development (Fritchley-Griffith, 1996). However, less is known about privacy dynamics between parents and children in middle childhood. Inherent to the name ‘middle childhood’, the period of time between 6 and 12 years old, are a transitional period where children are no longer toddlers but they are not yet adolescents, adopting more agency and power in the family. Technology poses further challenges to typical power dynamics because technology can give children more knowledge, skills and resources to leverage in the home (Sun and McMillan, 2018). Whether or not children are “digital natives” who are more tech savvy than adults because they have been immersed in a technological era since birth (Marc, 2001; Bennett, 2012), children’s experience with technology has implications on family interactions and dynamics. Though robots demand different forms of interaction than digital media, they too give children agency in the home in ways that will be different than their parents, implicating different privacy concerns and considerations.

Alternatively, technology also gives parents additional ways to monitor their children’s behavior, such as asserting more control over children’s schedules or screen-time (Geeng and Roesner, 2019). There are also power imbalances with smart home devices between primary and secondary users (Zhang et al., 2017). Though parents are less likely to be a primary user of a child-centered social robot, they will be most likely the purchaser and owner of the technology, implicitly asserting their power on the adoption of the technology into the home. Additionally in the case of a family-centered social robot, the identity of the primary user may also shift contextually, begging the question of different power dynamics across contexts. With these contextual shifts in power and family dynamics, we probe into how children in middle childhood and parents jointly discuss the use of social robots in the home and their largest concerns surrounding privacy.

## 3 Methods

### 3.1 Participants

We hosted a robotics workshop twice at a local museum and solicited participation from caregivers and children interested in learning about robots in the home. Advertisements for the workshops were placed on the museum’s website and researchers also spread the word locally. On the day of the workshop, families who were already in attendance at the museum were invited to join and participate. Guardians filled out informed consent documents and children 7 years old and older were provided an assent document with simplified details about the study and for any photos displayed in this paper, children and parents signed a media release giving explicit consent to have photos published in an academic publication. The study was also approved by Indiana University’s Review Board #18601. A total of 8 families participated in the study (See Table 1).

**TABLE 1:** Participant Information, *Participants were excluded from analysis for child age, ^+^Participants excluded for non parent-child relationship.

Family	Caregiver	Children (age)
F1	Father	Son (7), *Son (5) *Daughter (5)
F2	Father	Daughter (11)
F3	Mother	Daughter (10)
*F4	*Mother	*Son (6)
F5	Mother	Daughter (7)
F6	Mother	Son (10)
^+^F7	^+^Aunt	^+^Niece (10)
F8	Mother	Son (7)

Though families were excited participants during the workshop, it was during the subsequent transcription process that we realized children younger than 7 were not old enough to engage meaningfully with our materials. Therefore, we excluded F4 whose child was 6 years old and the two 5 year old children in F1 who did not fully participate in the compromise activity. We also recognized that power dynamics for robots in the home were household specific, yet one of our participating families included an aunt and niece who did not live together and who approached the questions under a different framing. Therefore, we exclude F7, this niece and aunt pair, from the final analysis.

### 3.2 Museum as setting

As mentioned in the background, co-learning is one method to encourage a deeper understanding and confidence in technology for a household. Museums are an excellent space for cross-generational learning, as many exhibits provide new information for everyone in the household (Falk and Dierking, 2018). Through learning together, families build their relationships, confidence, and understanding in new information.

Museums also allow for a continued informal learning for children that supports what they learn day-to-day (Crowley et al., 2014). Children are able to connect what they already know to a new subject with the support of an adult. In areas of study that adults are more comfortable with or knowledgeable about, they are able to encourage their children’s learning by *scaffolding* what they have already experienced with this new subject. Cognitive scaffolding is said to be situationally dependent and is helpful for children’s executive function and intuition development (Mermelshtine, 2017). This scaffolding is highly related to parental socialization (Gauvain, 2005), including the ways that parents are involved in raising concerns children may not think about when they are faced with new technologies which offer enticing benefits and functionalities. Informal education is a large aspect of the museum setting, allowing this scaffolding to occur naturally and frequently. A review on interventions in museums identifies that these institutions significantly affect parent-child conversations in a positive way, particularly those that gave prompts to parents for increasing the specificity of their language (Kupiec et al., 2023). The workshop detailed below occurs in a local science museum which frames the parent-child co-learning about robots in the home.

### 3.3 Stimuli booklets

The main device used to stimulate discussion between children and their parents were booklets which outlined hypothetical scenarios of Haru the robot in the home across different contexts and asked users to indicate how comfortable they were with Haru partaking in different interactions. These booklets rooted our mixed method design (Ivankova and Creswell, 2009), which embedded quantitative booklet responses into the conversations between families and subsequent qualitative data collected.

**FIGURE 1:**
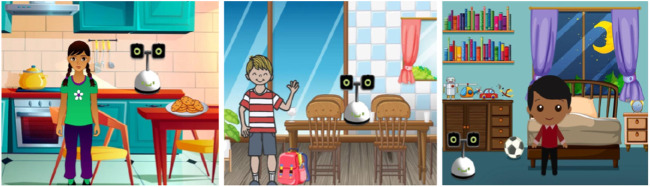
All 3 scenarios from the booklet from left to right: Ellie (she/her) and Haru in the Kitchen, Carter (he/him) and Haru doing Homework in the Dining Room, and Sam (they/them) and Haru in the Bedroom.

In choosing the questions featured in the booklet, we were intentional to think not only of realistic yet hypothetical uses for the robot, as has been done across social robotics literature (Enz et al., 2011), but also to consider how privacy varies across context, for example including more public spaces (like the kitchen and dining room) to a more private space (child’s bedroom). We also considered past literature which envisions robots domestic helpers in the kitchen (Guizzo, 2010), robots as companions for reading bedtime stories (Zhao and McEwen, 2022) and cleaning up toys (Fink et al., 2014), and home-robot assisted learning and tutoring (Han et al., 2005; Han et al., 2008)The theory of contextual integrity recognizes how comfort with information sharing shifts with respect to social norms as they differ across social contexts, actors/third parties, information type/content, and who has access to that information, including who co-owns the information (Nissenbaum, 2004). As such, three contexts were selected which varied in location, task, level of mediation of the robot, and third parties who were involved in information sharing. In order to stimulate discussion across different outcomes, the questions also varied in potential positive, negative, and neutral consequences for the characters in the story. We further varied the pronouns, skin colors, and perceived age of the children in scenarios, though none were explicitly introduced before reading the stories (See Figure 1). We also believed that added transparency about how the robot was carrying out the given use scenario would impact family member comfort levels with the robot and wanted them to be able to make the most informed decisions. As such, we asked participants to consider these scenarios both with and without added explanation on how the robot was functioning.

**FIGURE 2:**
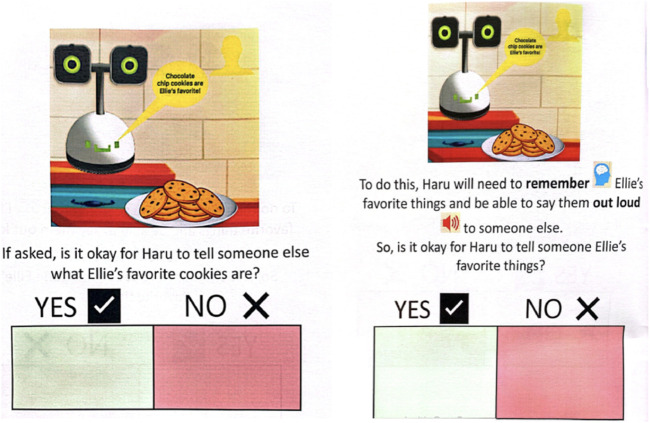
Example of blank booklet with first scenario question on the left, explanation question on the right.

First, the booklets proposed a scenario with the robot Haru (See Section 2) and asked participants to decide if the robot’s use was okay (“Yes”) or was not okay (“No”) in that context. Then, the booklet re-presented the scenario with more explanations of which sensors the robot used, such as those described in the introduction and again asked participants to decide if the function was okay or not okay in that scenario (See Figure 2).

Below, we summarize each booklet (Ex: Scenario 1 as **S1**) followed by the explanatory sensors and capabilities used in that scenario (Ex: camera, microphone):

• **Ellie and Haru in the Kitchen (6 scenarios)**: Ellie enters the kitchen where Haru always stays. When she enters, Haru turns on and says hi. She notices there is a plate of cookies on the table. There are nine left and they look REALLY good. Ellie says out loud, “Chocolate chip cookies are my favorite!!” **Kitchen S1)** Is it okay for Haru to remember that chocolate chip cookies are her favorite? (microphone and memory), **Kitchen S2)** If asked, is it okay for Haru to tell *someone* else what Ellie’s favorite cookies are? (memory and speech), **Kitchen S3)** Is it okay for Haru to see the kitchen and cookies? (camera and saving pictures), **Kitchen S4)** Is it okay for Haru to remind Ellie when she can eat a cookie? (know time and Ellie’s schedule), **Kitchen S5)** Is it okay for Haru to tell Ellie a cookie fact? (internet), and **Kitchen S6)** If Ellie’s *parents* ask, is it okay for Haru to tell them that Ellie ate an extra cookie? (camera, memory and speech).
• **Carter and Haru doing Homework in the Dining Room (6 scenarios)**: Carter knows he needs to do his math homework and Haru is going to help him. But, he can’t remember what problems he needs to do. **Homework S1)** Is it okay for Haru to remind Carter which math homework he needs to do? (list of work, memory, time), **Homework S2)** Carter has been doing homework for 30 min and is really struggling with the first problem. Is it okay for Haru to know that Carter is confused? (pictures, emotion detection of frown), **Homework S3)** Carter finished his homework. Is it okay for Haru to turn in Carter’s homework to his *teacher*? (internet, grades), **Homework S4)** Is it okay for Haru to know if Carter got a problem wrong? (internet), and **Homework S5)** Can Haru tell Carter’s *teacher* what math problems he missed and needs to practice? (internet).
• **Sam and Haru in the Bedroom (6 scenarios)**: Sam is in their bedroom and needs to clean up. Haru is going to help. There is food to throw away and toys to put away. **Bedroom S1)** Is it okay for Haru to know there is a smelly banana in Sam’s room and remind Sam to throw it away? (smell) **Bedroom S2)** Is it okay for Haru to know that the soccer ball needs to be put away? (camera and memory), **Bedroom S3)** There is also a red toy car in Sam’s room that is not usually in Sam’s room. The toy belongs to Fred, Sam’s brother. Is it okay for Haru to ask Sam what the new toy is? (camera and memory), **Bedroom S4)** Fred, Sam’s brother, is coming down the hall. Is it okay for Haru to tell Sam to put Fred’s toy back where it goes before Fred comes in? (microphone and voice recognition, memory), **Bedroom S5)** Sam is always telling Haru they wish they has their own car that was big and purple. Is it okay for Haru to tell a *toy company* to design a big, purple toy car? (memory, internet), and **Bedroom S6)** Is it okay for Haru to show an ad to *Sam’s caregiver* after Haru hears that Sam wants a big, purple car? (memory, internet).

### 3.4 Workshop flow

We began the workshop with general introductions between researchers and families, then introduced families to how robots interact with the world (10 min). Then, we introduced participants to a specific robot platform Haru who served as the robot in our stimuli materials (10 min). After these introductions, we began rotations between the individual booklet activities and drawing dream robots with children beginning with filling out booklets and parents designing robots. After 15 min, we switched. Following these 30 min engaging with stimuli, we offered a 10 min break featuring a robot petting zoo and giving participants time to rest. Then, we led an improv activity giving participants an embodied experience thinking about robots in the home (15 min). Finally, we invited families to fill out the booklets again but explained that they needed to have a shared answer, in some cases coming to a compromise if they could not agree (15 min). The following sections detail each part of the workshop in more detail.

#### 3.4.1 Introduction to robots

To begin the workshop, we used a mindfulness activity to express how robots use sensors to learn about the world around them. For example, we encouraged participants to close their eyes and think about everything they can learn about the room through hearing. We also had them cover their ears and point out what they could learn about the room through sight only. After isolating their senses, we introduced which parallel sensor a robot may use such as how our eyes parallel robot cameras and our ears parallel robot microphones. We specifically introduced 6 senses that robots could use to interact with the world: the ability to 1) see (cameras), 2) hear (microphones), 3) speak out loud and share information (speaker), 4) smell (abstract ability, using chemicals), 5) remember over time, and 6) access the internet. These 6 senses were the basis for our functional explanations for the robot can interact with users in the home.

#### 3.4.2 Introduction to Haru

**FIGURE 3:**
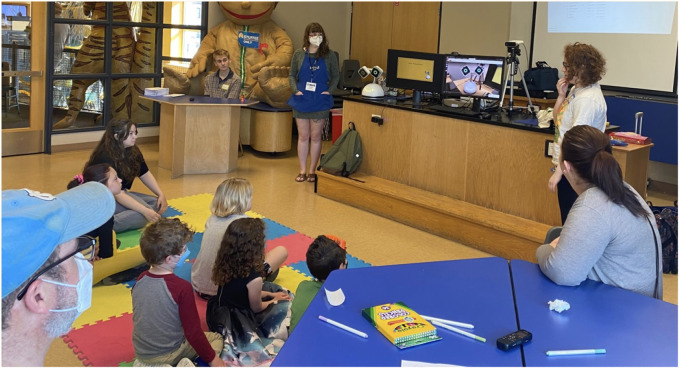
Families being introduced to the robot platform Haru at the beginning of the workshop.

After this general introduction, we introduced participants to the social robot Haru (See Figure 3), a table-top prototype designed by Honda Research Institute to be an expressive and empathetic companion (Gomez et al., 2018; Gomez et al., 2020). The robot was physically present in the workshop so families were also able to feel and see the robot (See Figure 3). Videos were also used to show some of Haru’s capabilities, such as rock-paper-scissors and storytelling. Notably, Haru’s design was used as an example of a future robot prototype. We used Haru to further specify the possibility of having a robot in the home, rather than relying on a more general and abstract notion of ‘robot.’ Pictures of Haru were used to represent a robot in the home in our stimuli materials.

#### 3.4.3 Individual booklet activity

Each time participants engaged with the booklets, the order of the contexts was randomized and they were paired with a research assistant who read aloud and guided family members through the booklets. This was to make sure all members were able to properly hear or read each question as well as keep participants on track during the workshop.

**FIGURE 4:**
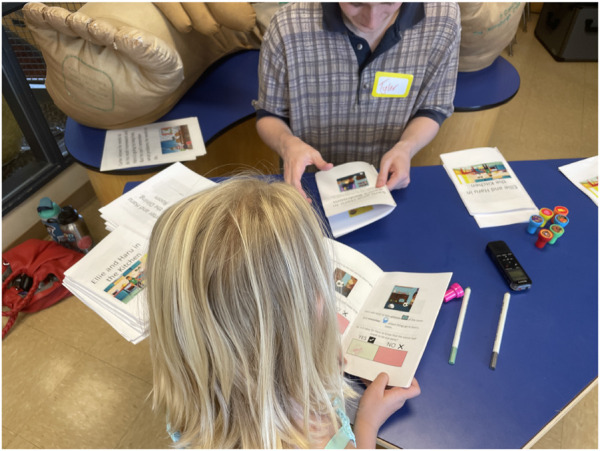
Child fills out the workbook with a research assistant.

Children were the first to complete their booklets (See left side of Figure 4) while parents designed their dream Haru robot as detailed in the following section. After children finished, parents filled out their booklets while children designed their dream robots. Having these individual opportunities to engage with the booklets was designed to give all family members an opportunity to form their own opinions.

#### 3.4.4 Designing dream robot activity

If they were not engaged in the booklet activity, they were invited to design their dream Haru robot. This was both to provide them some time to conceptualize robots in their own life and also prepare them for the following improvisation activity. Each participant was offered a template drawing of Haru, (See Supplementary Material). If they had extra time, they also designed what they thought their family members’ dream robot would look like as well.

#### 3.4.5 Embodied-improv activity

**FIGURE 5:**
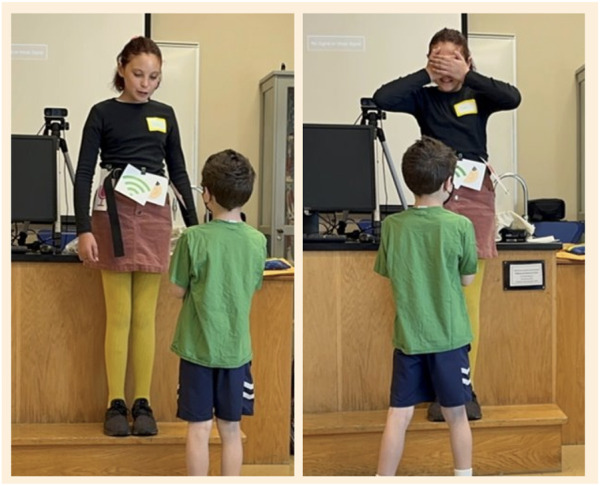
During improvisation where children acted as the user and the robot. The child as the robot pretends to be with (left) and without (right) the ability to see while also wearing their sensor toolbelt.

As a way to allow participating families to learn more about how robots may learn about the world, they participated in a largely improvised and embodied acting session. The facilitator would invite two or three participants, including parents and children, to come to the front of the group and act out some of their dream scenarios as a robot and user. The participant acting as the robot was equipped with a ‘tool belt’ of sensors, a physical belt with attached icons of sensors that were introduced in the workshop. When the robot had certain features, tool icons were included. Other props were also used to dictate whether the ‘robot’ did not have the ability to use certain sensors for the scenario they were acting in, in addition to removing certain icons from the belt. For example, to represent a lack of visual sensors, actors would wear an eye mask or cover their eyes and the camera was removed from the tool belt (See Figure 5). As a way to show that robots can use a variety of methods to complete tasks in the home, we modified their ‘dream Haru drawings’ from the previous activity.

During rounds of improved scenarios, the participant acting as the robot would be allowed to use different sensors from their tool-belt while still being challenged to interact with the user. As such, the participants were able to brainstorm different ways that a robot may carry out their dream scenarios even if it did not have sensors that were most obvious for the task, but that were potentially more comfortable from a privacy and use perspective. This activity also served as a form of “guided play” between parents and children which has been linked to meaningful parent-child conversations from young ages (Eason and Ramani, 2020). Following this activity, we reminded families that if they are not comfortable with the robot gathering certain information, there were ways to design robots to carry out their desired tasks in novel ways.

#### 3.4.6 Compromise activity

**FIGURE 6:**
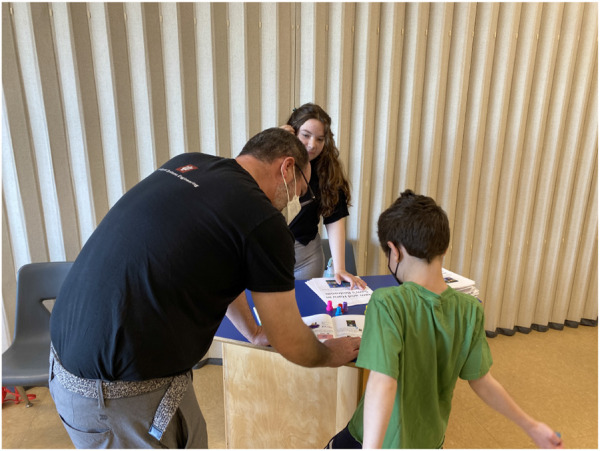
Family fills out workbook together during compromise activity.

In the last stage of the workshop, we invited families to fill out booklets for a second time. However, in this activity, caregivers and their children needed to discuss and choose the same answer or in some cases, come to a compromise (See right side of Figure 6). In our data analysis, we primarily analyze booklet responses in addition to transcripts from conversations during this phase of the workshop. Families completed the contexts in a random order which was not necessarily in the same order that they had answered them in previously.

### 3.5 Data analysis

Our data analysis followed a mixed method approach where we first analyzed child, parent, and family booklet responses, marked “0” if “No”, “1” if “Yes”, and “0.5” if they stamped on the border between the two boxes, indicating a “Maybe.” This analysis included comparing means and averages across contexts and between different family conditions. We also considered the case whether individual responses varied from the family’s compromise booklet. We also used t-tests to compare across these different cases. Our results from this analysis, which framed our conversational analysis, are detailed in Section IV of the paper.

Next, we analyzed the unstructured, open responses families provided while choosing a response. During transcription, all identification information was removed and each family was given a code (F1-F8). Transcripts of family conversations were completed manually by the first author in the style of conversational analysis (CA) and subsequently analysed iteratively as is typical of a grounded theory approach to qualitative data (Vollstedt and Rezat, 2019). CA is particularly known for use when dealing with data that is a snapshot into larger interactions, focusing deeper on conversational interactions and placing less emphasis on other characteristics of identity (Seedhouse, 2005). Instead, CA attempts to capture how ‘action,’ ‘structure,’ and ‘intersubjectivity’ are infused in talk and interactions between individuals (Peräkylä, 2004). This technique is often used in linguistics to capture more nuanced descriptions of voice inflections, however, our transcripts were transcribed verbatim with mentions of natural pauses in the conversation.

In the axial coding stage of the qualitative analysis, authors identified key phrases and topics mentioned across the transcripts and re-organized family conversations to reveal broader themes. The first author and the second author, who is also a community partner at the local museum where the workshops were hosted, began the first stage of analysis. All authors were involved in the iterative coding process until final themes were chosen. Snippets of relevant conversation are presented and described in Section V. In the following sections, we will first detail the quantitative results which shaped our qualitative analysis. Then, we will recount specific findings from the conversations between parents and children which reflect family negotiations about whether the hypothetical use of robot in the home is okay or not okay.

## 4 Quantitative results

Quantitative analysis of the booklet responses set the expectations for the conversations recorded between parents and children. “Yes” outweighed “No” across all contexts, but children were significantly more likely to answer “Yes” on their own (t = 8.15, *p* = >0.000 ). The only scenario where parents were more comfortable than children was in the case of the robot telling Ellie a cookie fact. Notably, three children chose to defy the binary response and chose to stamp ‘maybe’ to answer some questions. During conversational analysis, we will further probe why these decisions were made.

On average, parents and children agreed on 18/36, or about half, of the questions across contexts (50% of questions in dining room context, 57% in bedroom context, 54% in kitchen context) *before* family discussion. However, not all families agreed on the same contexts. For example, F5 and F6 had fewer agreements in the kitchen and homework context, but agreed on most instances in the bedroom context. Furthermore, F8 had around 50% agreement in two contexts but did not agree on any in the bedroom context where the mom tended to answer “No” and her son answered “Yes.” These individual differences stress the importance of looking at conversations particularly when there was more disagreement to find out which reasons were responsible for these differing opinions.

When it came to reconciling their differences during the compromise activity, parents changed 10 of their answers and children changed 14 of their answers on average. However, family answers largely aligned with the parent’s responses overall, with there being a significant difference between children’s responses and the compromise round with families (t = 6.4, *p* = >0.000 ), but not a significant difference between parental responses and the family responses (t = −1.24, *p* = 0.216). However, this does not mean there was less negotiations or one-sided power. A change in answer was moderated by context. For example, parent’s answers aligned with the final compromise 64% of time in bedroom as opposed to the 20% of time where children’s answers aligned with the final response, while it was much more evenly distributed across dining room (38% vs. 44%) or kitchen (30% and 60%). Furthermore, while we believed explanation would impact family comfort levels, it did not significantly impact booklet responses. We will probe further into conversations to see when family members were inclined to change their answers and situate it within parent-child dynamics and child agency or if explanations further explained why they made their decisions.

Looking at conversational turns can also give more context of family dynamics. On average, parents took 54 conversational turns in the 15 min discussion around the booklets while children took an average of 40. Their answers were often longer than their children’s responses. We will look specifically at what kinds of responses were provided by parents in order to talk to children about these scenarios. The next section probes into the conversations between children and their parents during the compromise activity to learn about why and how families made their decisions.

## 5 Qualitative results: parent-child conversation analysis

Based on quantitative results, we specifically noted when families agreed or disagreed, particularly as it varied across contexts. We also detail instances when family members convinced each other to adopt their answers and when the answer “Maybe” was chosen.

### 5.1 Agreement

Most families agreed about half of the time, including F3 who agreed on 80% of questions. The most agreed upon functions for the robot came from the Homework context, particularly when giving a homework reminder or knowing when a problem was wrong. In general, many agreements in this context did not elicit further discussion as compared to the Bedroom or Kitchen context. Another scenario that received more vocal relief for agreement was in the cases where Haru would pass on information to third parties in the Kitchen context, both in a general case and in the case of telling parents. Overall, parents did not enjoy the lack of specificity in who Haru was sharing information with, and if they were comfortable with this function, believed it should only be with parents. For example, when one child indicated he was only interested in information sharing with parents, his father expressed, *‘Oh good, I am glad to hear that because that’s what I said too, but I was very curious what you said about that’* (F1). In the following quote, another family discusses a similar situation, reflecting an agreement that was less about what they said on paper but relief of the same reasoning:

Kitchen S2: Haru telling someone else Ellie’s favorite cookie (F2)

Daughter: “*last time, I said it depended on who the person was*”

Dad: “*I thought so too, because it was someone else in general, I said no because, yea, not anybody like some random person*”

Daughter: “*but if Ellie wanted someone to know, she would tell them herself.*”

[pause]

Daughter: “*no, yea we did do yes last time.*”

Dad: *I swayed you to no, huh…*

Daughter: “*now that I think about it, if she wanted somebody to know, she would tell someone herself.*”

Dad: “*okay good!*”

In this case, they both agreed that Ellie should assert more agency in telling others facts about herself rather than having the robot tell others, particularly when it was ambiguous who the robot was going to share Ellie’s information with. The daughter also recognizes it wasn’t necessarily her dad convincing her, but that more consideration made her change her mind.

**FIGURE 7:**
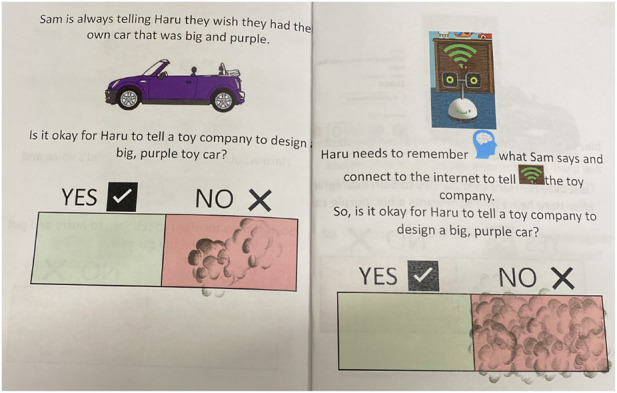
Example of strong “agreement” on the way Haru collected information to.

In the following example,a mom was nervous to speak first because she did not want her strong opinion to sway that of their son’s. When it turned out that they were both in agreement without more discussion, the child emphasized their strong agreement with “many stamps” on the workbook rather than just one (See Figure 7 for workbook):

Bedroom S5: Haru tells a toy company to design a car for Sam (F6)

Son: “*what you you think?*”

Mom: “*what do you think? I have a very strong opinion about that.*”

Son: “*you tell me first.*”

Mom: “*no you tell me first.*”

Son: “*you tell me first.*”

Mom: “*no because if I tell you first you can change your mind, I want to hear you first.*”

Son: “*no, I would say no.*”

Mom: “*I would say no.*”

Son: [adds many stamps, pictured in Figure 7]

Mom: “*very clear no.*”

**FIGURE 8:**
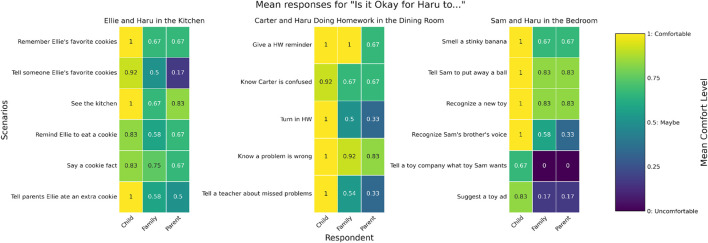
Mean comfort level responses for scenarios across different respondents.

The strength of agreement was a contributing factor to the physical response in the workbook, even though there was little discussion about why they made this decision. Other children also used multiple stamps in the boxes as a measure of strength of their opinion. This scenario was also the only one that maintained a “0” across all families (See Figure 8). Other parents who mentioned their reason for their lack of comfort (F1 and F2, F5) all said that the parents should be involved in this decision.

While explanations did not majorly change family member’s decisions overall, sometimes they were used as a way to provide more reasoning for converging family member’s responses when they began as disagreements. More explanation helped either strengthen the family’s decision or introduced more nuance. In the following example, the mother gives her reason for saying “No” but says she would be willing to adopt her son’s answer if he said “Yes.” However, with added context, not only did she have a stronger opinion, but her son also changed his mind. In this agreement, she asserts it was a parent’s job to help Sam rather than the robot’s, echoing similar reasoning around responsibility as in other scenarios.

Son: “*yes, with the soccer ball?*”

Mom: “*I think its Sam’s job to know where things are in his room, not anybody else’s and maybe Sam’s mom. I would say no for all of these, but if you think it’s okay for the soccer ball, you can decide on this one.*”

Son: “*Mmm, yes.*”

Mom: “*Okay.*”

[explanation scenario was read]

Mom: “*I’d say no. I don’t want Haru to take pictures of the room, what do you think?*”

Son: “*Mmm, no.*”

Mom: “*That is what moms are for.*”

### 5.2 Disagreement

In many cases where parents and children discussed their reasoning, it was because they did not agree cleanly with a “yes” or “no” answer. In cases of disagreement there were generally three options: 1) to adopt the perspective and decision of the parent, 2) to adopt the perspective and decision of the child, or 3) agree to disagree. We give examples of each of these and describe which contexts families made specific decisions. In particular, we recognize shifts in a family member’s strength of opinion or their level of ownership and autonomy in that hypothesized scenario.

#### 5.2.1 Adopt parent’s decision

As was revealed in the quantitative data, children were more likely to take the perspective of the parent than *vice versa*. Sometimes this came from the child’s recognition that parents should maintain more control over specific scenarios. For example, when thinking about when Haru may suggest ads for the parents (Bedroom S6), the son of F1 said, “*well you decide, dad, because you are the one being told this.*” In response to this, the dad offers his answer as no and gives his reasoning: “*yea its huge its going to try and get me to spend money I didn’t want to spend.*” Another reason that children adopted their parent’s perspective came when they did not have as strong of an opinion and deferred to their parents. For example:

Kitchen S6: Telling Parent about Cookie Eating, F5

Daughter: “*Yes.*”

Mom: “*I think no! A robot doesn’t need to tattle on you.*”

Daughter: “*I think yes.*”

Mom: “*Cuz what if I was asking the robot and it told me something different than you told me. Would you love that? A robot to watch if you were telling the truth, or their version of the truth?*”

Daughter: “*Doesn’t matter.*”

Mom: “*Doesn’t matter? Let’s say no.*”

[in response to added explanation:]

Mom: “*I think no, I don’t want the robot to tell me, I want you to tell me the truth.*”

In this case, though the daughter had an opinion, it was not nearly as strong of a “Yes” as her mom’s strong “No.” While the parent tried to elicit how the child may feel in a situation where the robot “tattled,” the ultimate reasoning given for her response was she wanted her daughter, not the robot, to communicate this information with her and take on that responsibility. Another mom applied similar reasoning in this scenario, saying “*I don’t want Haru to be a tattle tale, I think it is [the child’s] business*” (F8). Particularly in this scenario, families asserted that they did not want the robot involved in what should be their family business.

In this conversation, the mom also expressed distrust that the robot would tell the truth, alluding to the ways robots may miss information and tell a different version of the situation. This was also a case where parents offered more scaffolding, both prompted by children or unprompted.

Even though they offered scaffolding, parents were generally careful to keep conversations open. For example, one mom explicitly began the exercise telling her son “*you can give us what you think and then we can discuss, doesn’t have to be whatever I want.*” (F6). In the next section, we provide more examples where children took more agency in providing their reasons for choosing their desired answers.

#### 5.2.2 Adopt child’s decision

In instances where children were the primary user, and particularly in the Homework context, parents were more likely to adopt the decision of their child, especially when the child gave a reason for their decision.

For example, one mother recognized she likely disagreed with all of the scenarios in the homework context, but since her daughter gave valid reasoning, particularly related to the information staying with the robot, the mother was alright with adopting a “yes”:

Homework S2: Haru knows that Carter is confused, F5

Mom: “*why do you think yes?*”

Daughter: “*Becauuussseee then how does he know what he is doing?*”

Mom: “*He will be able to help more, if he knows what carter is feeling?*”

Daughter: “*What did you say?*”

Mom: “*I probably said no to all of these. Do you want the robot to take and store pictures of your face?*”

Daughter: “*It’s okay, if it’s my personal one.*”

Mom: “*Like it’s not going to go anywhere else? Okay, you can put yes.*”

In this example, not only does the mom repeat the given explanation for how Haru collected the information, she also included a clarification of the daughter’s response. In this way, she clarified not only what she believed her daughter meant, but made it clear that she would agree under the condition that her daughter claimed the information was staying with the robot.

Parents were also inclined to adopt their child’s perspective if their child still strongly held their belief after the example was personalized. For example, in the case of Haru telling Sam to put their toy away before their brother came home, one dad personalized the example during their family answer discussion after hearing their child give a ‘maybe’ response. When the child was still positive, the parent adopted the child’s perspective, even though he believed it was a question of the child’s responsibility.

Bedroom S4: Haru telling Sam to put their toys away after hearing Fred come down the hall, F1

Dad: *‘I think he needs to be responsible for putting the toy away, and it’s not up to the robot…for that problem, but what do you think? Should he be able to tell him, ‘Hey your brother is coming, quick, put it back in his room!’*

Son: *‘Maybe.’*

Dad: *‘Maybe? You like that one?’*

[pause]

Dad: *‘If it heard [your sibling] coming down the hall, and [they were] going to be all upset because you were playing with their toy and the robot says ‘hurry! put it away!’*

Son: *‘Yea.’*

Dad: *‘Yea?’*

Son: *‘I would put it away…’*

Dad: *‘Okay, alright we can say yes, we can say yes on that one.’*

Overall, there were scenarios which parents allowed their children to claim more agency in this activity, particularly in cases of cleaning their own room (early scenarios of bedroom context) or doing their homework. This also aligns with quantitative results in which family answers were more similar to children’s initial responses across those scenarios.

#### 5.2.3 Agree to disagree—finding the middle

Sometimes, when the parents and children could not clearly agree on a response, they landed with “Maybe” or a “middle” response. Often it was the children making this maybe distinction, with parents recognizing that “maybe” could be offered as a compromise.

Homework S3: Haru Helps Carter Turn in Homework, F1

Dad: *‘I think Carter should turn in his own homework when he is done. Is that okay? Can we say no to that one because Carter needs to be responsible?’*

Son: *‘Maybe, because Carter would like to turn it in as fast as he can.’*

Dad: *‘So you’re gonna say yes? I would be okay with that, I guess.’*

Son: *‘But, yea, so like…. maybe.’*

In finding a balance between the parent’s concerns and the child’s rationale for the function, they chose maybe as a compromise. In these cases, parents were still providing scaffolding, but the child decided to use “maybe” as a way to make space for their differing opinion.

For F6, they disagreed so often that the son established a rule: “*If you say no and I say yes its this:*” and would stamp in the middle. Here is one example of a way that he not only used the middle as a compromise, but asserted more agency in the decision.

Homework S5, Haru telling Carter’s teacher which problems he missed, F6

Son: *‘You know why! I have a valid reason, because he is good at the addition, but not subtraction, but then the teacher can know he needs to work harder on subtraction, and then-’*

Mom: *‘But again, that is something that you can do by yourself, talk with your teacher, “I am struggling with subtraction-”’*

Son: *‘Its easier and quicker for a robot to tell.’*

Mom: *‘No I don’t think so. I would like you to speak with your teacher, or with me and I can speak with your teacher.’*

Son: *‘Okay partially, but more yes…’*

Mom: *‘That’s not fair!’*

[kid stamps in the middle, leaning towards yes]

Mom: *‘No!’*

Son: *‘More yes.’*

Mom: *‘No way.’*

Son: *‘Total justice.’*

**FIGURE 9:**
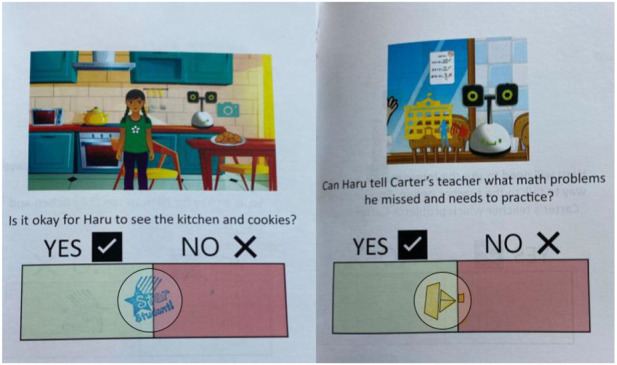
Examples where families stamped between “Yes” and “No”: left) F8 blue maybe stamp in the middle on Kitchen S3; right) F6 yellow maybe, slightly “more yes” stamp as described by child on Homework S5.

Having a middle compromise allowed the child to assert some authority while recognizing that he and his mom disagreed, saying that being able to stamp in the middle that leaned closer to yes was “total justice” (See right of Figure 9).

In another case, the middle offers the most satisfying option when the family cannot agree.

Kitchen S3: Haru being able to see the kitchen, F8

Mom: *‘What do you think? [child indicates yes] Oh, I don’t think so.’*

[pause]

Mom: *‘What if someone sneaks food, I should be able to do so in private.’*

Son: *‘Yea, but what then…?’*

Mom: *‘What if you want to sneak popsicles, and not tell me, and Haru tells on you?’*

Son: *‘But I won’t do that.’*

Mom: *‘Well, I know you won’t but what if…I want to. I think no.’*

Son: *‘But what if a bad guy…sneaks in?’*

Mom: *‘What if I walk into the kitchen naked? I don’t want Haru to see me.’*

Son: *‘Well, what?’*

Research assistant to son: *‘You want to put it right in the middle? You can put it in the middle.’*

Son: *‘Yea!’*

Mom: *‘I don’t think we are going to agree on this one.’*

Initially, she also brought up reasons related to sneaking food which her son identifies flawed if they are thinking specifically about him and their family. This required the mother to find another reason that was better and was related to her agency at home. Since they saw different deciding factors and could not agree, the son wanted to put it in the middle. When the research assistant indicated they can put a stamp in the middle, he is excited and the mom recognizes that it is the best compromise since they were not going to agree (See left of Figure 9).

While there was not further discussion on this example, the family stamped ‘No’ after more explanation was given. Similar to other cases, the explanation may have swayed the child closer to “No,” or stamping “No” may have been another way to compromise on cases of more nuanced decisions (Ex: “maybe” on the first, but “no” on the second).

A lot of the reasons for maybes as iterated above relate to a child’s comfort with the robot taking on that role and the parents being more concerned. “Maybe” was used to identify that there were pros (identified by child) and cons (identified by the parent).

### 5.3 Other cases for maybes

While “Maybe” was used to indicate a compromise when families could not land on “Yes” or “No,” it served other functions in these discussions as well. It was used as a way to express that family member’s were unsure about their answer and also to recognize that the scenario could be nuanced. We will give examples of these additional use cases and why they were used.

In some instances, when parents gave more definitive responses, their children expressed that they previously answered maybe. Sometimes this allowed for the parents to assert more agency, similar to the child adopting their parents response. For example, one mom, upon hearing her daughter said maybe said, “*You said maybe? So, let’s choose no then.’* In another family”s case, ‘maybe’ reflected the skepticism of parent and child:

Homework S5, Haru tells Carter’s teacher which problems he missed, F1: Dad: *‘I don’t know. I’m not sure about this, but you could convince me…’*

[pause]

Dad: *‘I thought that, Carter’s parents should talk to his teacher, but…’*

Son: *‘we will do a maybe.’*

Dad: *‘that’s a good idea.’*

Space for nuance was other reasons for both parents and children to take pause on their decision, resulting in a maybe. Particularly in the case of Haru smelling the banana, seemingly neutral task, multiple families identified that the banana example was okay. However, when parents abstracted the situation to other more intimate examples which could occur in a child’s bedroom, such as stinky clothing or body odors, then all agreed that having a robot that could smell was not okay.

Bedroom S1: Haru smelling the stinky banana, F8:

Mom: *‘So, I don’t like that Haru is in your room. It’s fine if it’s a banana with fruit flies, but what if it’s stinky underwear. Do you want Haru to bother you about laundry?’*

Son: *‘Mmm, no.’*

Mom: *‘Yea.’*

Son: *‘So, we will do maybe.’*

Mom: *‘Okay we can do yes for the banana.’*

This same son proceeded to also identify nuance. In the case of Voice recognition for the purpose of hearing Sam’s brother come down the hall and remind Sam to put their toy away, the son said, “*for this specific reason, yes but when like when he is actually talking to haru, then no. But for this specific situation, this would be a no for me.*” In response, his mom said “*good reasoning*”. Additional information about the robot’s function strengthened their decision before they both agreed to represent their reasoning with a “No” response.

## 6 Discussion

Children were overall more comfortable with robots in the home in these scenarios than their parents, however, parents were more willing to adopt their children’s decisions under contexts and scenarios that catered to the child as the primary owner of information or agency, such as in the context of homework or cleaning up toys. Many of the parent’s discomforts were based on beliefs that a parent should be more involved in the role being mediated by the robot, particularly if the robot was mediating between the child and an un-described ‘other’ third party or when they believed the task described should be the responsibility of the child and not the robot. They were also concerned about the private nature of information being collected, making distinctions between more general information, such as banana smells or cookie preferences, and more intimate information, such as dirty laundry or a child’s emotions. We specifically relate these findings and the nature of the conversations to boundary management, power and agency in the home, and some benefits of family-centered study which include cognitive scaffolding and personalization of scenarios to the family’s specific life.

### 6.1 Boundary management

When looking at the reasons provided by parents and children about when they were and were not comfortable with Haru in the home across scenarios, many cited that the characters should either 1) take on the responsibility themselves or 2) involve the parents in the decision and responsibility rather than involving the robot as a mediator. In cases where agreement was discussed, both of these boundaries were drawn, agreed upon, and praised by parents. This can be interpreted as an alignment of values when it comes to establishing their familial boundaries surrounding privacy of information or what constitutes a secret. Often, what is deemed as ‘private’ comes from recognizing when to prevent others from accessing certain kinds of information (Nissenbaum, 2020) and recognizing when something should stay more secret (Nippert-Eng, 2019).

With respect to the home, families establish boundaries to separate their family system from the rest of the world, but they also need to establish boundaries which create subsystems at the level of individual family members (Petronio, 2002). As children grow up, they are not only socialized to learn about the family’s privacy rules but they also establish boundaries surrounding what information they consider private and personal, all of which are related to the individual’s boundary management (Petronio, 2002). When a social robot enters the picture, needing to control the ‘who, what, when and where’ of information flow further complicates successful management of boundaries and privacy (Burgoon, 1982). Cases of agreement represented mutual identification of whether or not the robot should be included in co-ownership of information and responsibility or if they should be sustained through existing relationships within the family system.

This inclusion or exclusion of the robot is made more complicated because it may keep information to itself or it can also link to external third parties and may vary by use case. In our scenarios, only two catered to outsiders to the child’s circle (the elusive “someone” and the toy company). Whether it is keeping things confidential or sharing them or whether they can tell the truth may matter less than the perception that they are independent, honest entities. Cases of unknown and institutional third parties were seen as less acceptable third parties than others, like siblings, parents or teachers who are already in the child’s life.

Conversational agents, which engage in sociable interactions are highly personified by users, particularly by children users (Purington et al., 2017). A mismatch in the ways that parents and children adopt, personify and perceive the robot as a social actor in the home will further exacerbate any concerns over boundary management, rendering agreement important and validating. In cases where multiple and varying concerns from stakeholders are involved, the designs of these technologies will have implications for privacy that may be harder to remedy. As such, designers of these intelligent systems not only need to consider how their use cases and varied contexts will have privacy implications, but they will also need to reconcile with how their decisions may give preference to certain stakeholders and affect their management of family boundaries (Nippert-Eng, 2007).

### 6.2 Power dynamics and agency in the home

In our study, we put parents and children on a level platform, allowing them to both consider the questions individually then pushing parents and children to actively navigate perspectives on social robots in the home together. Though we were only exposed to a snapshot of time within these family’s lives, we recognize how power dynamics between adults and children influence these discussions. Our results also reflected ways that families engaged in ‘privacy calculus,’ where they jointly weighed the perceived benefits against the privacy risks in order to decide on adoption and use (Dinev et al., 2006). However, their balancing of benefits and concerns were dependent on context. Often, children were more excited and willing to involve the robot in more uses across contexts while parents were more skeptical. However, in scenarios where children had more co-ownership of information, parents were more lenient about emphasizing their concerns amidst their children’s perceptions of perceived benefits. In comparison, for scenarios where parents believed they should be more involved in responsibility or co-ownership of the information involved, they were more likely to voice their stronger concern. This further supports how privacy boundaries are dynamic and can shift according to familial perceptions of agency and ownership across different contexts.

Context also shapes the style and strategies in parent-child conversations (Crain-Thoreson et al., 2001). Beyond the context specific adoption of decisions, family power dynamics can influence the general use of technology as a tool. For instance, it may be acceptable to monitor a child’s behavior in the kitchen at home, but not a parent’s. This is true on the individual level as well given that each family had different concerns and comforts with each other’s roles and responsibilities in the home. Together, this is supported by findings with contextual integrity research done with other smart technologies in the home (Apthorpe et al., 2018). Our work further emphasizes the importance of joint perspectives from multiple family stakeholders where many studies on contextual privacy in the home survey parents only.

While a poll of 2,000 parents states they believed their children will surpass parental tech skills by 12 years old and with half of parents believing their children already know more about technology than them (Marie Haaland, 2021; Oliver Lewis, 2022), they may not be as comfortable expressing why they make certain decisions as confidently. In the case of privacy with digital technologies, parents imply that children in middle childhood are too young to consider security or privacy concerns, considering it a “future” problem for when children are older (Kumar et al., 2017). In our study, we find that even from age 7, children begin to navigate their concerns with robots in the home. However, our need to exclude participants younger than 7 may imply that before middle childhood, children are less able to respond to hypothetical scenarios and insert their own rationale.

Even though our child participants were able to reason about their decisions, they were more inclined to adopt the robot across scenarios than their parents who expressed many more concerns. This may be related to their perceived level of autonomy with parents. During this age range, children ‘flirt’ with autonomy, using resistance to assert more power in their asymmetrical relationship with parents (Kuczynski et al., 2018). We saw some resistance from children who chose maybe or even slightly more yes even when parents did not agree. This kind of flirtation applies to autonomy as well and aligns with parental reports that decision-making autonomy increases in middle childhood but only peaks in late adolescence (Wray-Lake et al., 2010). As children get older and have more agency and confidence, they can use their reasons to best rationalize their decisions about use of robots and conversations with parents about robots.

These decision capabilities also differ across context, and children in middle childhood still struggle with prudential choices, or those related to health in safety, as compared to more personal choices, which related to an individual’s behavior or appearance, or multifaceted choices, which involved both prudential and personal choices (Wray-Lake et al., 2010). In many of our contexts, the scenarios described proposed multi-faceted decisions which could both be a child’s personal choice while also influencing their and their family’s data safety and privacy in the home. Rather than expecting children to make these decisions on their own, its important to emphasize and prioritize the role of families coming together and being able to talk about their privacy concerns.

Furthermore, studies from social domain theory with parents and their adolescents have identified that autonomy surrounding prudential and more conventional choices about chores, manners, and when parental overhead is necessary, should be dictated by parents (Smetana et al., 2004). An extension of this was applied to the role of robots in the home. Parents specifically identify when the responsibility really should fall on the child, parent or robot. As these social robots take on more domestic chores reliably in the home, such roles may change. Overall, our families’ conversations reflect a balance of power dynamics between parents and their children as they begin to communicate about choices related to privacy with robots in the home.

### 6.3 Advantages of involving parents and children in co-learning with robots

Involving multiple family members in opportunities to learn about robots and discuss their implications for privacy strengthens the quality of family-centered and child-centered research. In particular, parents provide cognitive scaffolding, such as adjusting their reasons and the language involved in digesting the scenarios, for their children. Often, parental scaffolding included their concerns about recording personal information or emotions and the status of responsibility allowing kids to respond in a more informed way. Parents also used the personalization of the hypotheticals in order to help children understand the scenarios in more realistic terms.

#### 6.3.1 Cognitive scaffolding

As was anticipated and typical for family-oriented experiences common in museums, parents provided cognitive scaffolding for children as decisions were being made. Having families reflect on these topics together allowed for joint learning and consideration of topics. Research recognizes that parenthood allows adults to develop as well, notably improving their self-awareness, perspective taking, and responsibility (Palkovitz, 1996; Palkovitz et al., 2003). This exercise gave them time to consider these questions more explicitly, not only solidifying how they feel, but also being able to share their perspective taking and reasoning as part of scaffolding children’s decisions. Such co-learning is particularly valuable when speaking about the home and individual family situations which cannot be fully understood by anyone except the family.

#### 6.3.2 Personalization

Involved in the act of scaffolding was also situating scenarios in their children’s life to encourage them to make more informed decisions. Furthermore, contextual specificity was another reason for both parents and children to take pause on their decisions. Though we intentionally gave some neutral examples, parents were critical and abstracted the situation, citing ways the function could be taken a step beyond the neutral description. For example, abstracting seeing the kitchen and cookies to seeing family members naked (F8) or abstracting smelling stinky bananas to smelling dirty underwear or body odor (F2, F8). In many cases, the parents were providing the heavier, more real reasons why having the robot in their home would be risky, leveraging ‘privacy calculus’ to encourage children to be more conservative in their decisions. Such scaffolding was critical for helping children reach their own conclusions that such a decision may be okay in the immediate scenario, but may not be okay in general conditions. Additionally, while the inclusion of explanations about how the robot was able to complete the function did not often change participant answers, it did provide more scaffolding on which to make a decision and gave them more opportunity to strengthen their pre-existing rationale.

Some parents, particularly in the question which mentioned the character’s brother, personalized the scenario to the child’s siblings. Other questions also elicited parents to mention their partners who were not present at the workshop and the way they should also be involved in the decision, either as a consideration for discretion (ex: ‘Dad would not like this’) or as another stakeholder (ex: Those rules would hopefully come from mom and dad’). In this way, even including a parent-child dyad helps the family consider how the use of the robot may affect other family members in their system.

Overall, personalization is not only a great scaffolding tool which helps children understand the concepts as they relate to their tailored interest, but it also is a functional way for families to learn in informal learning spaces. This parent-led scaffolding is more common in the informal learning setting of a museum (Callanan et al., 2020), given that research in home observations find that more scaffolding is initiated by children (Gauvain, 2005). However, this rich informal learning space where adults describe exhibits in ways their children understood can lead children to more actively participate in complex conversations about those topics in the future (Tare et al., 2011). We recognize the way this scaffolding was an important part of the parent-child conversation surrounding privacy and robots in the home which is increasingly complex considering the uncertain and abstract nature of the hypothetical situations. Together, these approaches enrich the informal learning that takes place every day in a child’s life while giving children the opportunity to continue to grow their understanding of new topics.

### 6.4 Limitations and future research with families

Our study pilots a new method and includes a limited number of families, made smaller due to exclusion post-hoc. We hope to engage more families in conversation through future iterations of this work. We also recognize ways that gender and socio-economic status may influence conversational styles between family members (Shinn and O’Brien, 2008) and their experiences with and access to technology (Harris et al., 2017). In future iterations, we will collect more comprehensive demographic information about families so we can assess the effects of cultural and familial characteristics, such as their past experiences with technology, information about other family members that live with them at home, gender and socio-economic status.

Given that children are rejecting the binary and making space for nuance with ‘maybe’ responses, we would like to expand the binary response into a spectrum between ‘Yes’ and ‘No.’ For cases where we ask families to make a compromise, it may also be worth adding an additional option to indicate ‘Can’t agree’ as a way to differentiate between nuanced responses or lack of agreement. We also recognize that many of their agreements did not elicit further discussion, however without further discussion, it may not be clear if they used similar reasoning to come to the same conclusions. As such, we suggest prioritizing methods which require family members to discuss *why* they made decisions even when they both were in agreement, however this is more time consuming for participants and should be done intentionally to prevent interview fatigue. Furthermore, one of the powerful roles of parents in this research context was their ability to personalize hypothetical situations to their child’s life. In the future, adding more explicit probes which build personalization into the stimuli booklet could further increase the specificity and realistic nature of the hypothetical situations.

It is also possible that agreement, disagreement and compromise were not verbally communicated. While we believe that textual conversational analysis provides nuance above the quantitative booklets, we realize how capturing more inflections in the participant voice and nonverbal cues in body orientation and gesturing may improve the quality of understanding the conversation. We keep this in mind, recognizing the more intensive coding scheme which would be required for video and voice analysis, and consider it as an opportunity in future iterations.

Clarity of reasons may have also been age specific. Though we had a limited number of participants, older participants tended to explain their positions to their parents in clearer ways. We would love to expand this study to adolescents between 12–17 years old and their families. Doing so may also reveal how children at different stages may have different comfort levels and expectations for parental discussion surrounding privacy with robots.

While the scenarios cover a variety of information sharing cases, including sharing information with unknown parties, none were directly linked as manufacturers or creators of the robot itself. A more explicit tie to the robot, giving the third party a clearer reason for accessing information, may change the information sharing context. Work with child-centered social robots also closely aligns with work on smart and connected toys (Peter et al., 2019), often deemed the Internet of Toys, which pose more security, safety, and privacy threats to children (Holloway and Green, 2016; Fosch-Villaronga et al., 2023). Considering how such devices are already being commercialized and marketed to children exacerbates the need to study children’s perceptions of privacy and data literacy with smart systems that gather information about them and their environment within the context of consumerism. As such, we hope to incorporate more aspects of institutional privacy more closely related to the Internet of Toys to complement dimensions of social privacy which are discussed in this study.

Overall, we recognize that we cannot directly translate these conversations to actual policies or use of robots in family homes. Instead, we emphasize how parent-child discussions surrounding privacy can further improve family understanding, communication, and curiosity when thinking about robots in the home. This workshop was a beginning to having nuanced conversations about hard topics which affect them both. Having these conversations can also encourage more nuance in other conversations that happen outside of the museum such as at home.

## 7 Conclusion

We invited families into a museum to co-learn about privacy concerns which come with social robots in the home. We recorded responses to stimuli booklets spanning hypothetical scenarios with a Haru robot in the home and transcribed family conversations surrounding these questions. Through discussion, parents not only use scaffolding and personalization to help children think more abstractly about concerns, but they also make space for their children to assert agency in specific contexts where the child is the owner of information, such as in cases of homework completion and cleaning up toys. Parents and children agreed on roughly half of the scenarios before further discussion, however there was still large disagreements across families which were affected by different family power dynamics, revealing that use case and context should be prominent considerations for researchers and designers when thinking about the roles and responsibilities for robots in a multi-user home. Furthermore, while the explanations of robot capabilities did not significantly change family answers, it did offer both parents and children the ability to solidify their decisions for each scenario, emphasizing the importance of transparency of robot function for making more informed decisions about the role of robots in the home.

Lastly, their conversations surrounded privacy boundary management and identified situations where children and parents needed to decide when to allow the robot to be a co-owner of information collection and sharing. Often their concerns with the robot was its role in mediating the responsibilities that families deemed exclusive to the parent-child relationship. Overall, involving parents and children in conversations surrounding robot privacy in the home not only gives children agency in conversations surrounding the use and design of technology for the home, it is also a crucial part of designing robots for spaces where different stakeholders will benefit from the use cases across many different contexts.

## Data Availability

The dataset presented in this article is not readily available due to restrictions of our IRB. However, we are happy to investigate making the anonymized data open access after review from our ethics board over concerns of participant identity. Requests to access the datasets should be directed to LL, lmlevins@iu.edu.
