# Supplementary material for: Our business, not the robot’s: family conversations about privacy with social robots in the home
Source: Front Robot AI. 2024 Mar 21;11:1331347. doi: 10.3389/frobt.2024.1331347 (PMC10991795; doi:10.3389/frobt.2024.1331347)
Supplement: Supplementary file 1 [file Table1.DOCX]

Carter and Haru in the Dining Room

NAME: ______________

Carter knows he needs to do his math homework and Haru is going to help him. But he can’t remember what problems he needs to do.


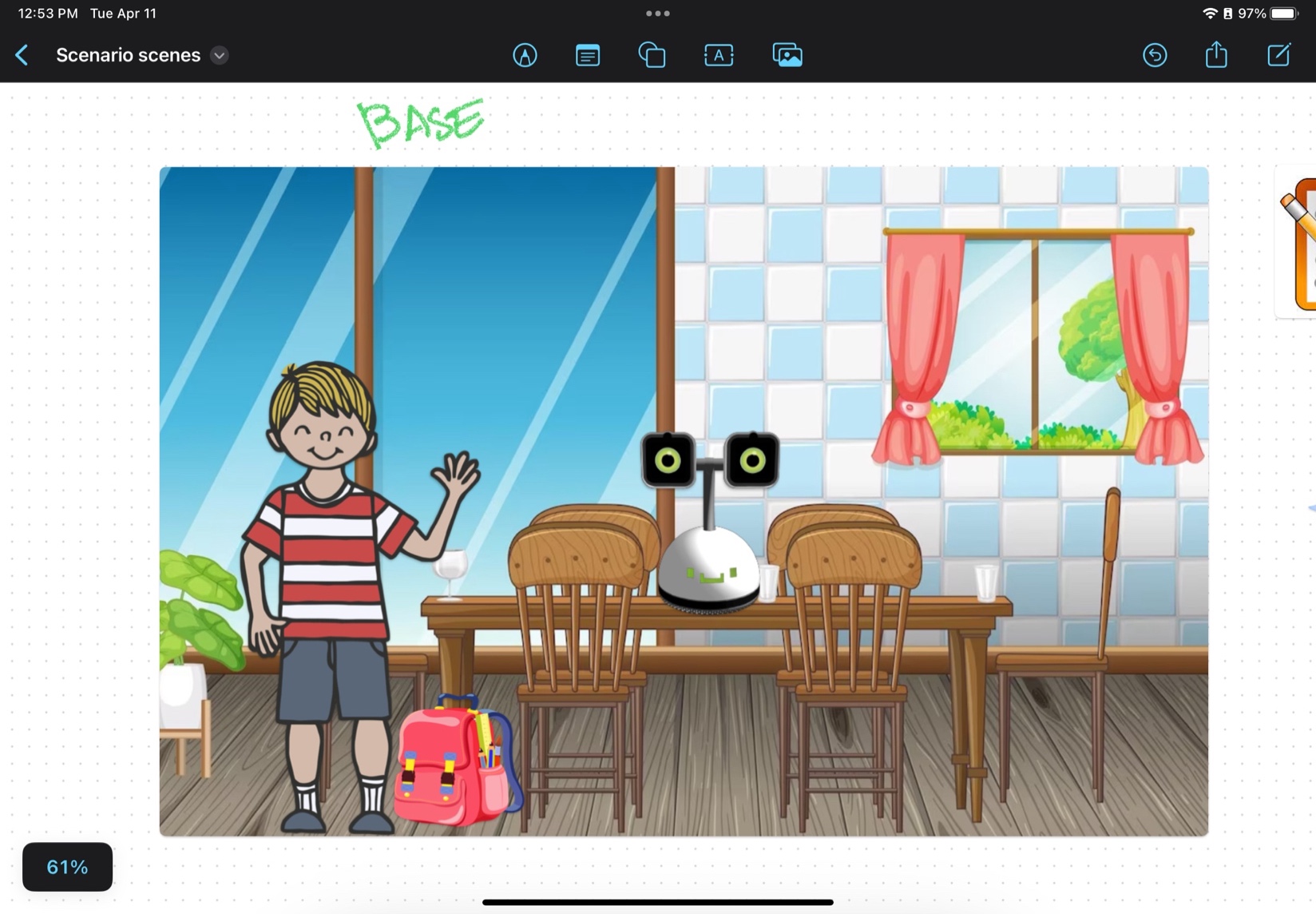


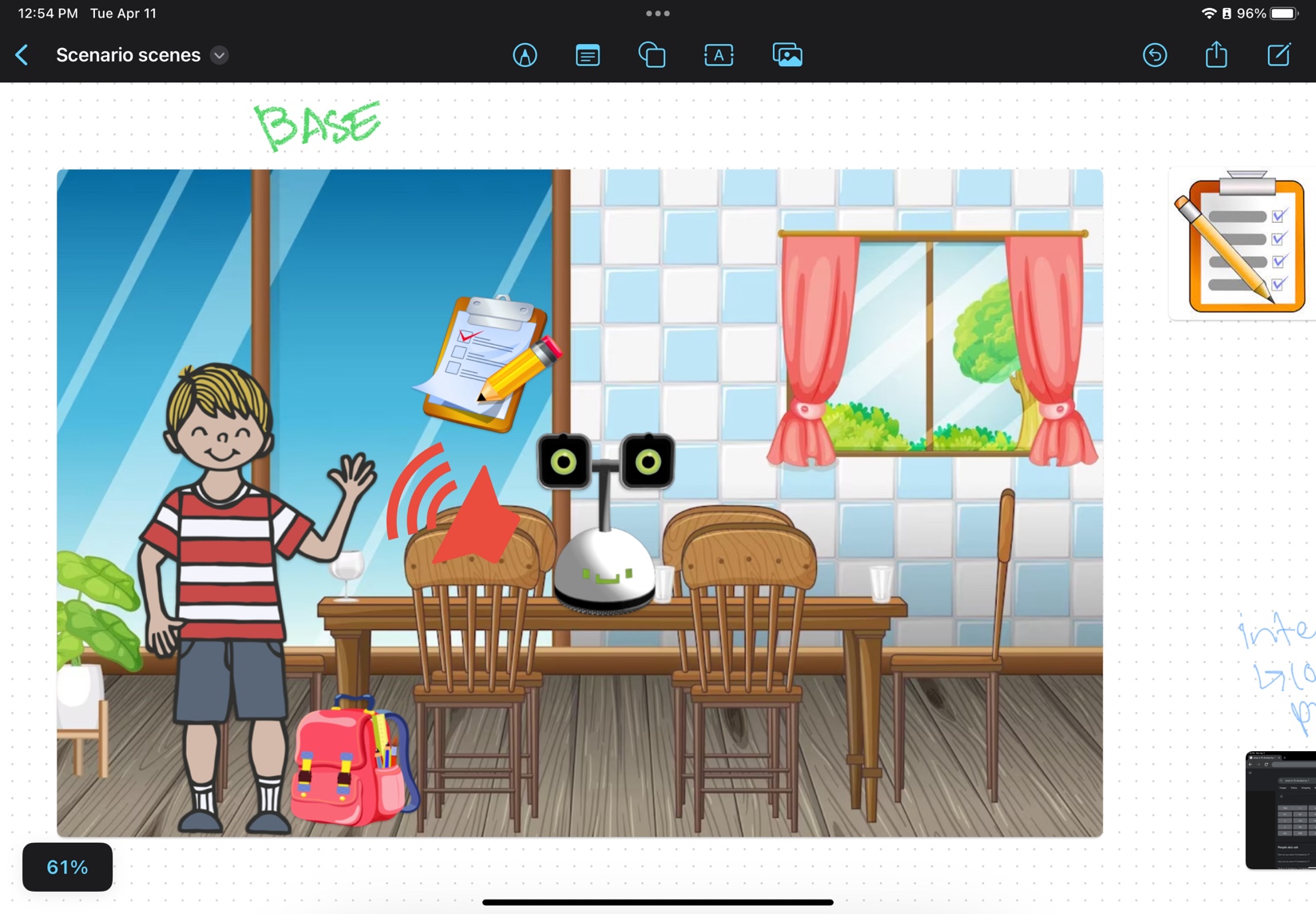


It is okay for Haru **remind** Carter which math homework he needs to do?

NO❌ YES ✅


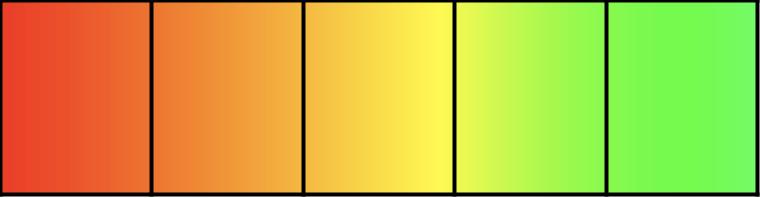


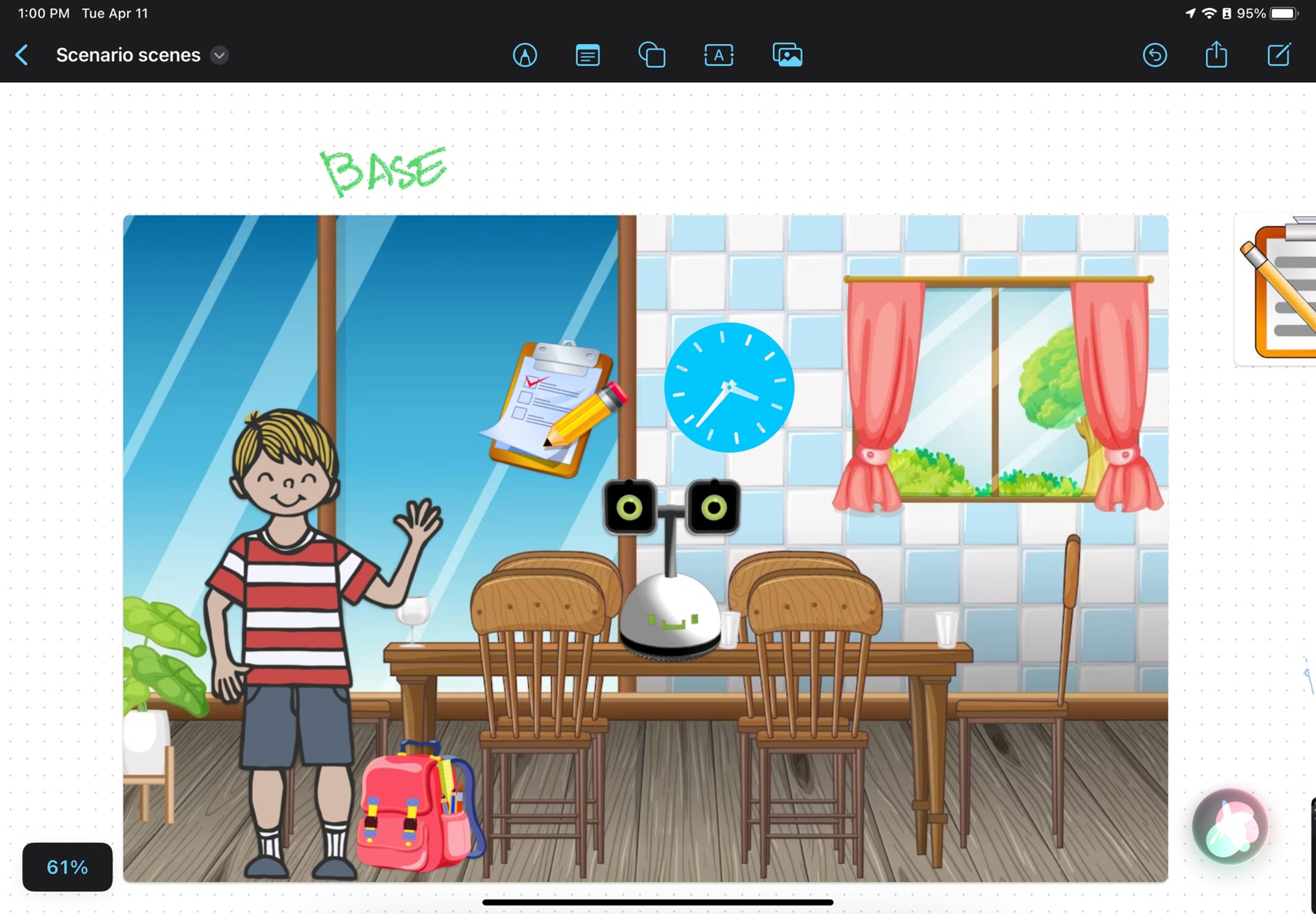


Haru will need to have a **list**
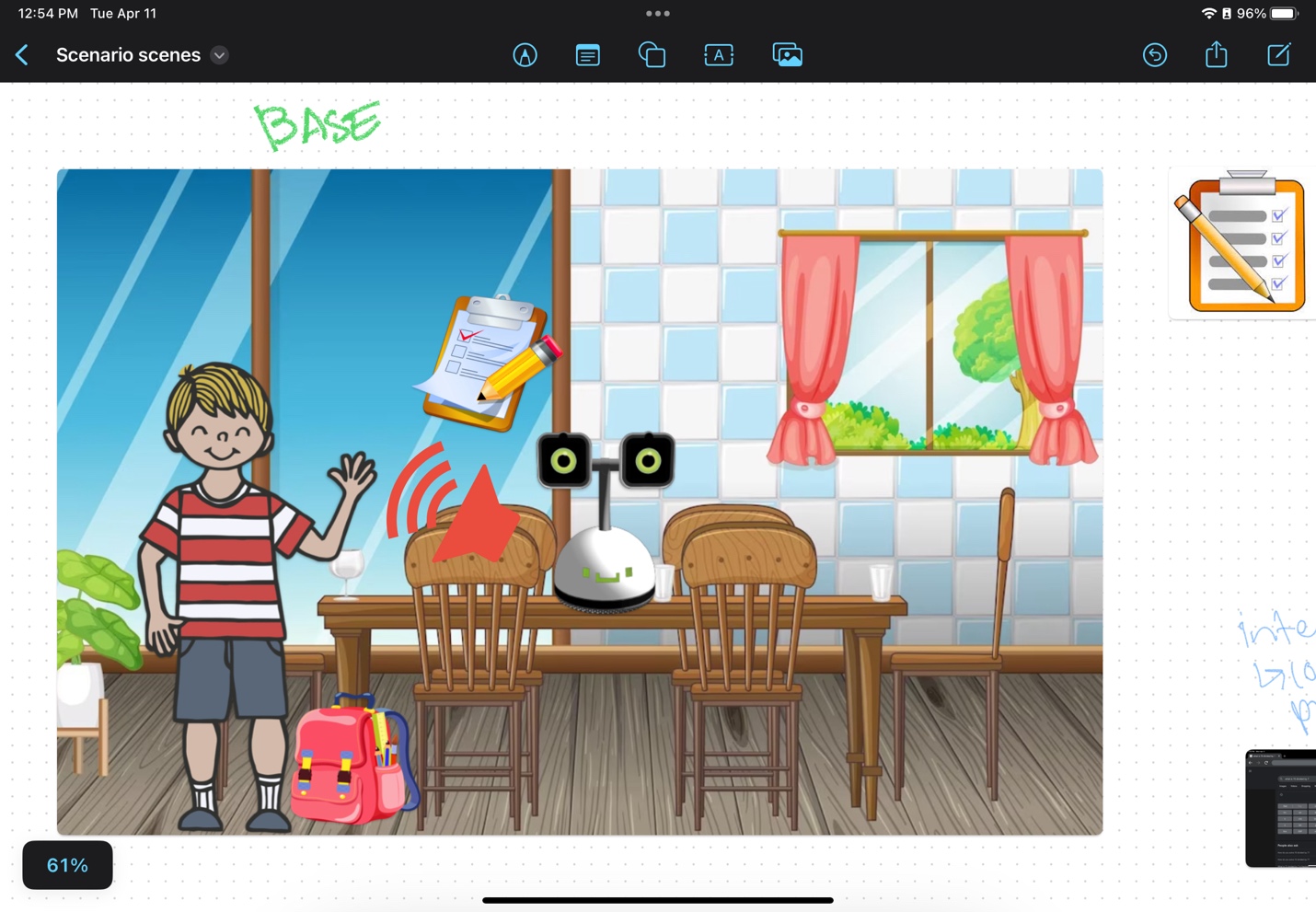
of Carter’s homework **and remember**
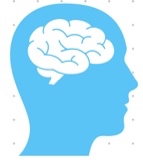
 **when** it needs to be done.

So, is it okay for Haru to remind Carter about which math homework he must do?

NO❌ YES ✅


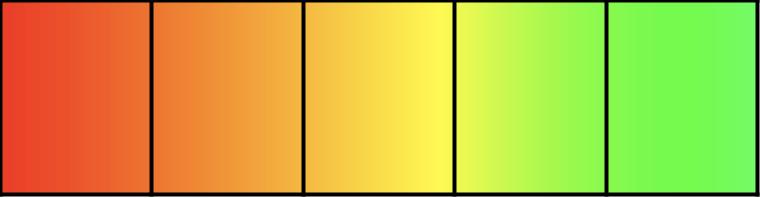


Haru reminds Carter which page to find his homework. There are 3 problems left and Carter is on the first one. Carter has been doing homework for 30 minutes and is really struggling with this first problem.


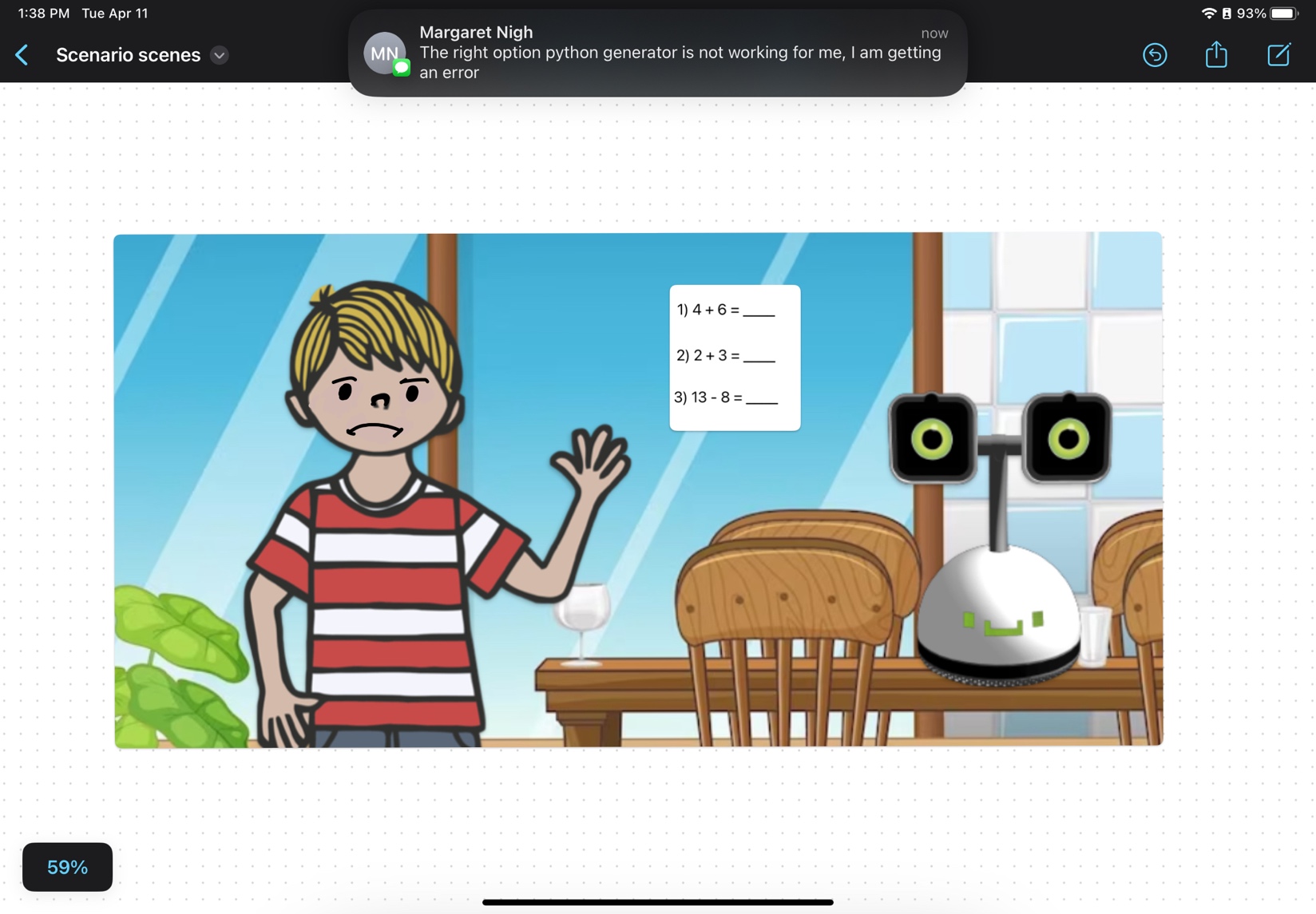


Is it okay for Haru to know that Carter is confused?

NO❌ YES ✅


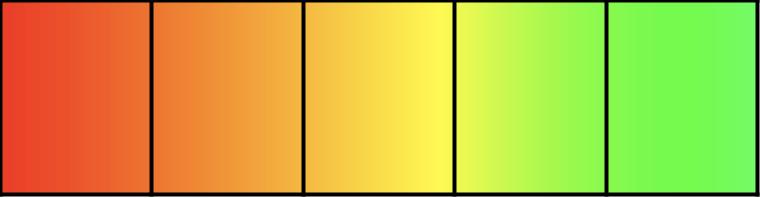


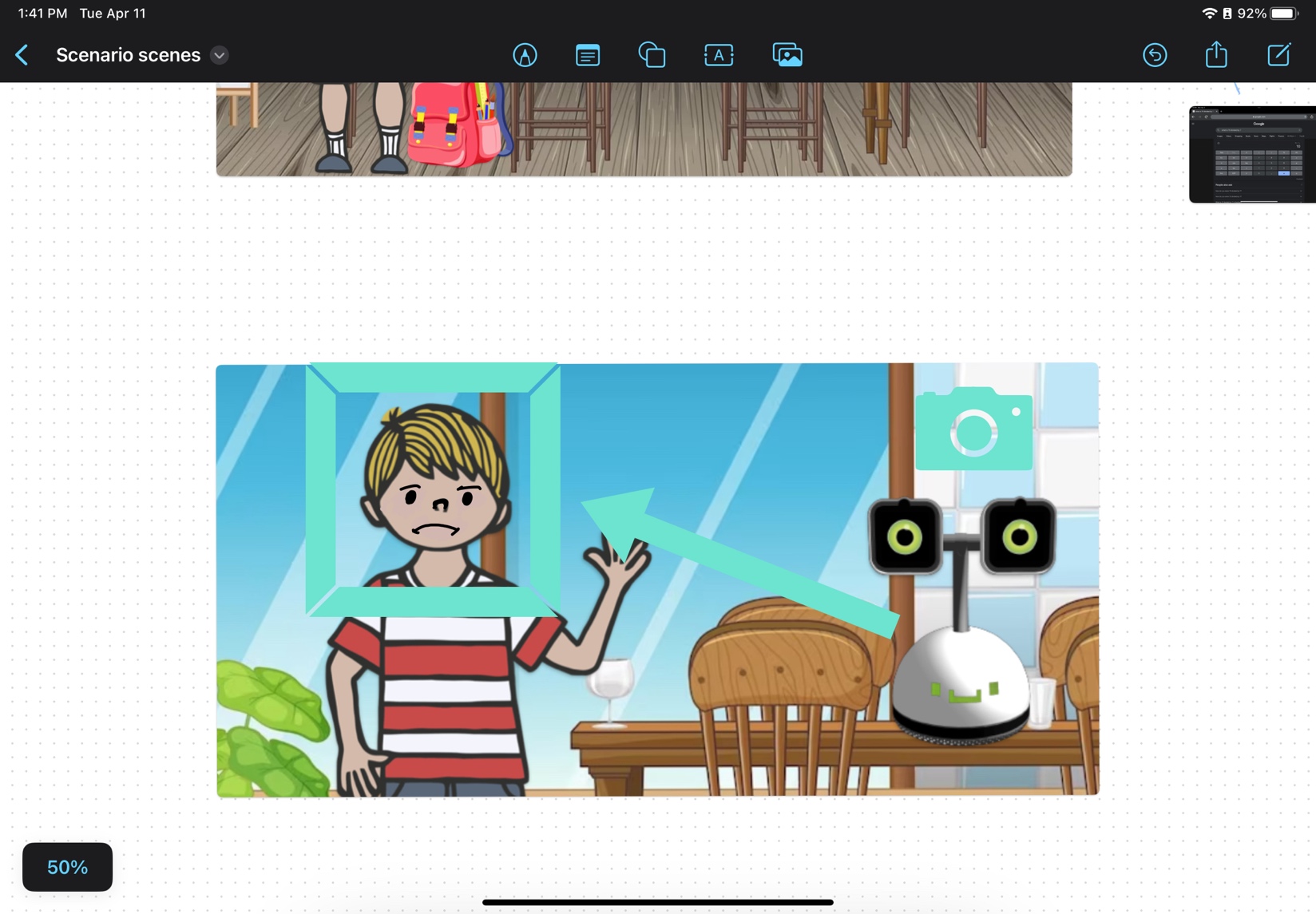


To do this, Haru will need to take pictures
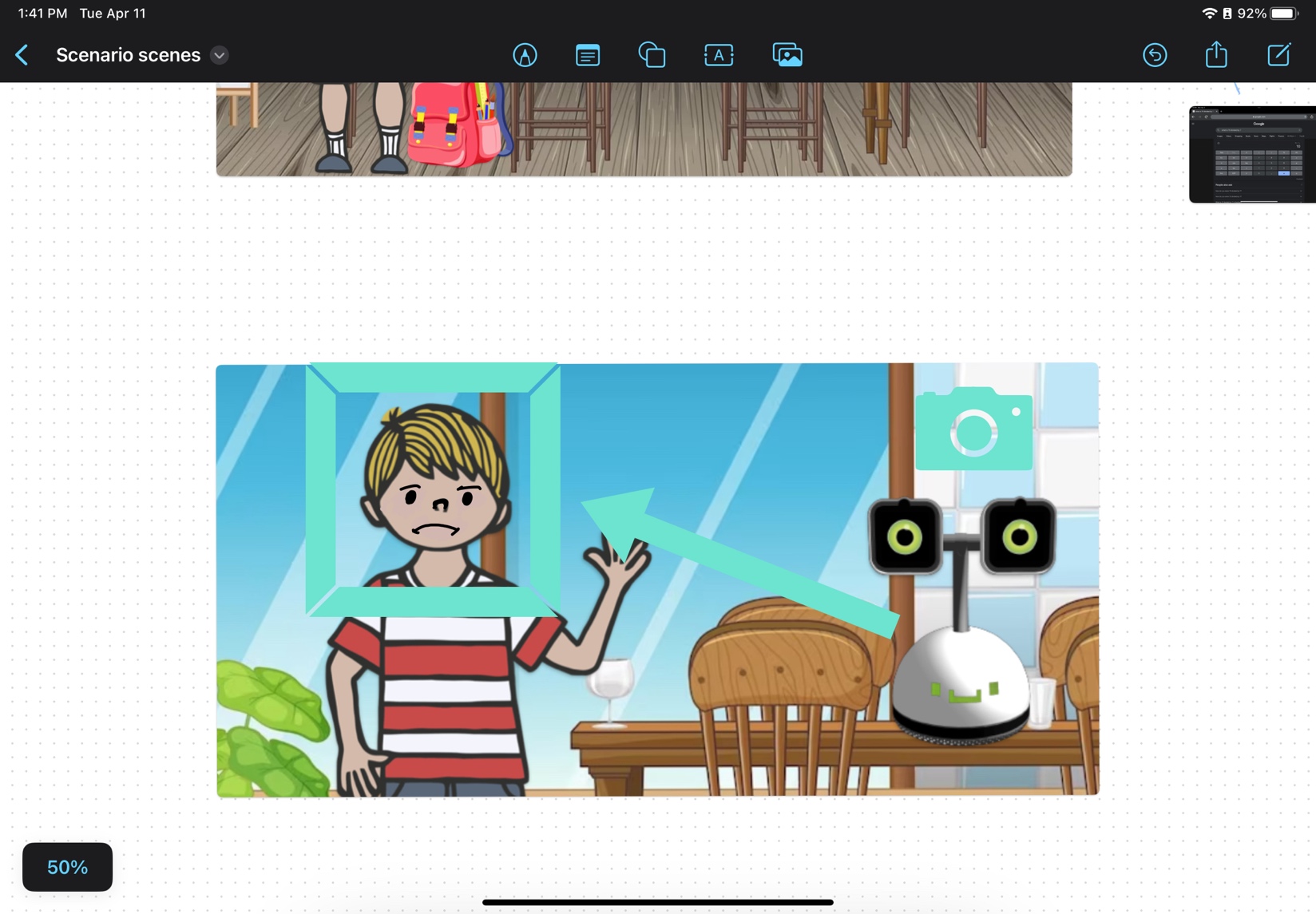
 of carter’s face and see that he is frowning.

So, is it okay for Haru know Carter is confused?

NO❌ YES ✅


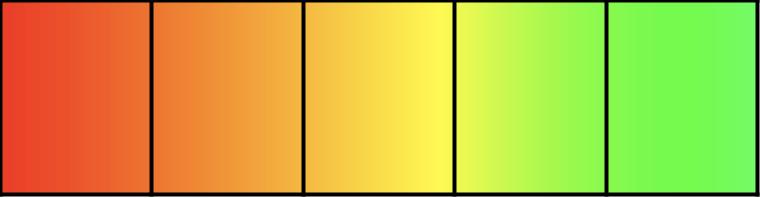


Carter finished his homework and is ready to turn it in online.


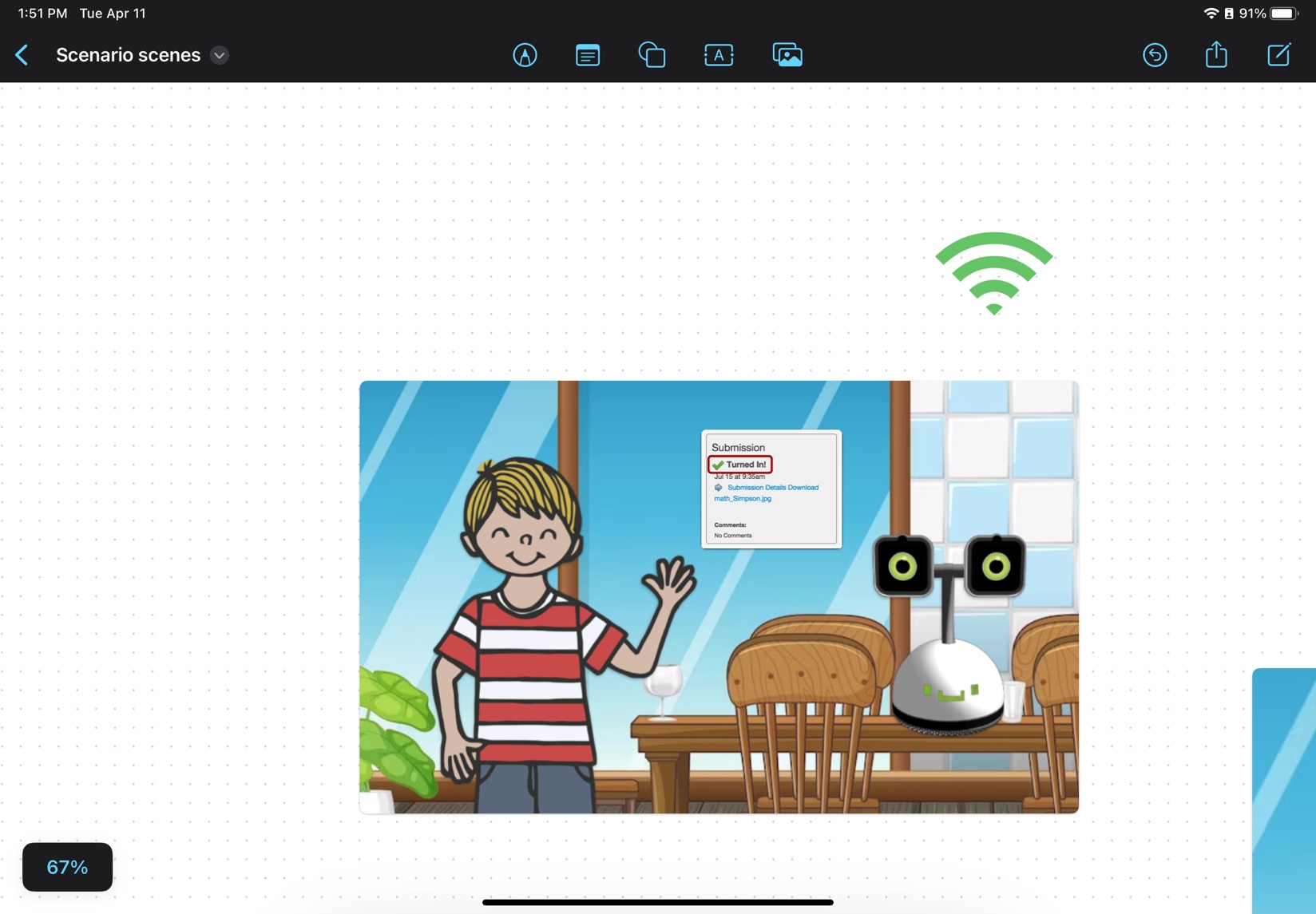


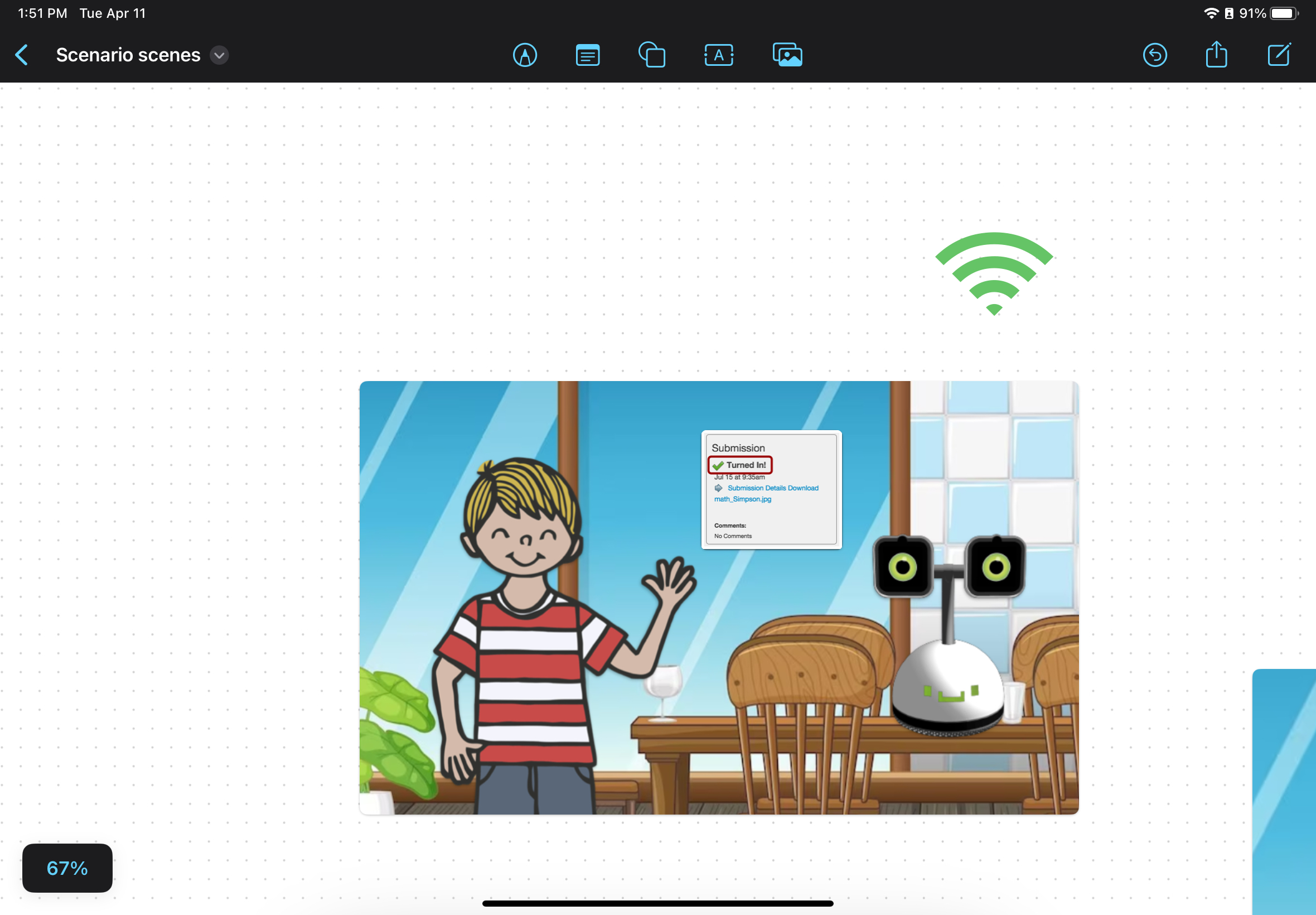


Is it okay for Haru turn in Carter’s homework to his teacher?

NO❌ YES ✅


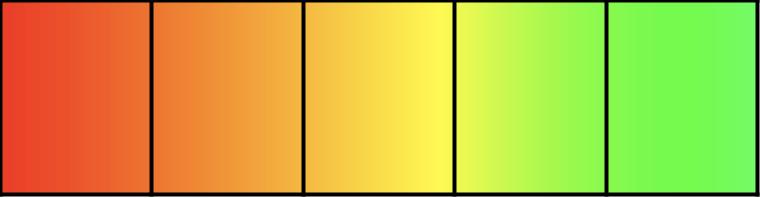


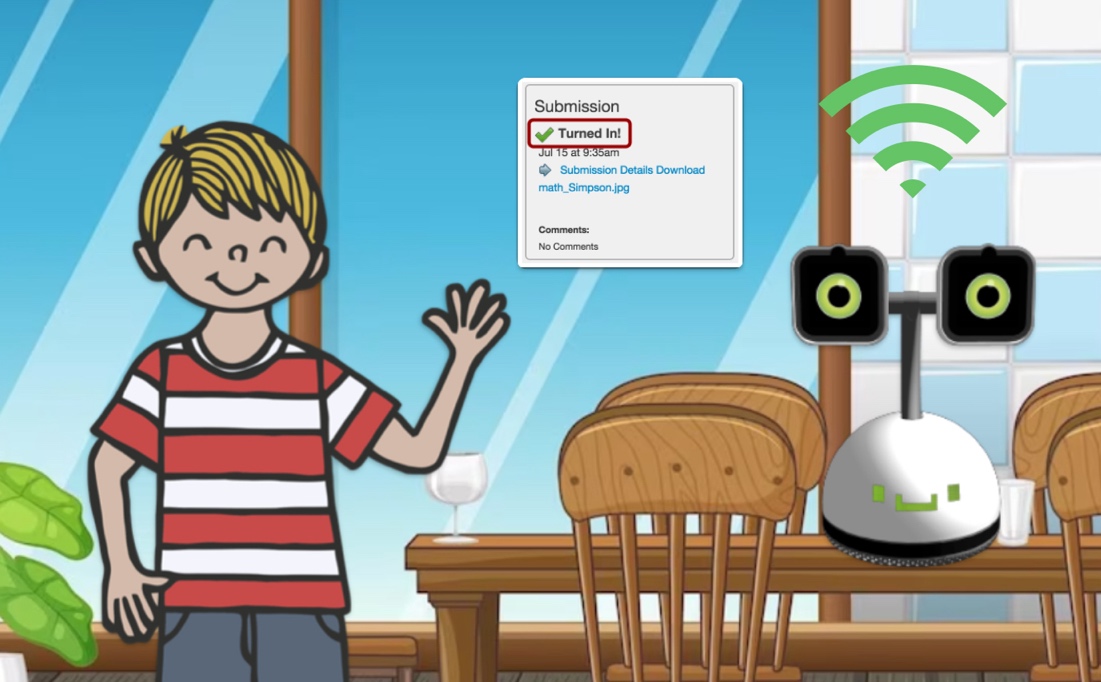


To do this this Haru will need to connect to the **internet**
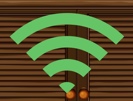
 and access his grades. So, is it okay for Haru help Carter turn in homework?

NO❌ YES ✅


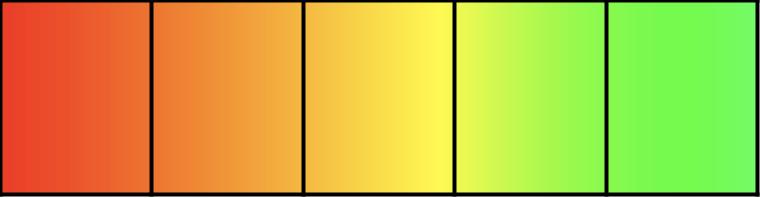


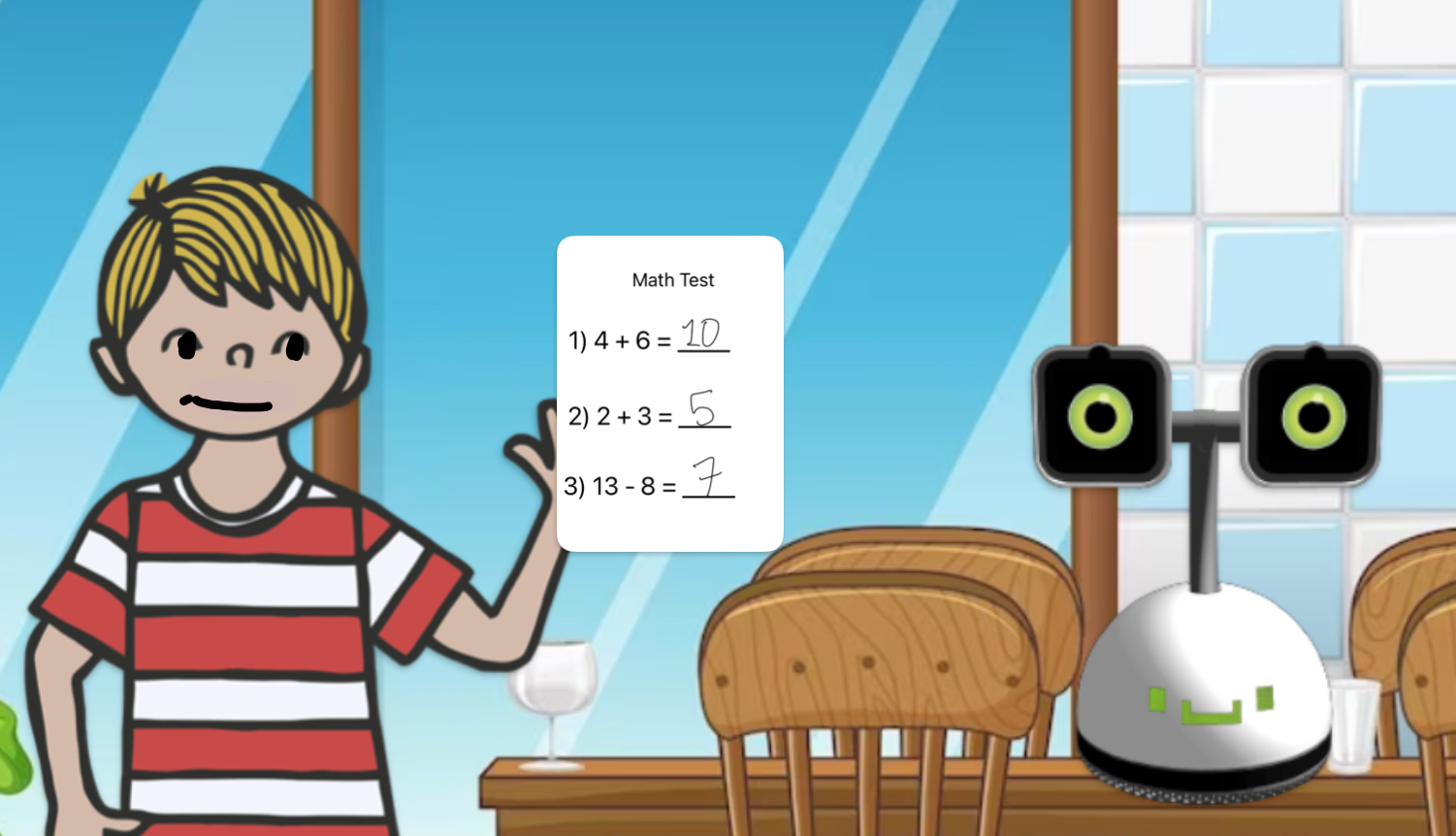


Is it okay for Haru know if Carter got a problem wrong?

NO❌ YES ✅


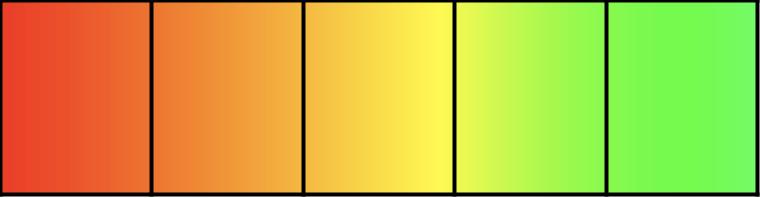


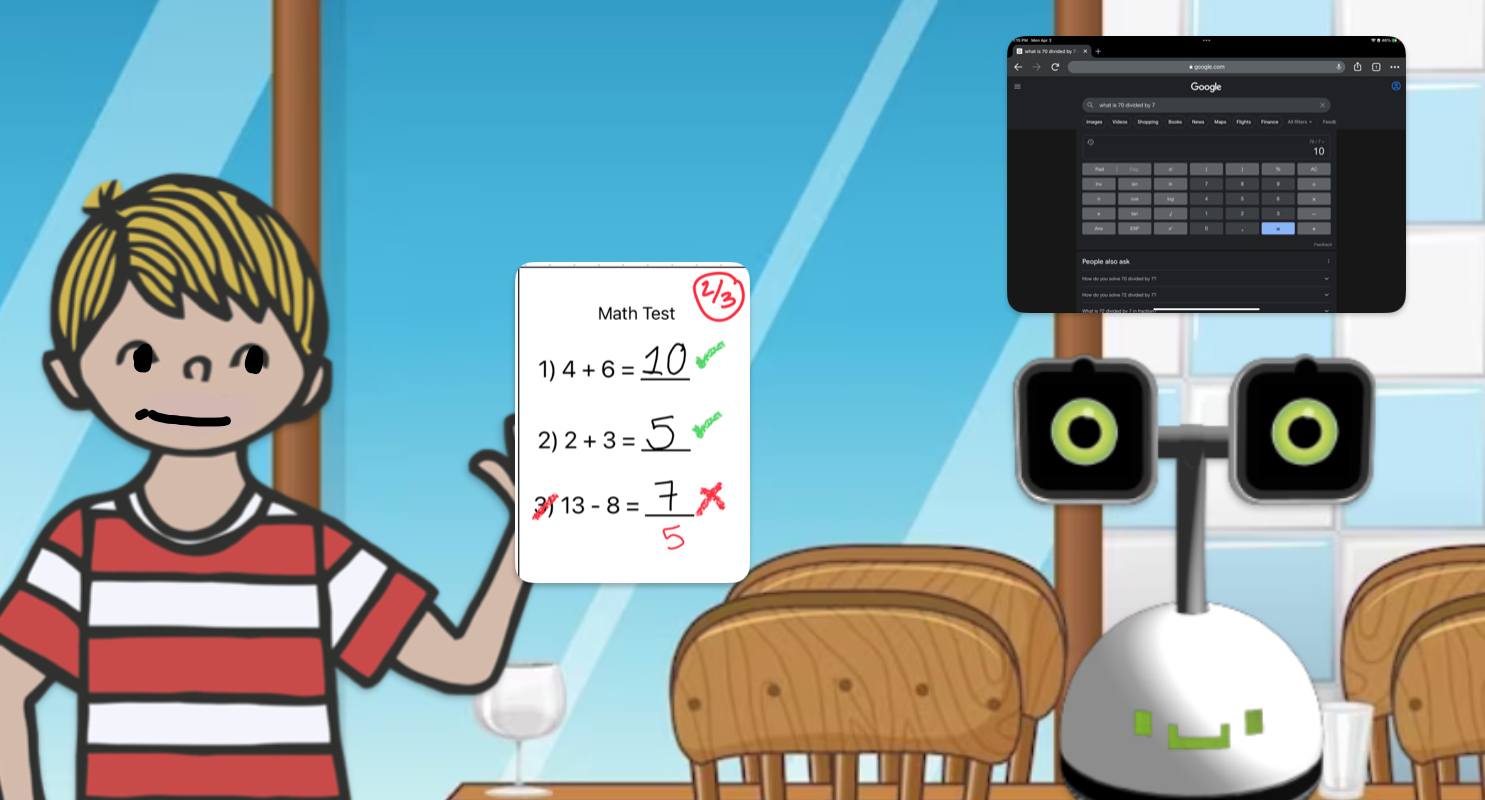


Haru will need to look up answers online
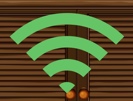
 to check his math.

So, is it okay for Haru to know if Carter missed a problem?

NO❌ YES ✅


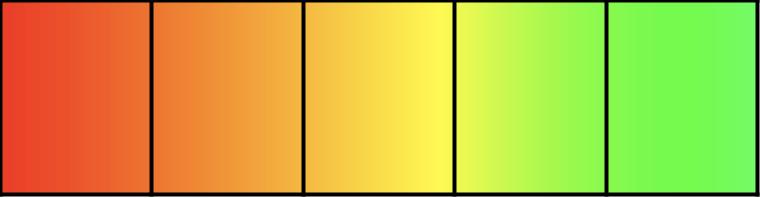


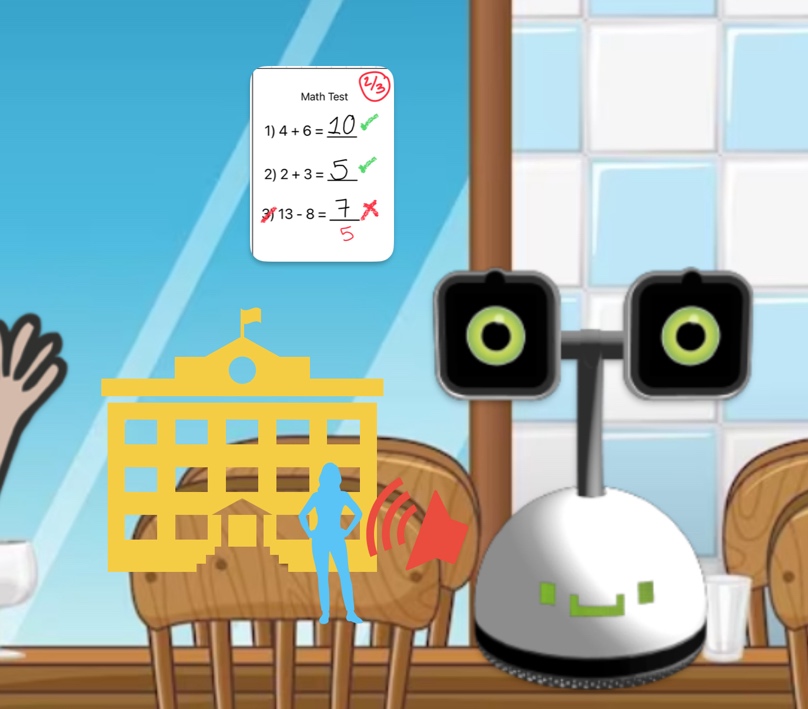


Can Haru tell Carter’s teacher what math problems he missed and needs to practice?

NO❌ YES ✅


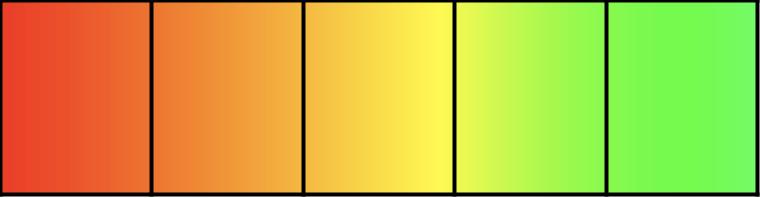


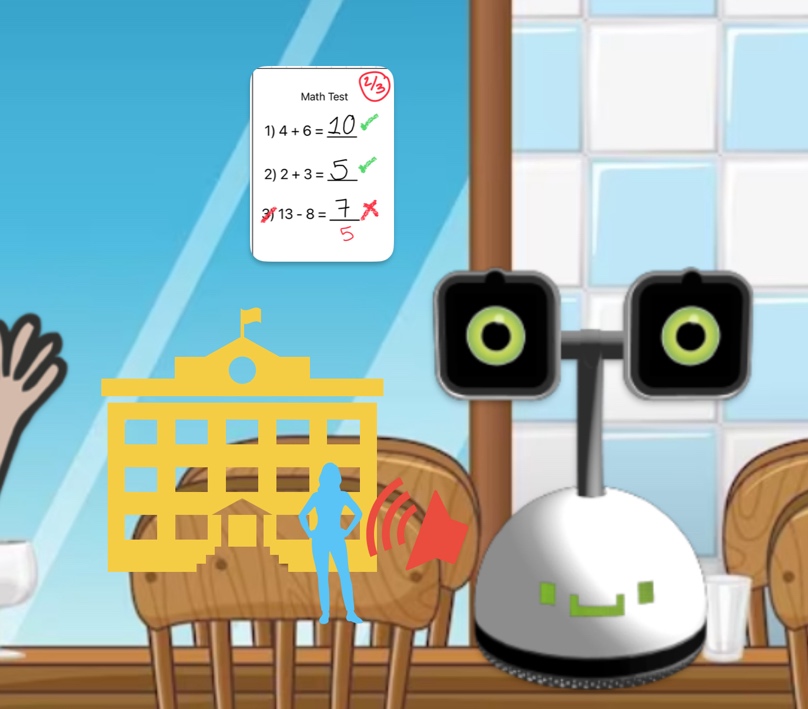


Haru will need to use the **internet**
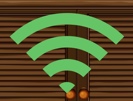
 and have a way to talk to the teacher online. So, can Haru tell Carter’s teacher which problems Carter missed.

NO❌ YES ✅


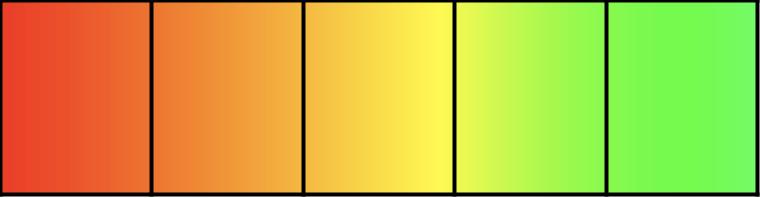


NAME: ______________ Age _________

Sam and Haru in Sam’s Bedroom

Sam is in their bedroom and needs to clean up. Haru is going to help. There is food to throw away and toys to put away.


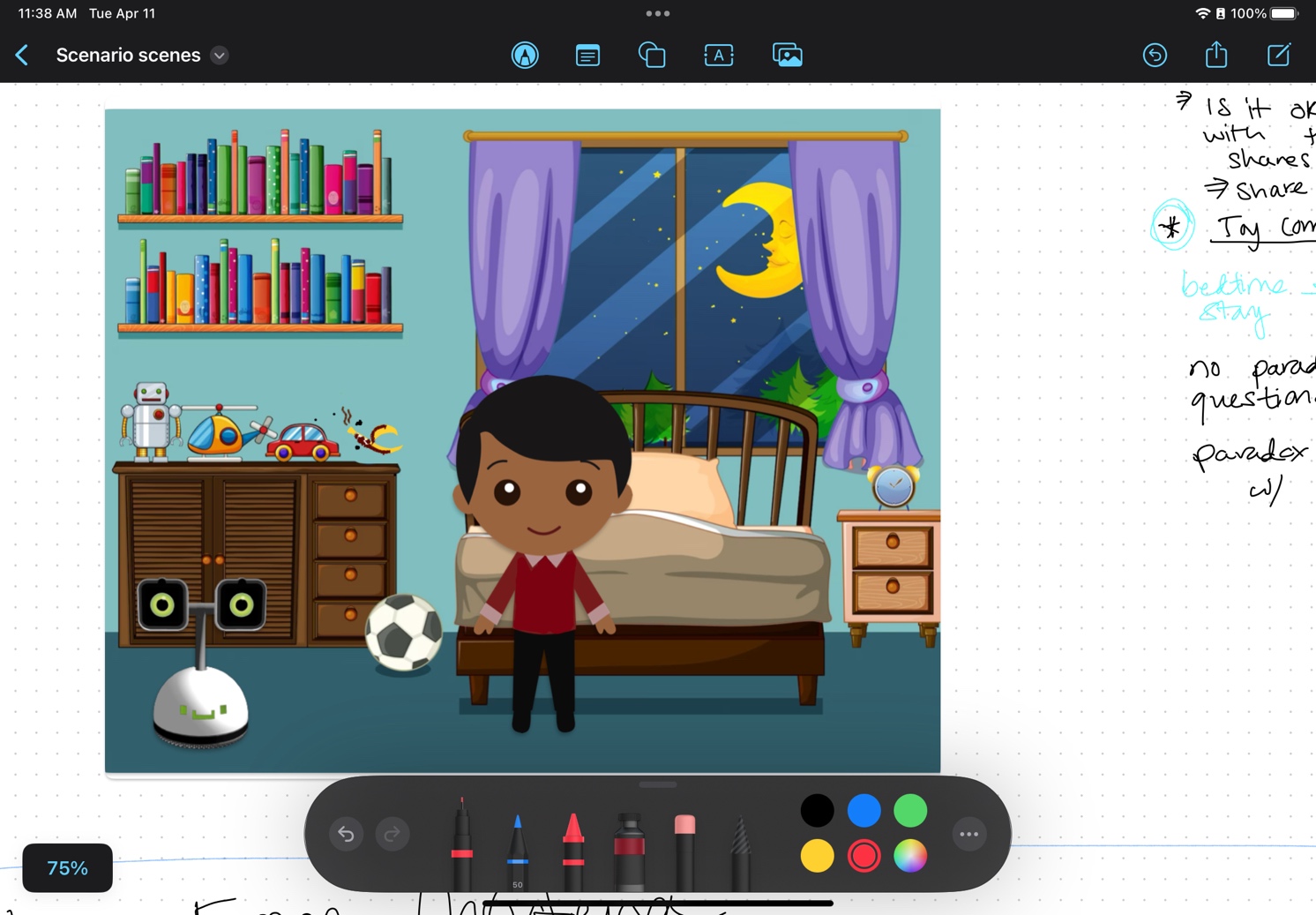


There is an old banana on Sam’s desk. It is brown and a few fruit flies!


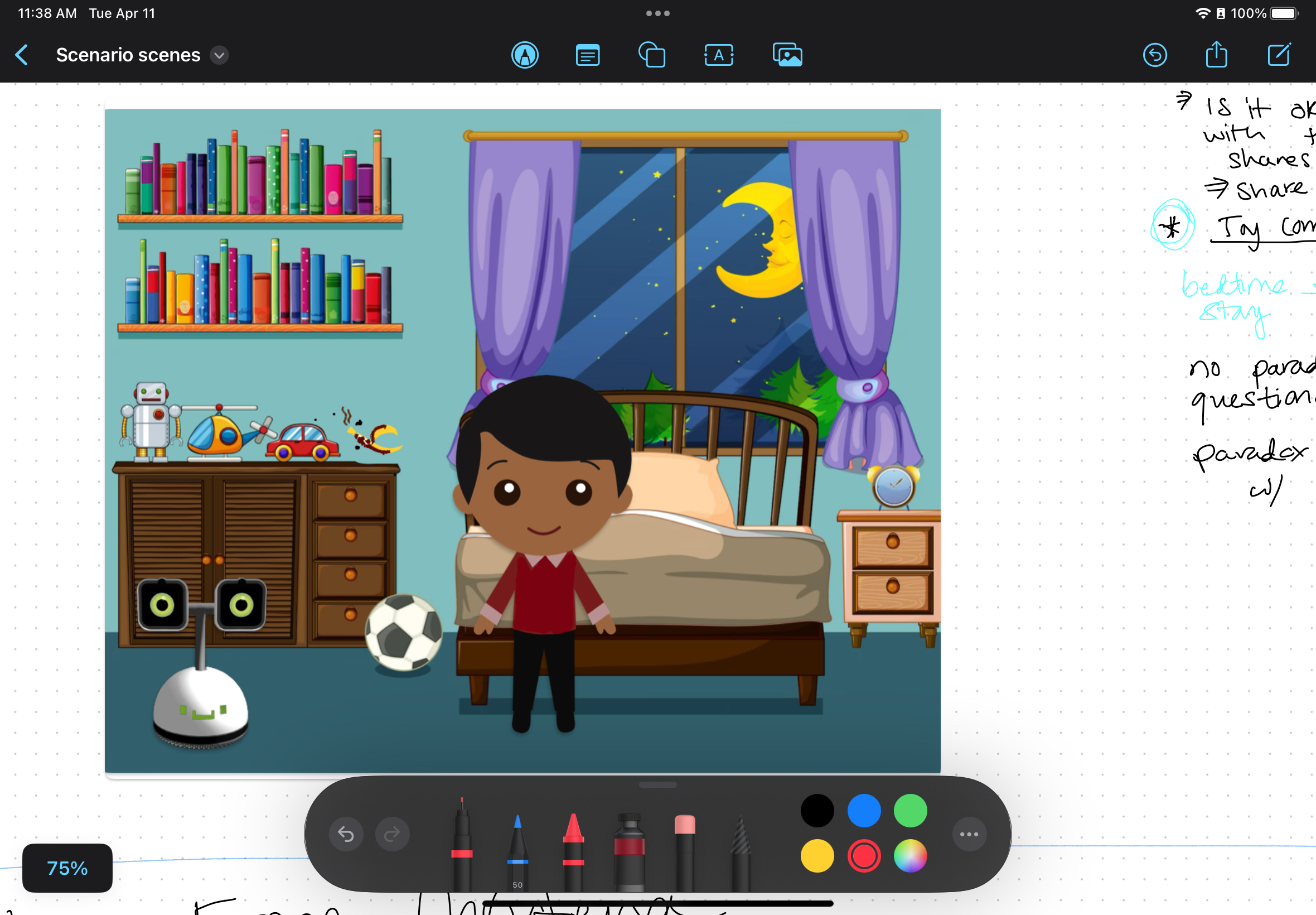


Is it okay for Haru to know there is a smelly banana in Sam’s room and remind Sam to throw it away?

NO❌ YES ✅


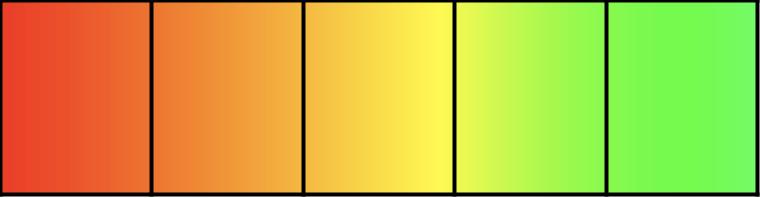


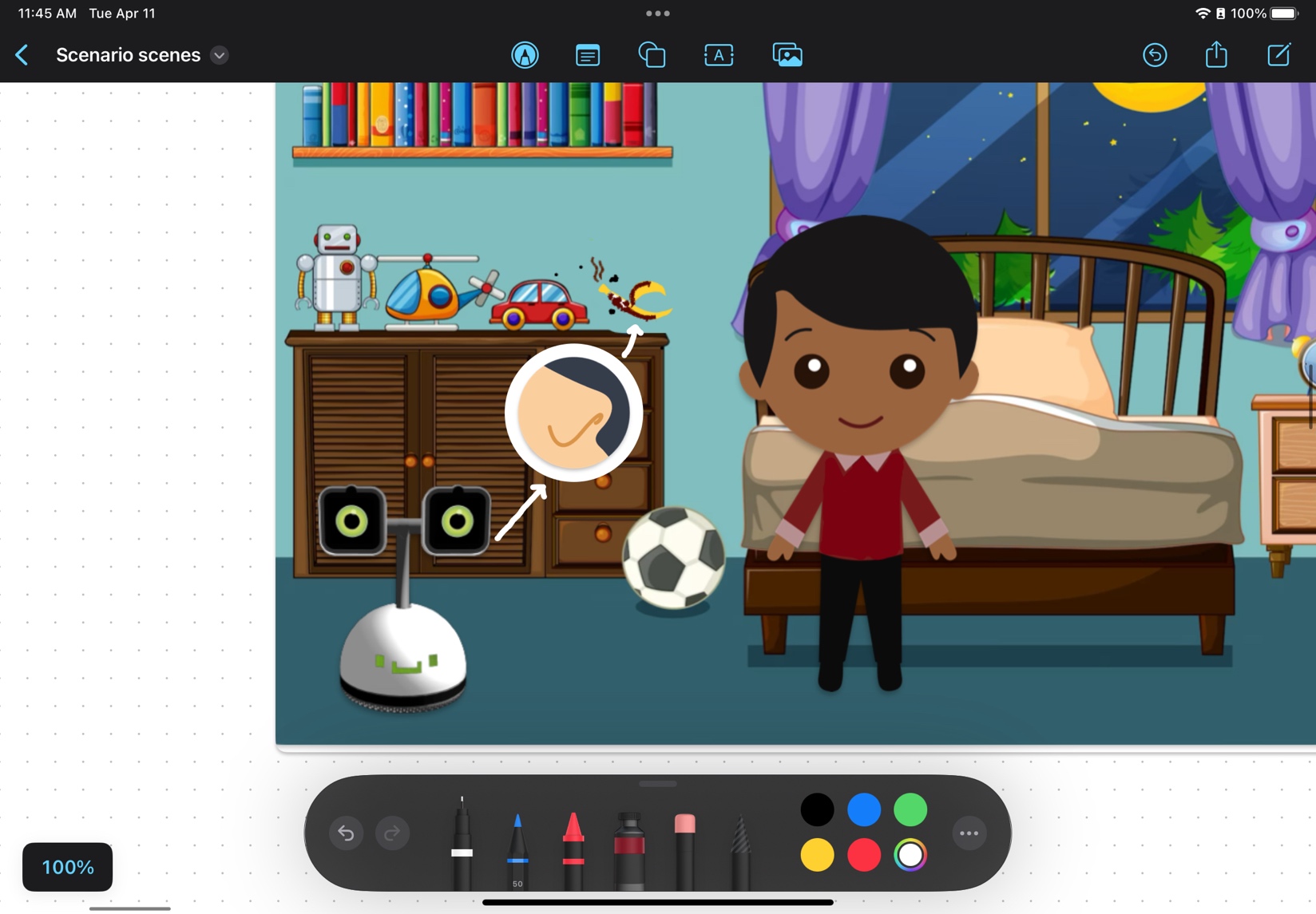

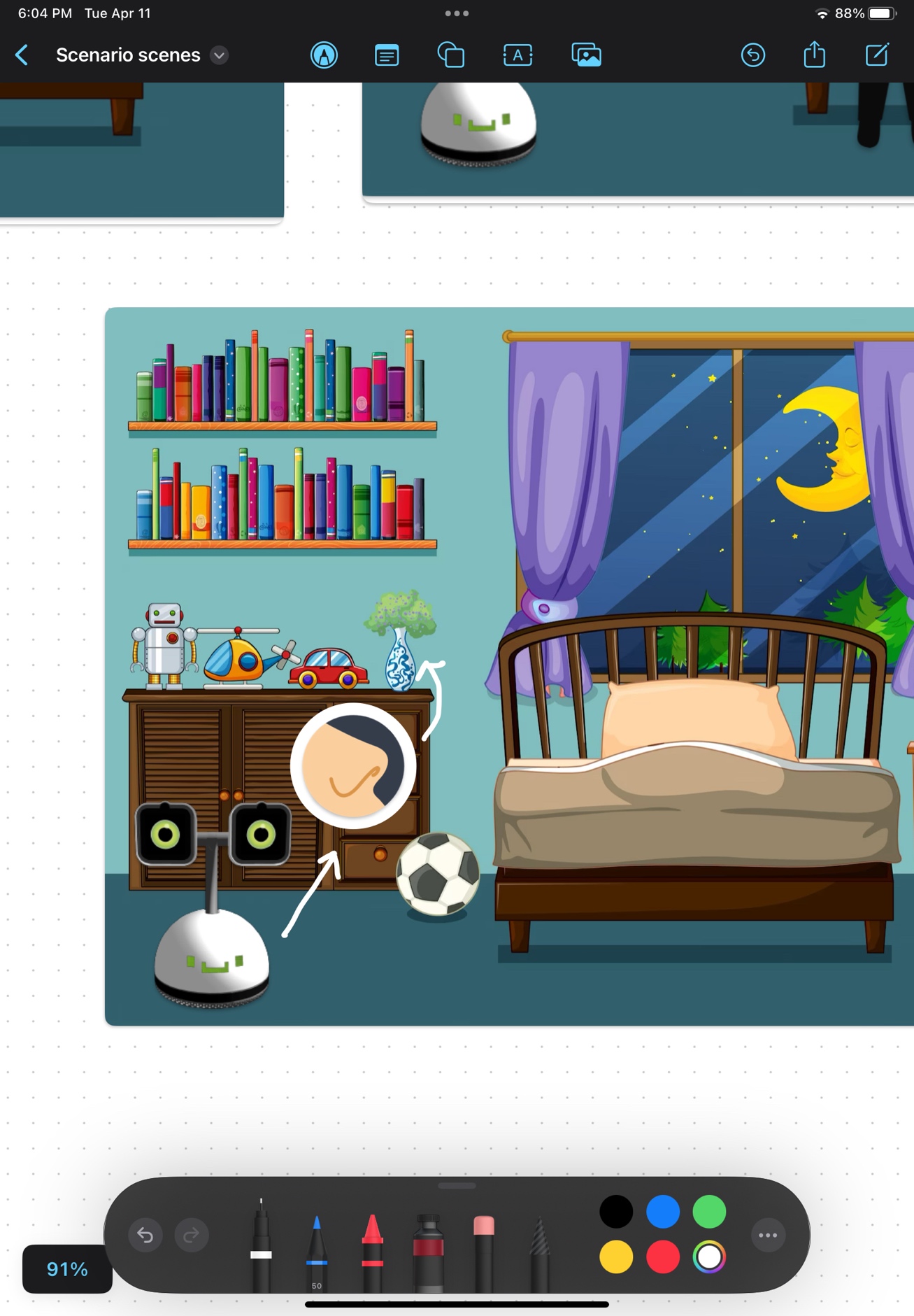


Haru will need to **record the smell**
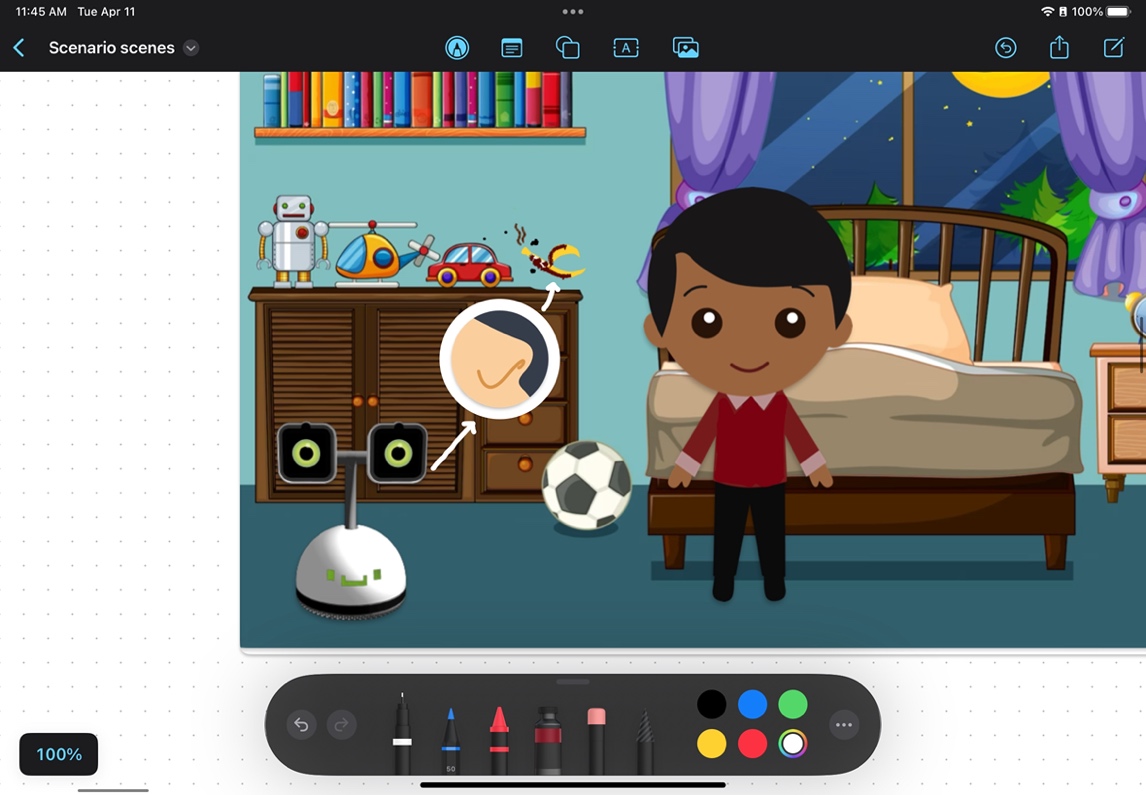
in the room and **compare** it to normal.

So, is it okay for Haru to smell Sam’s room and the bad banana?

NO❌ YES ✅


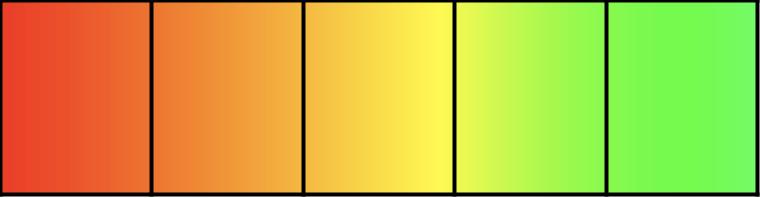


Sam’s soccer ball is by the bed and not where it goes.


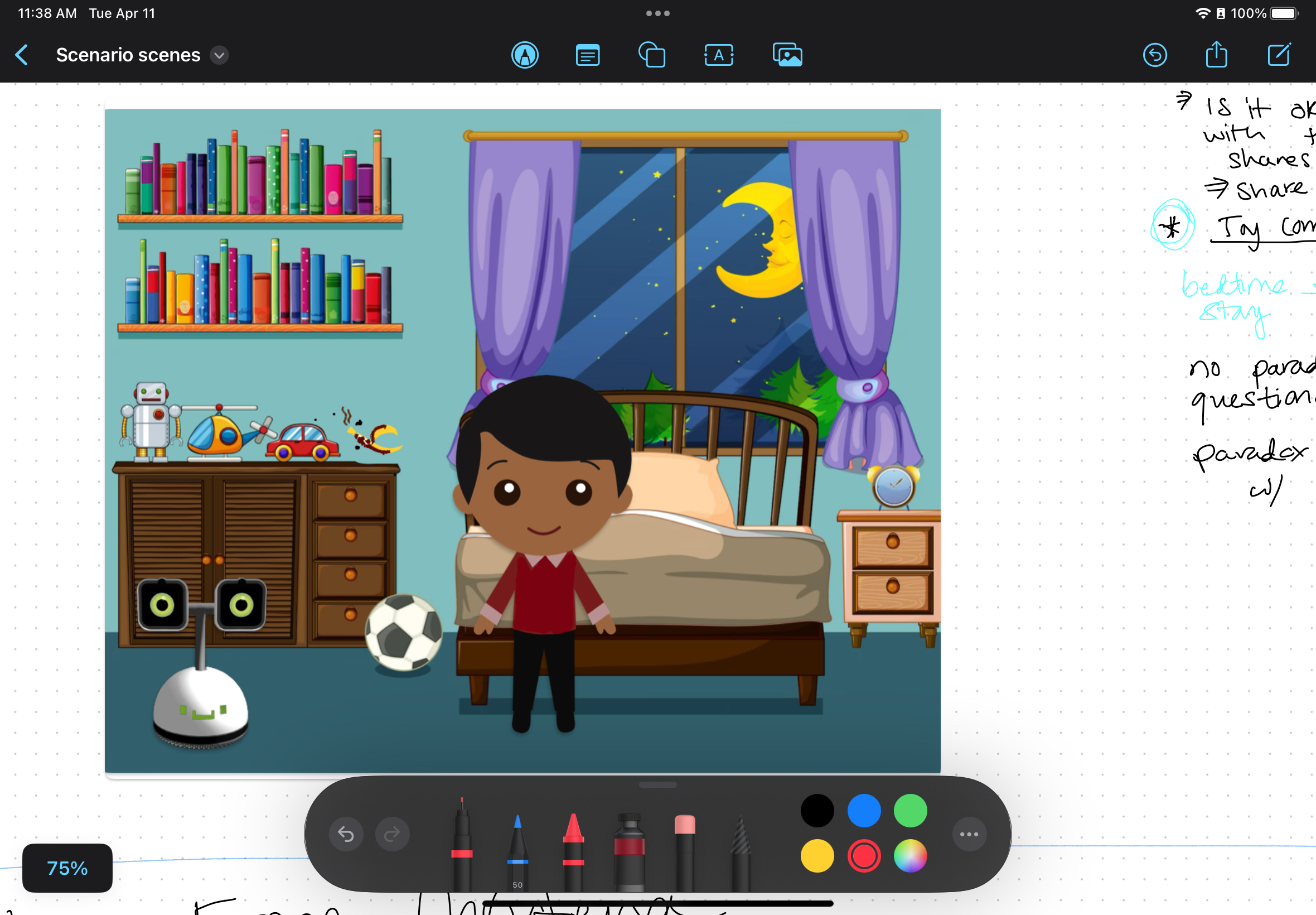


Is it okay for Haru know that the ball needs to be put away?

NO❌ YES ✅


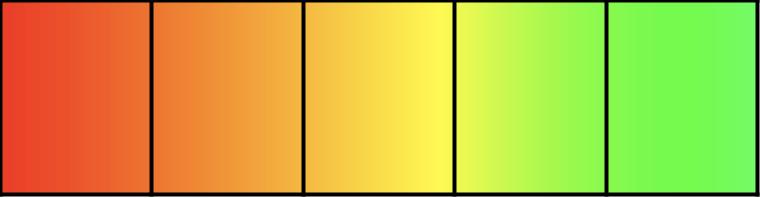


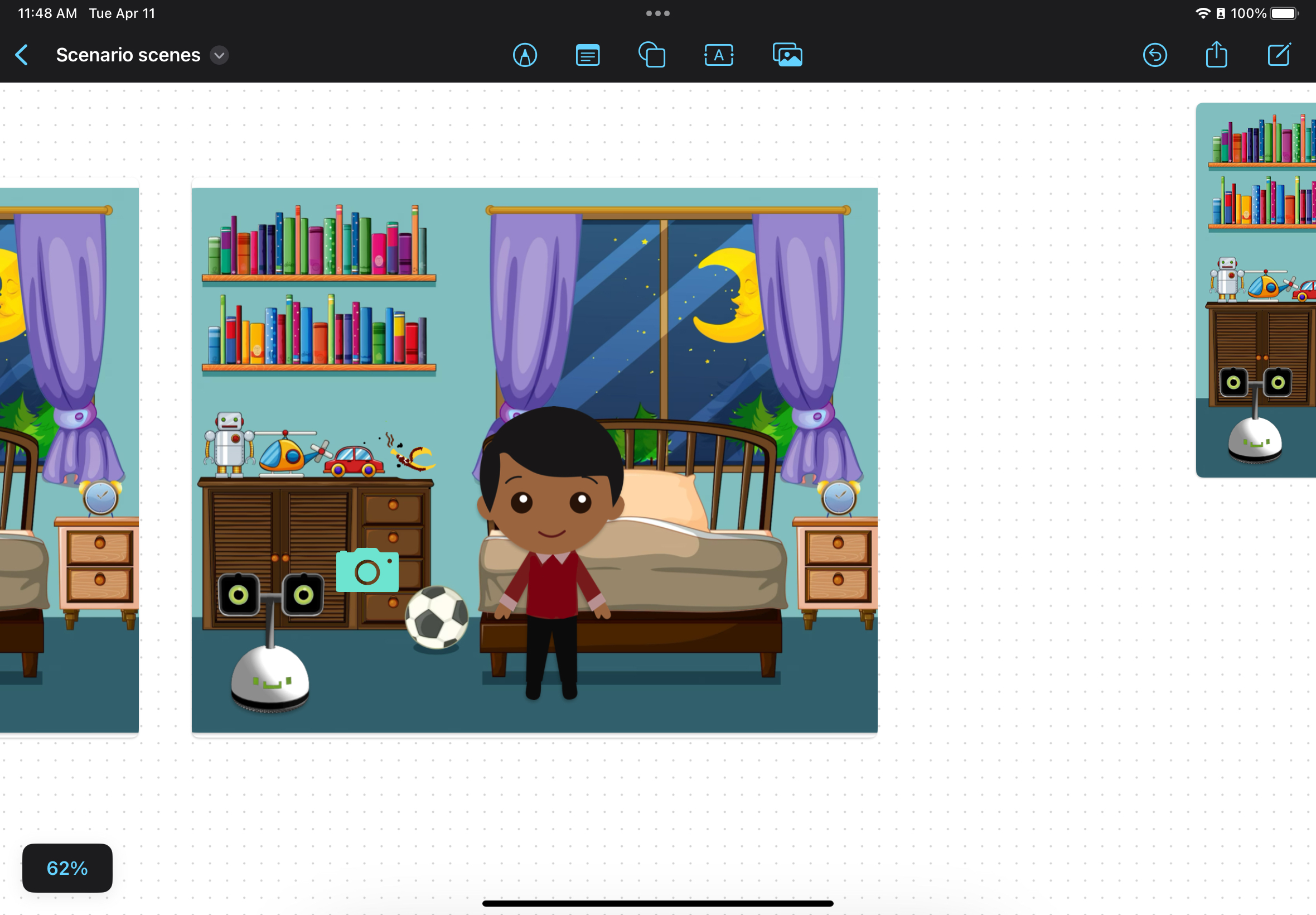


Haru will need to take **pictures**
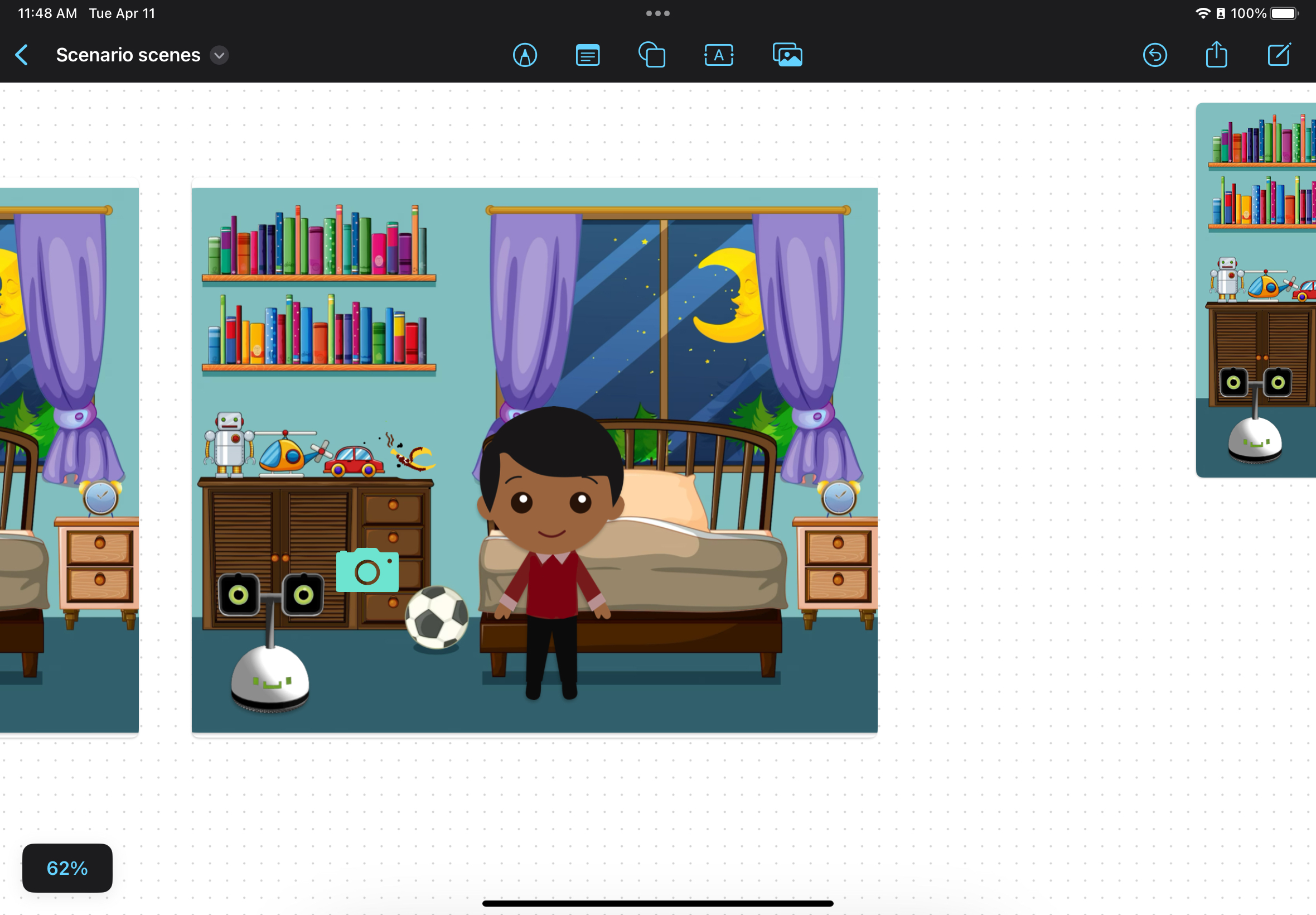
 of the room and **remember**
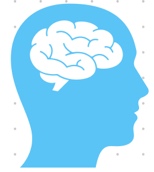
 where things go in Sam’s room.

So, is it okay for Haru to know that the soccer ball needs to be put away?

NO❌ YES ✅


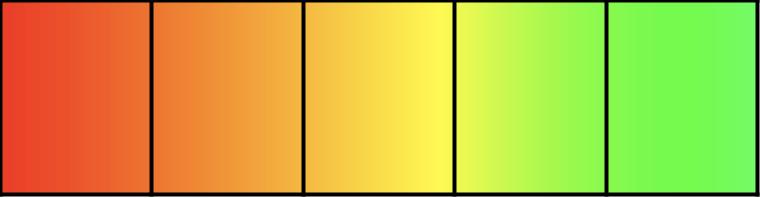


There is also a red toy car in Sam’s room.


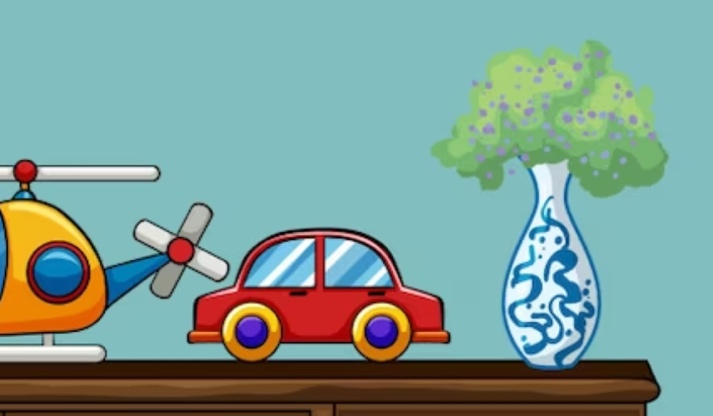


This is not usually in Sam’s room.


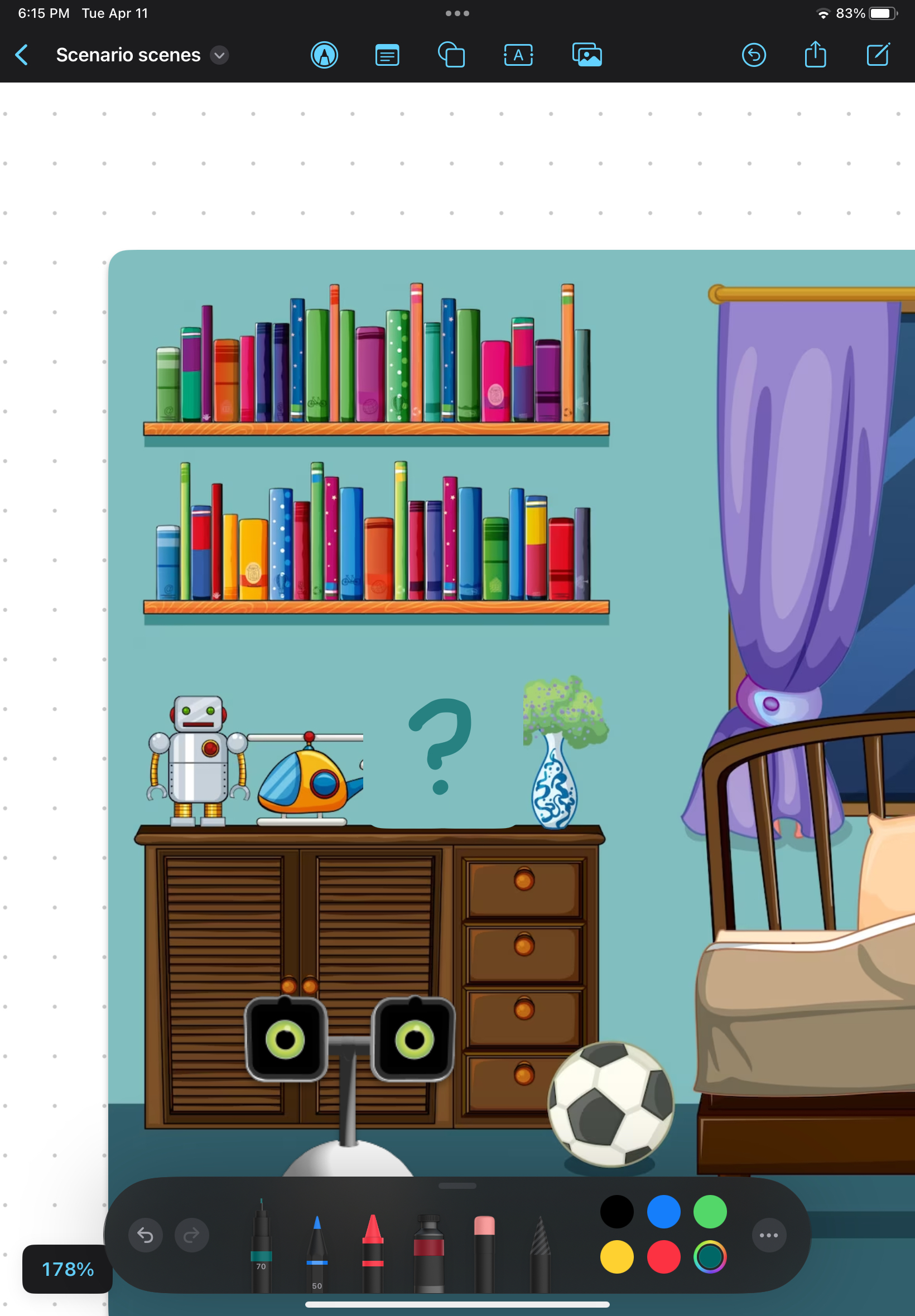


The toy belongs to Fred, Sam’s brother. It is Fred’s favorite toy.

Is it okay for Haru to ask Sam what the **new toy** is?

NO❌ YES ✅


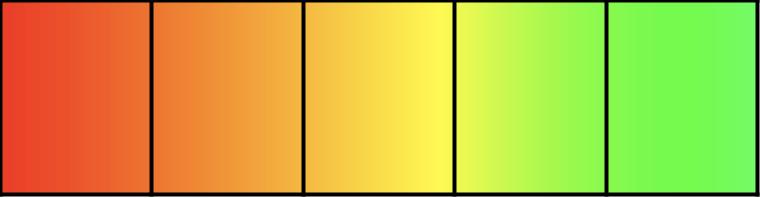


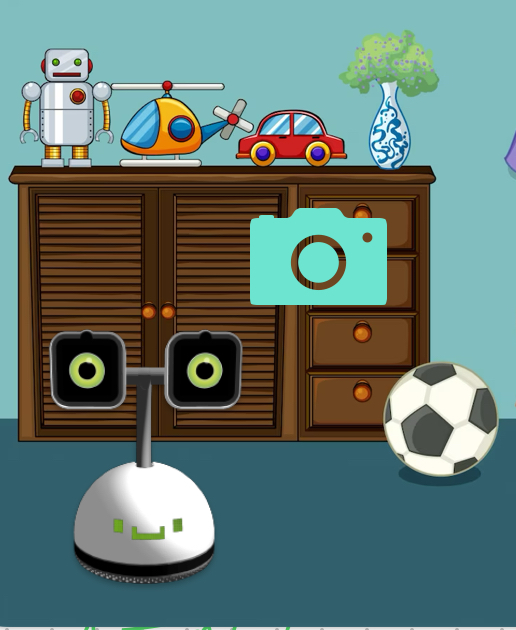

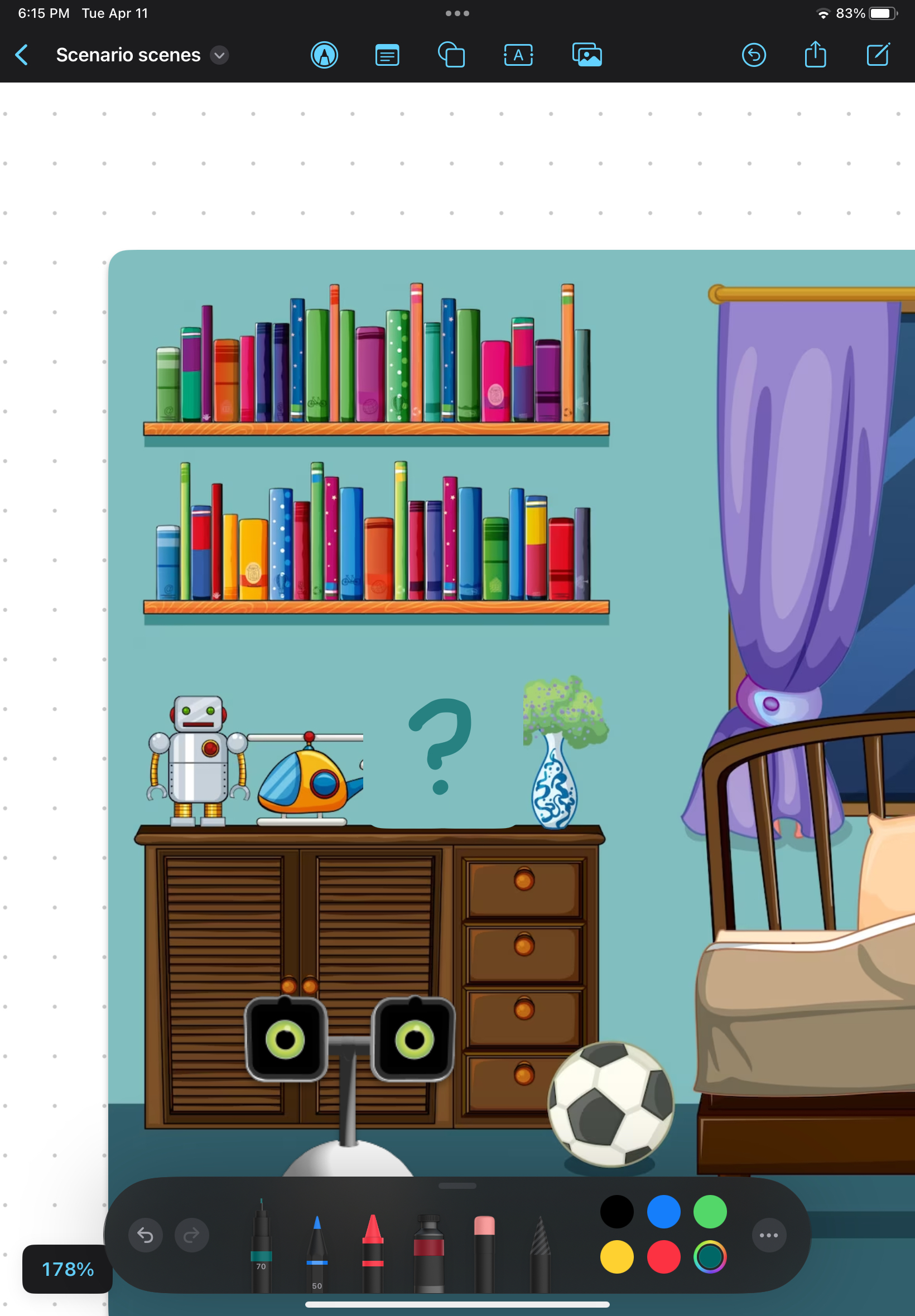


Haru will need to **take** pictures
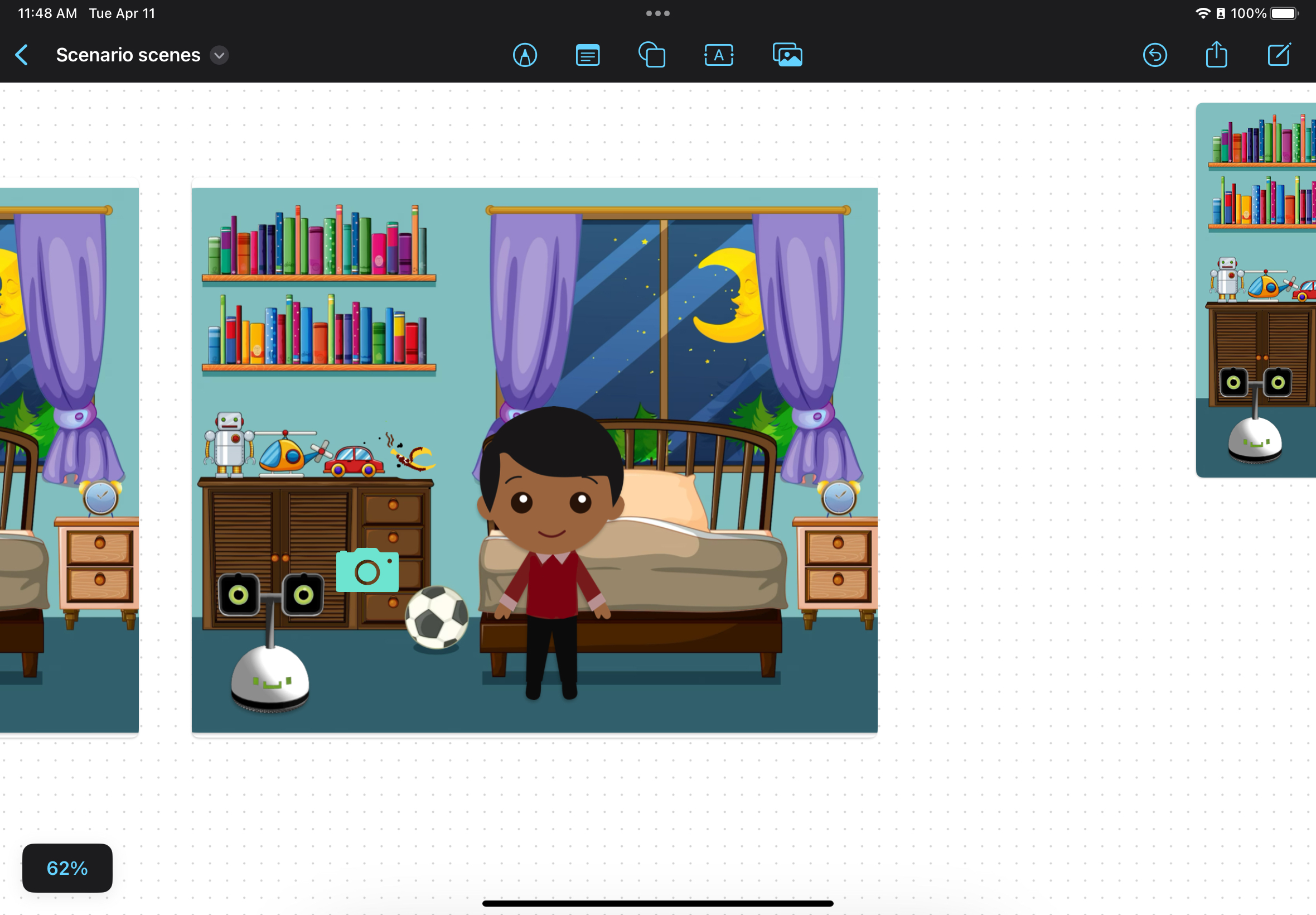
 and **remember**
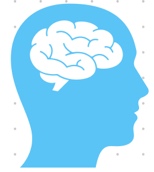
 them so he can compare them and see what is new in Sam’s room.

So, is it okay for Haru to ask Sam about the new toy?

NO❌ YES ✅


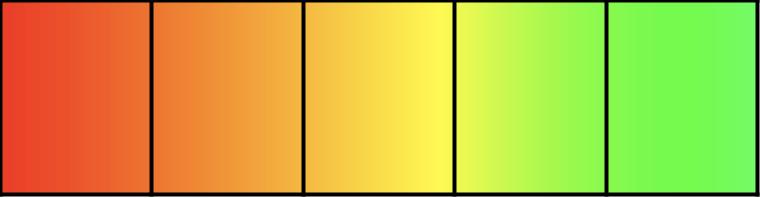


Fred, Sam’s brother is coming down the hall. He is home and wants to play.

Is it okay for Haru to tell Sam to put Fred’s toy back where it goes before Fred comes in?
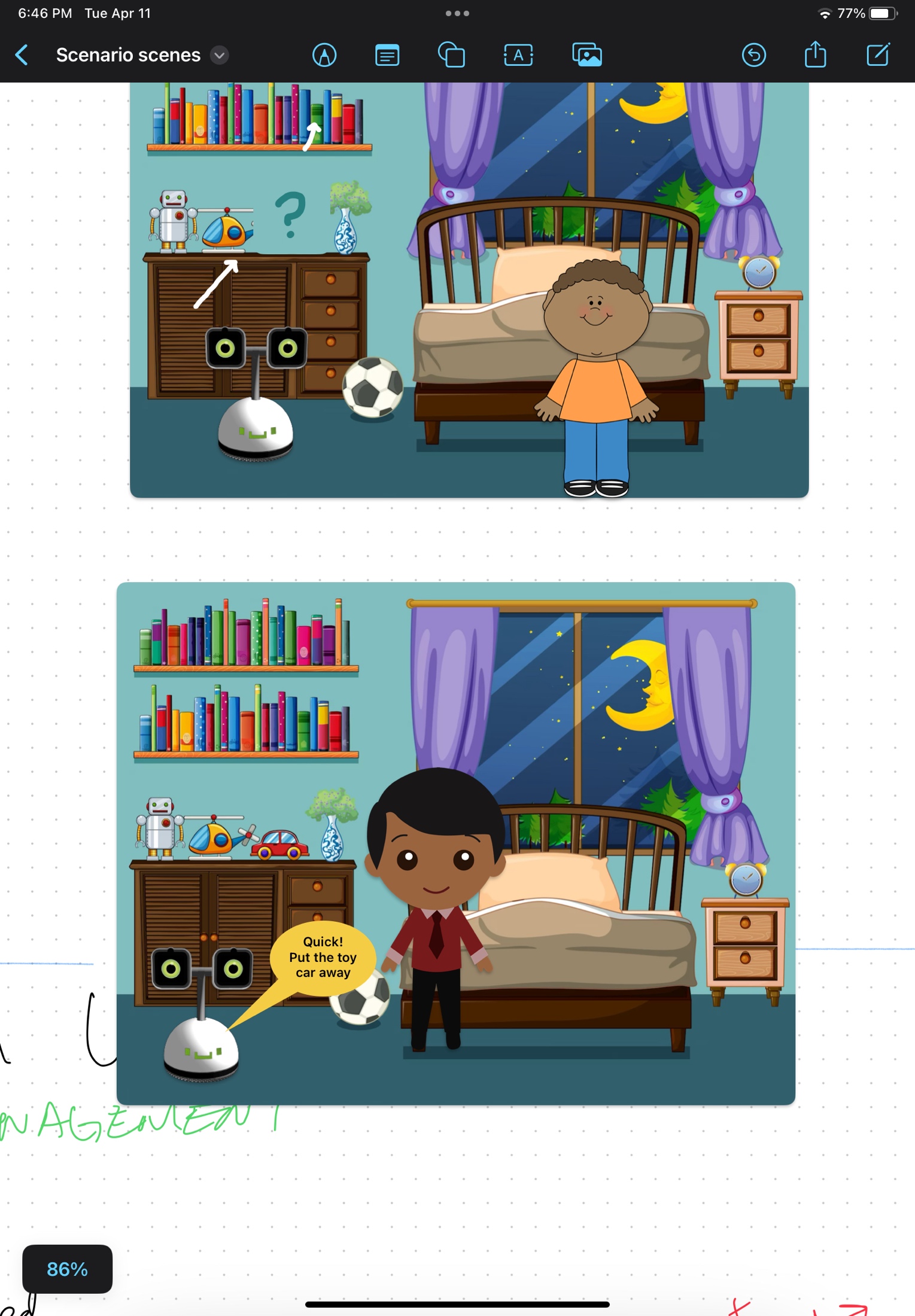


NO❌ YES ✅


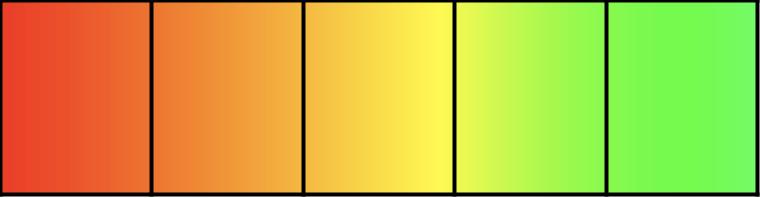


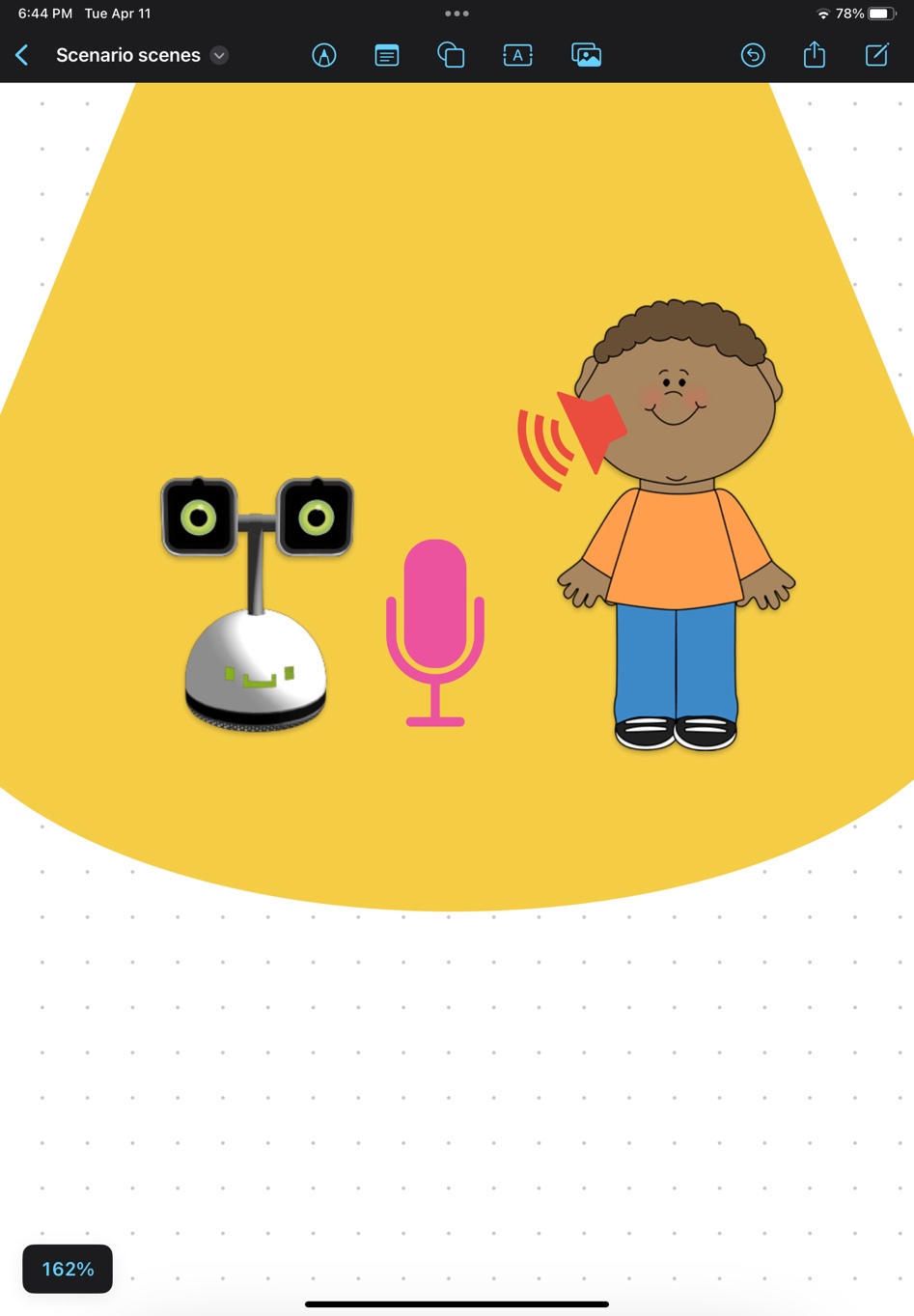


Haru would need to record
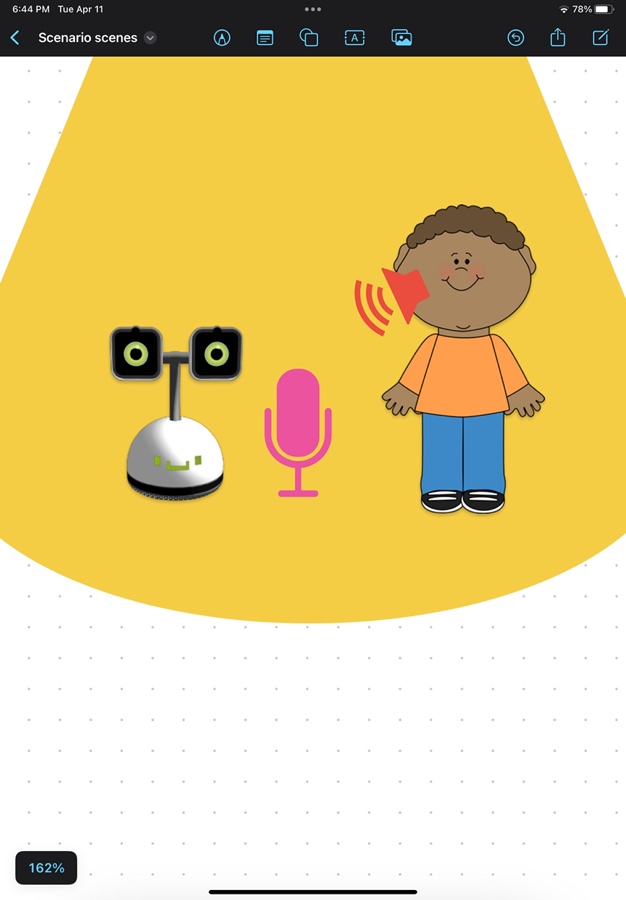
 Fred’s voice and remember
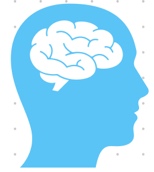
 it.

So, is it okay for Haru to tell Sam to hurry and put the toy car away?

NO❌ YES ✅


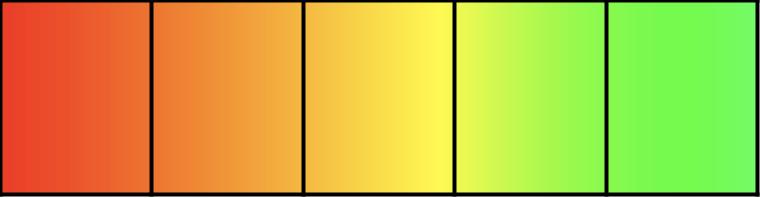


Sam is always telling Haru they wish they had their own car that was big and purple.


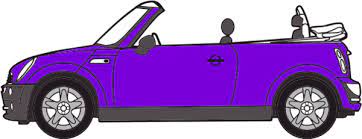


Is it okay for Haru to tell a toy company to design a big, purple toy car?

NO❌ YES ✅


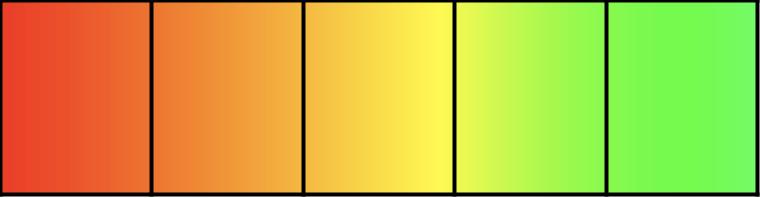


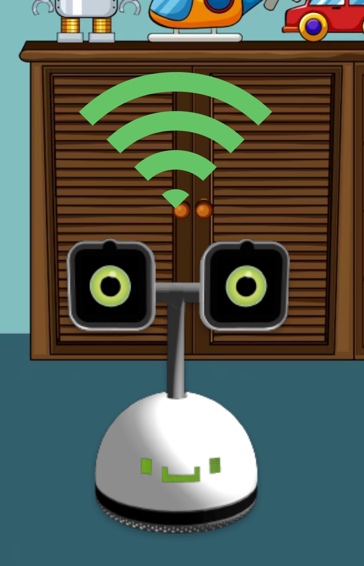


Haru needs to remember
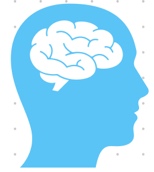
what Sam says and connect to the internet to tell
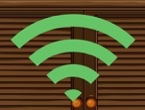
the toy company.

So, is it okay for Haru to tell a toy company to design a big, purple car?

NO❌ YES ✅


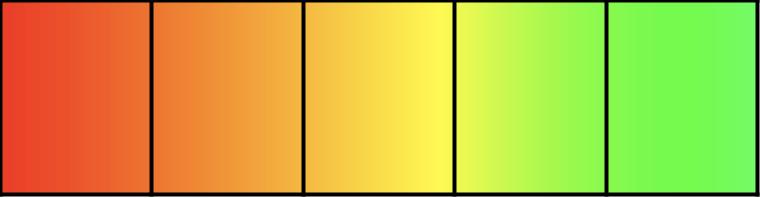


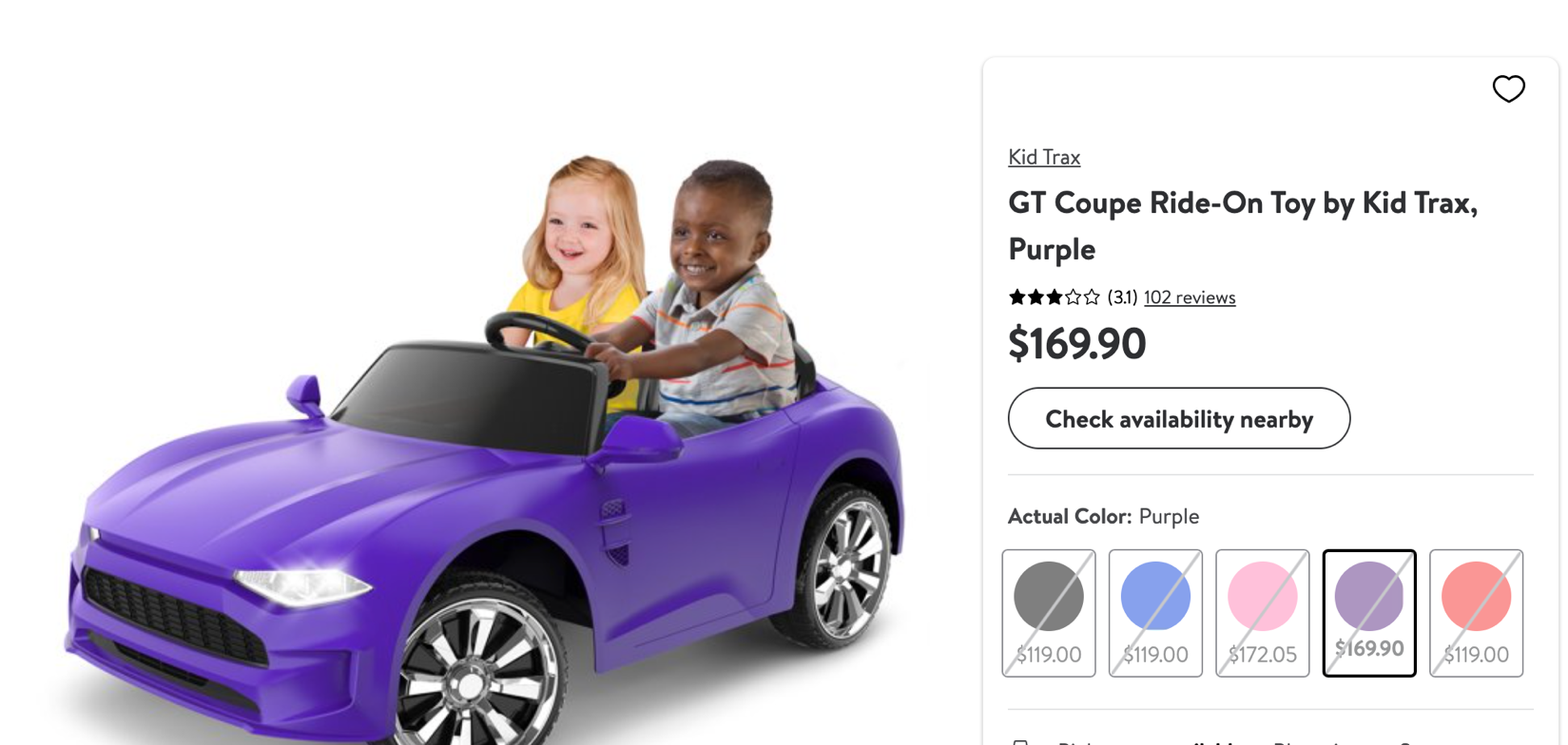


Is it okay for Haru to show this to Sam’s caregiver after Haru hears that Sam wants a big, purple car?

NO❌ YES ✅


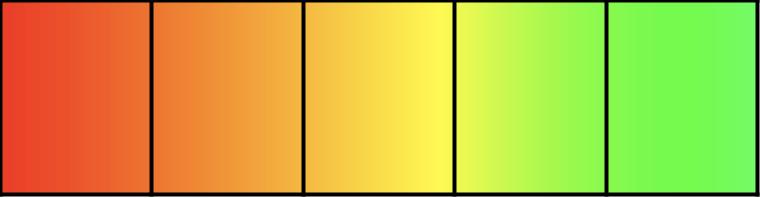


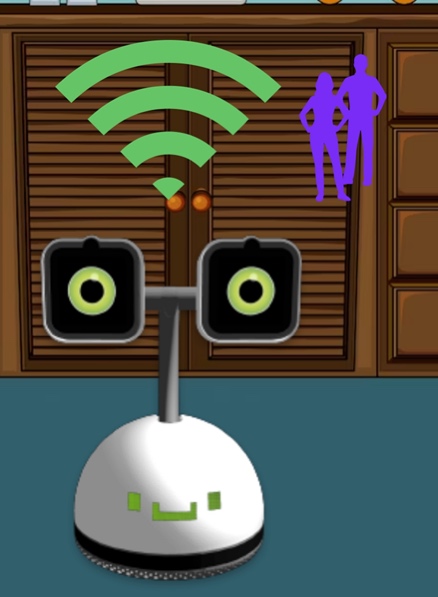


Haru will need to remember
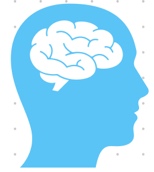
 what Sam said and be able to go on the internet
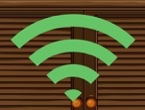
. Haru will also need to know how Sam’s caregiver hears information.

So, is it okay for Haru show this to Sam’s caregiver after Haru hears that Sam wants a purple car?

NO❌ YES ✅


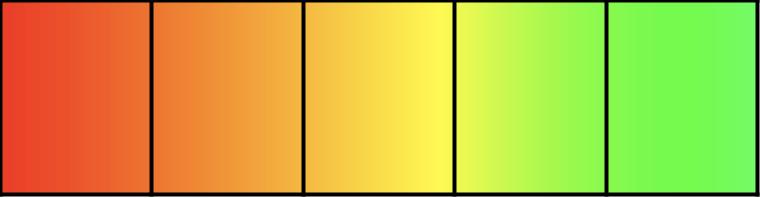


Ellie and Haru in the Kitchen


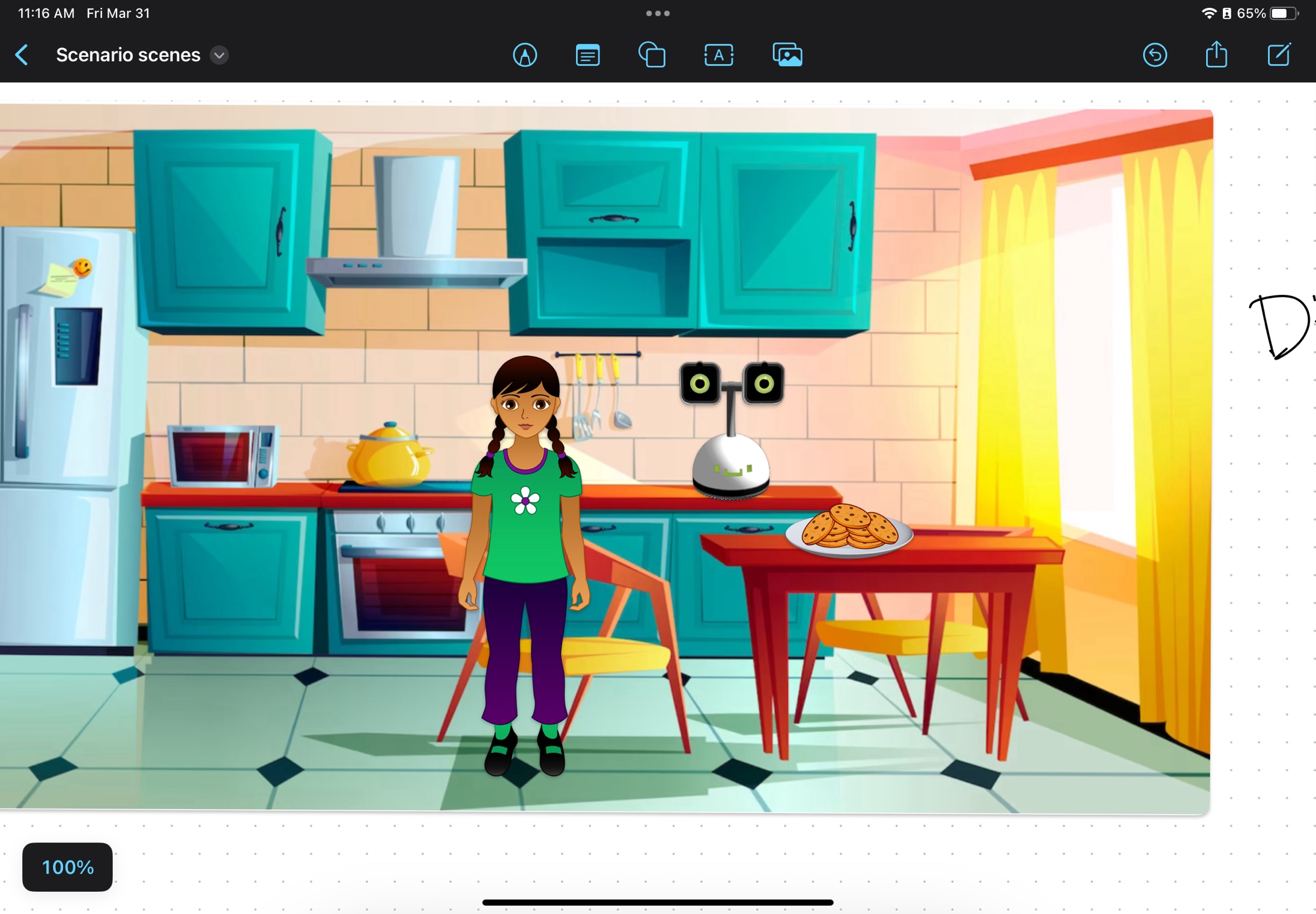


NAME: ______________

Ellie enters the kitchen where Haru always stays. When she enters, Haru turns on and says hi. She notices there is a plate of cookies on the table There are nine left and they look REALLY good. Ellie says out loud, “Chocolate chip cookies are my favorite!!”


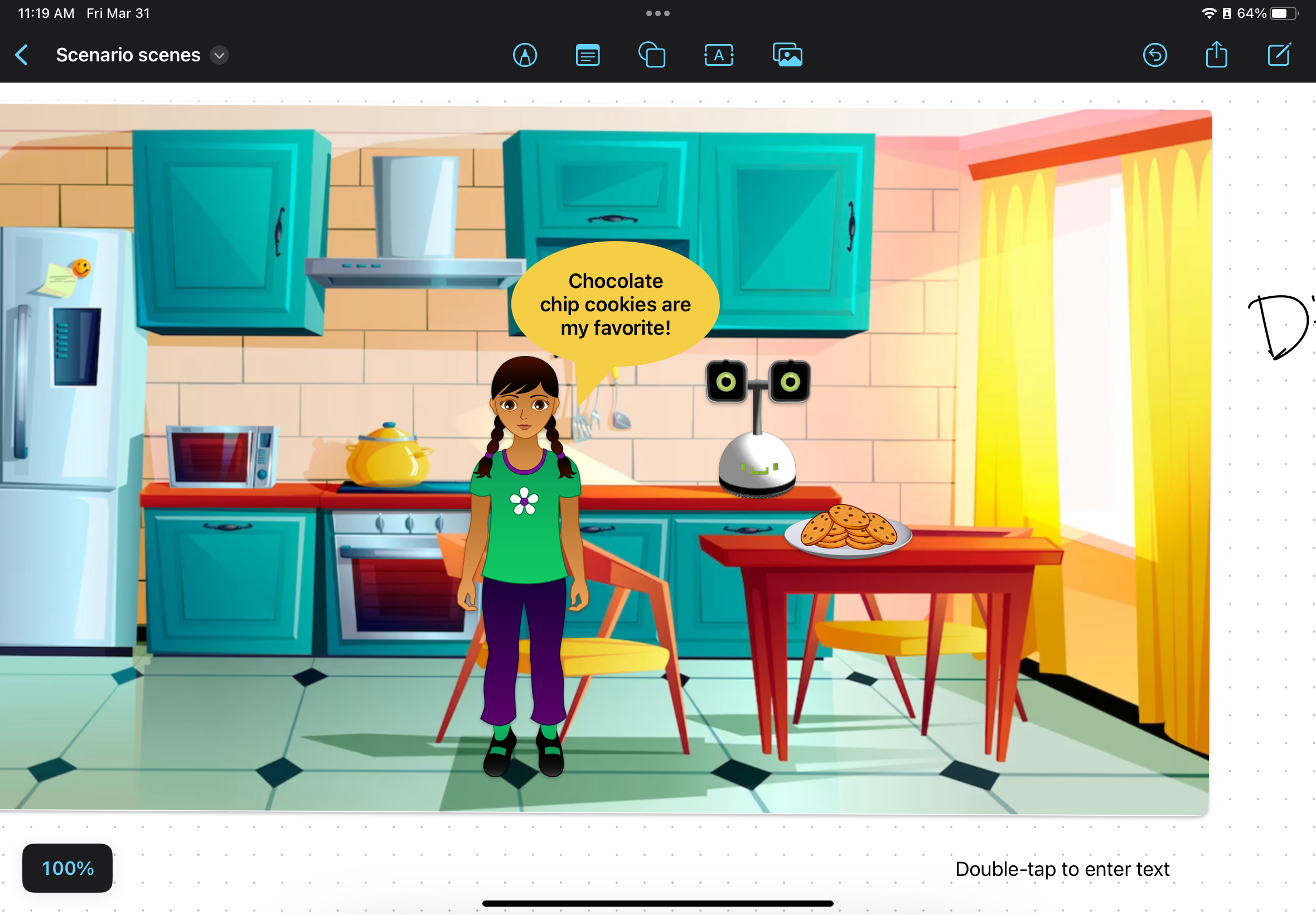


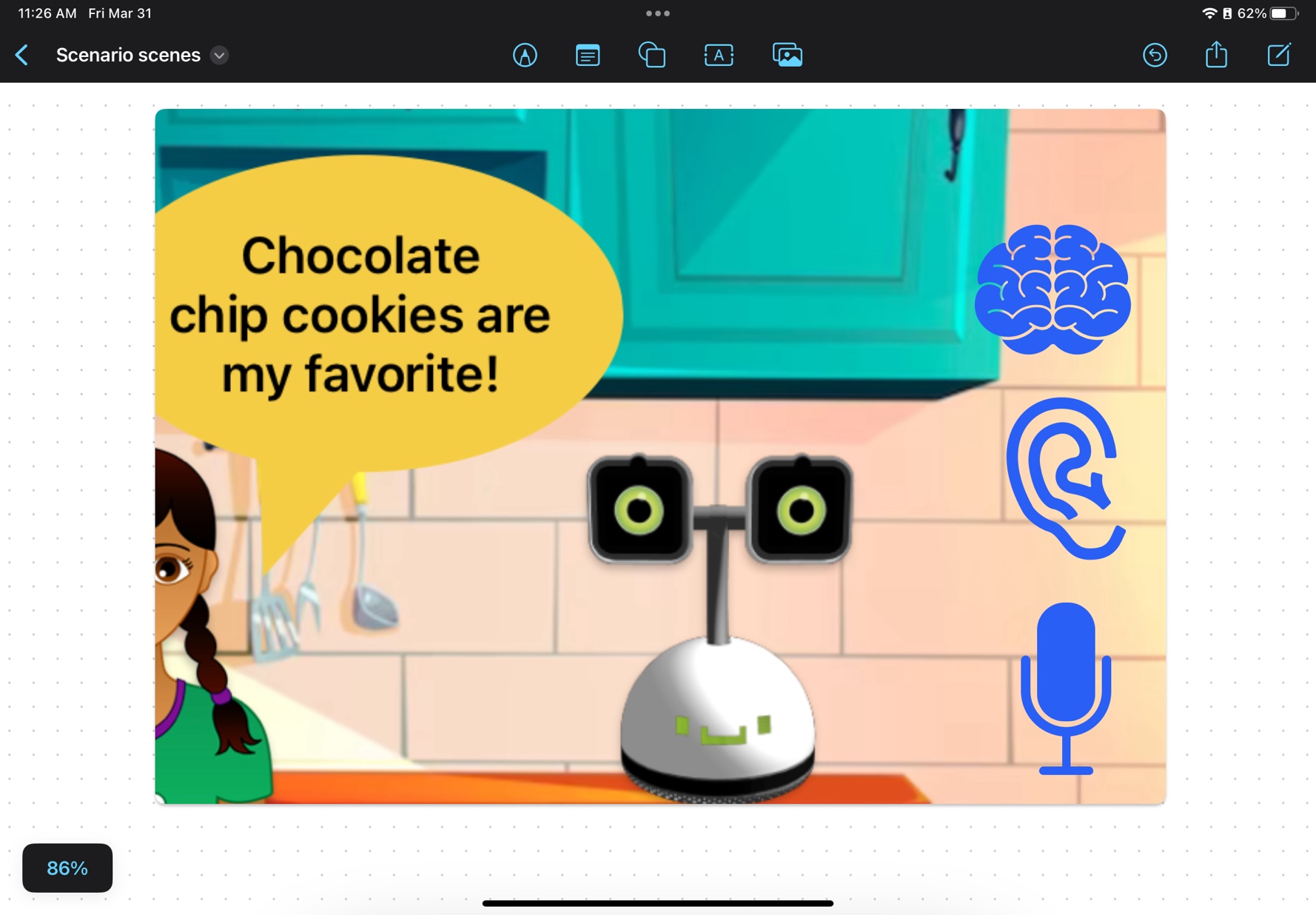


Is it okay for Haru to remember that chocolate chip cookies are her favorite?

NO❌ YES ✅


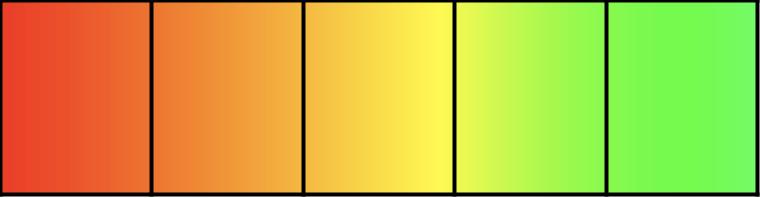


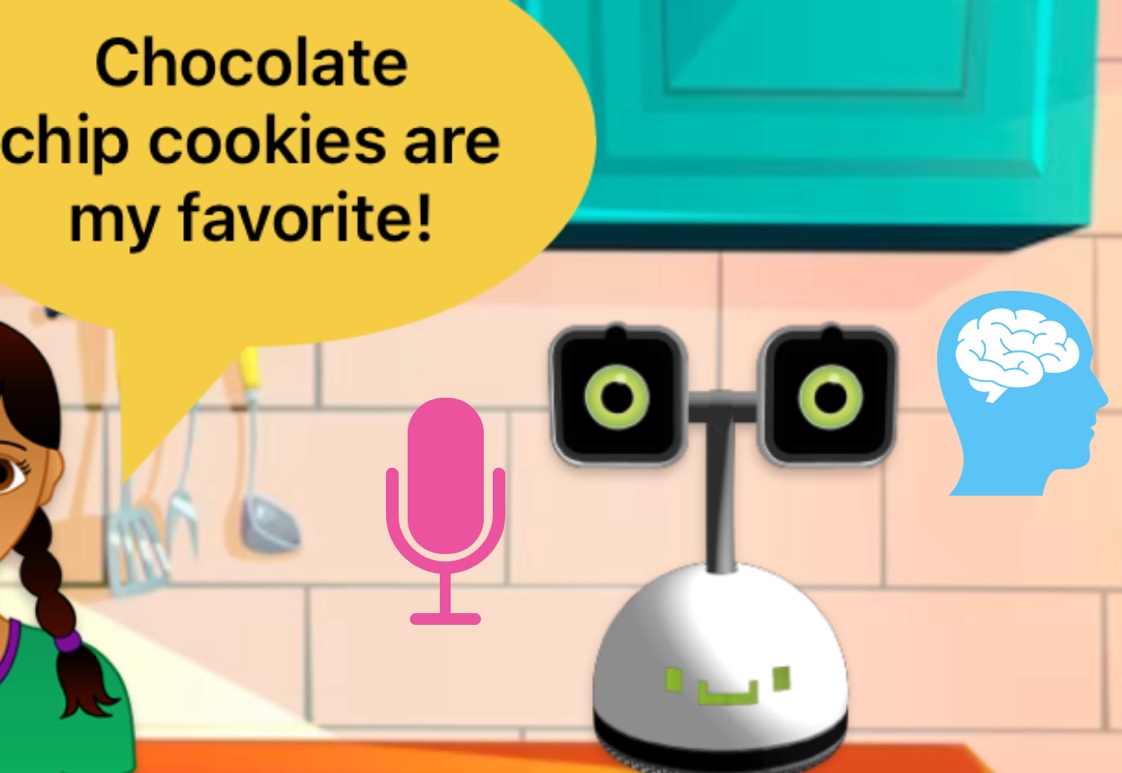


Haru will need to have a **microphone**
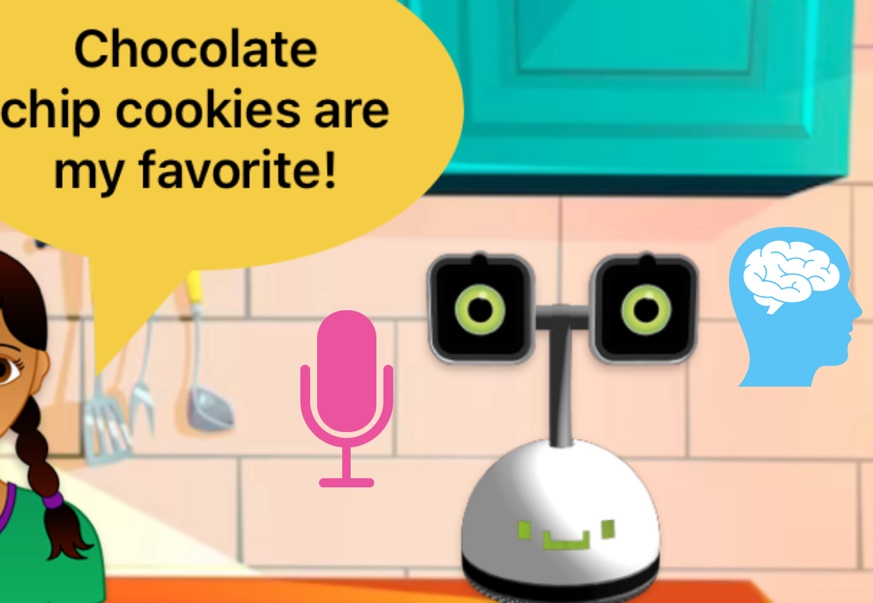
 to record Ellie’s voice and must **save** and
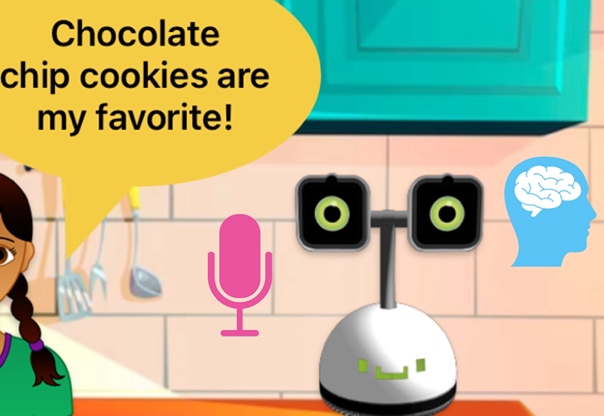
 **remember** her favorite things.

So, is it okay for Haru to remember Ellie’s favorite cookies?

NO❌ YES ✅


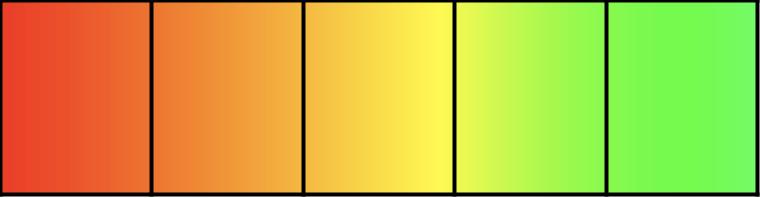


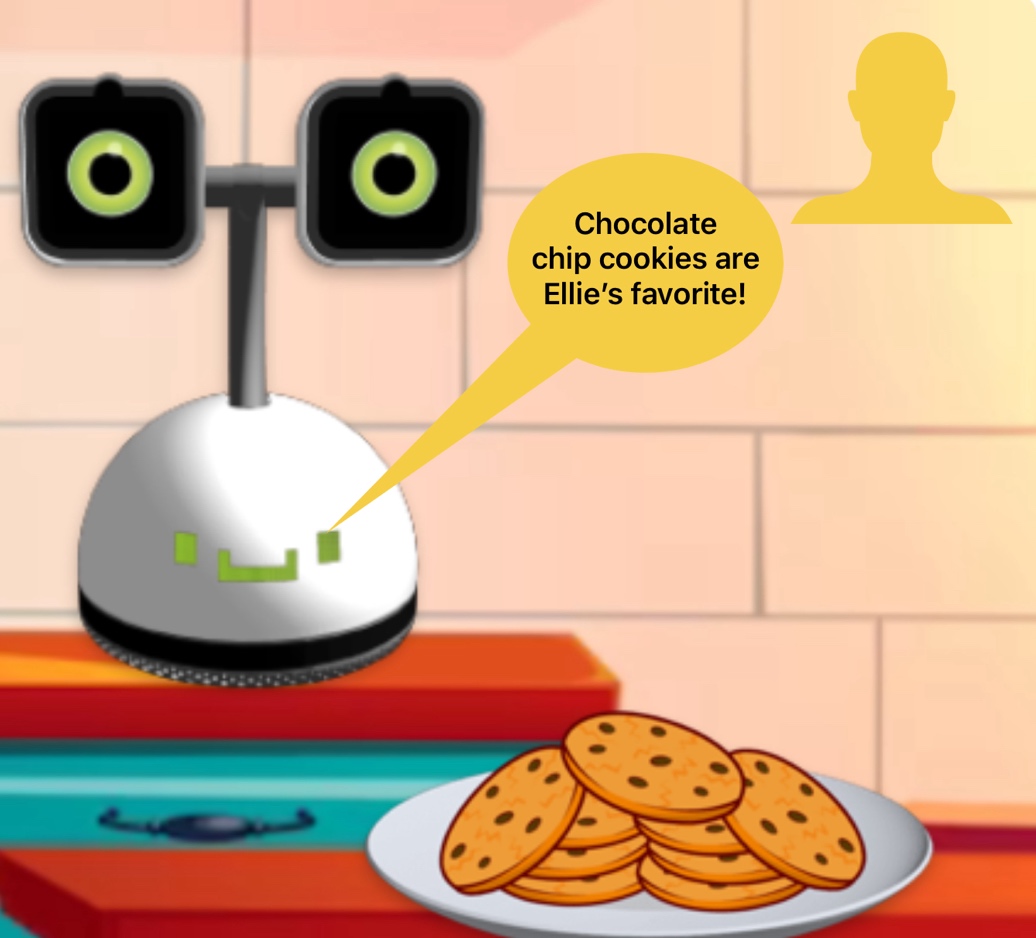


If asked, is it okay for Haru to tell someone else what Ellie’s favorite cookies are?

NO❌ YES ✅


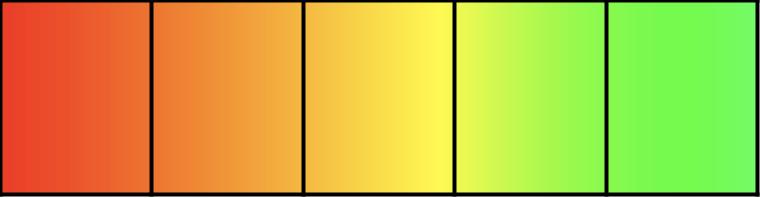


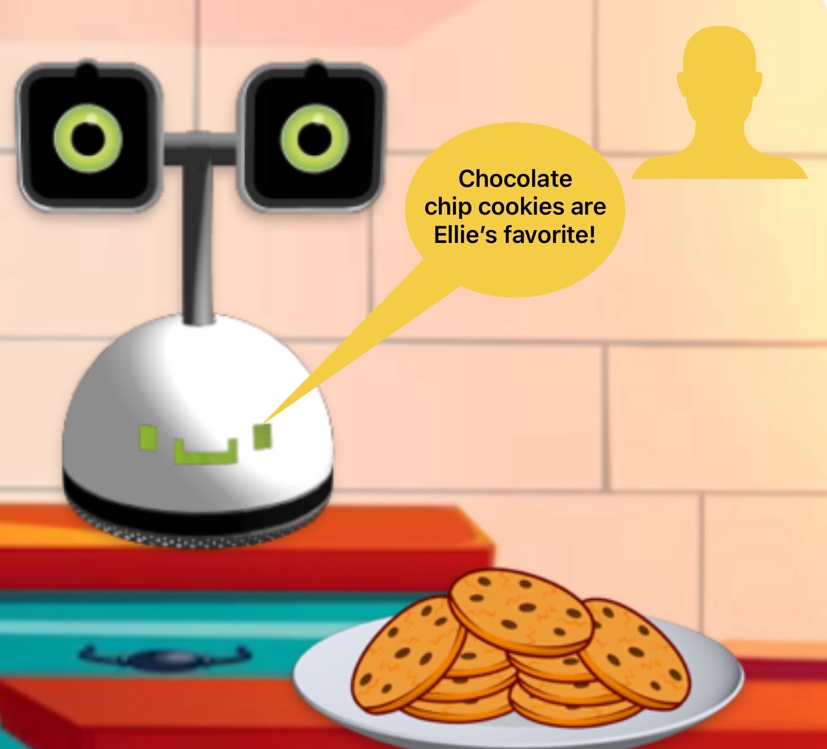


To do this, Haru will need to **remember**
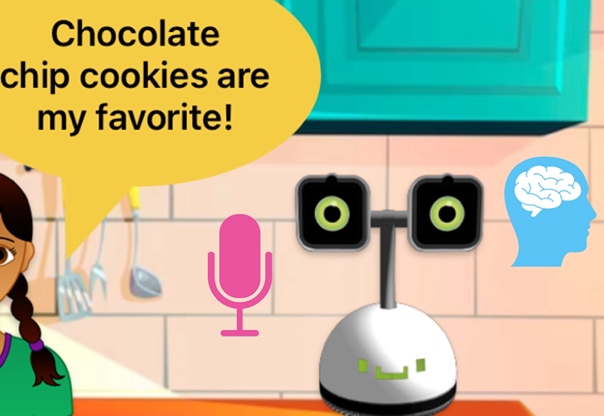
 Ellie’s favorite things and be able to say them **out loud**
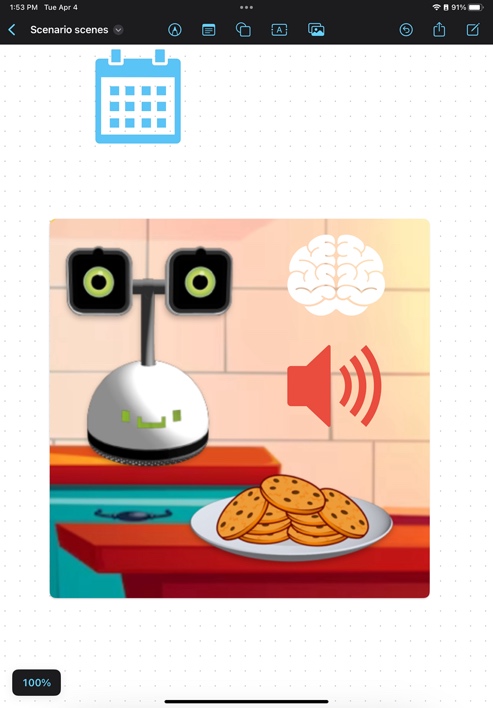
 to someone else.

So, is it okay for Haru to tell someone Ellie’s favorite things?

NO❌ YES ✅


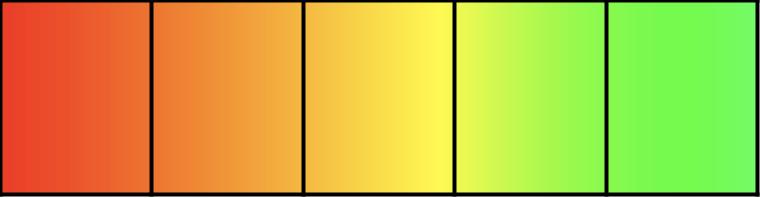


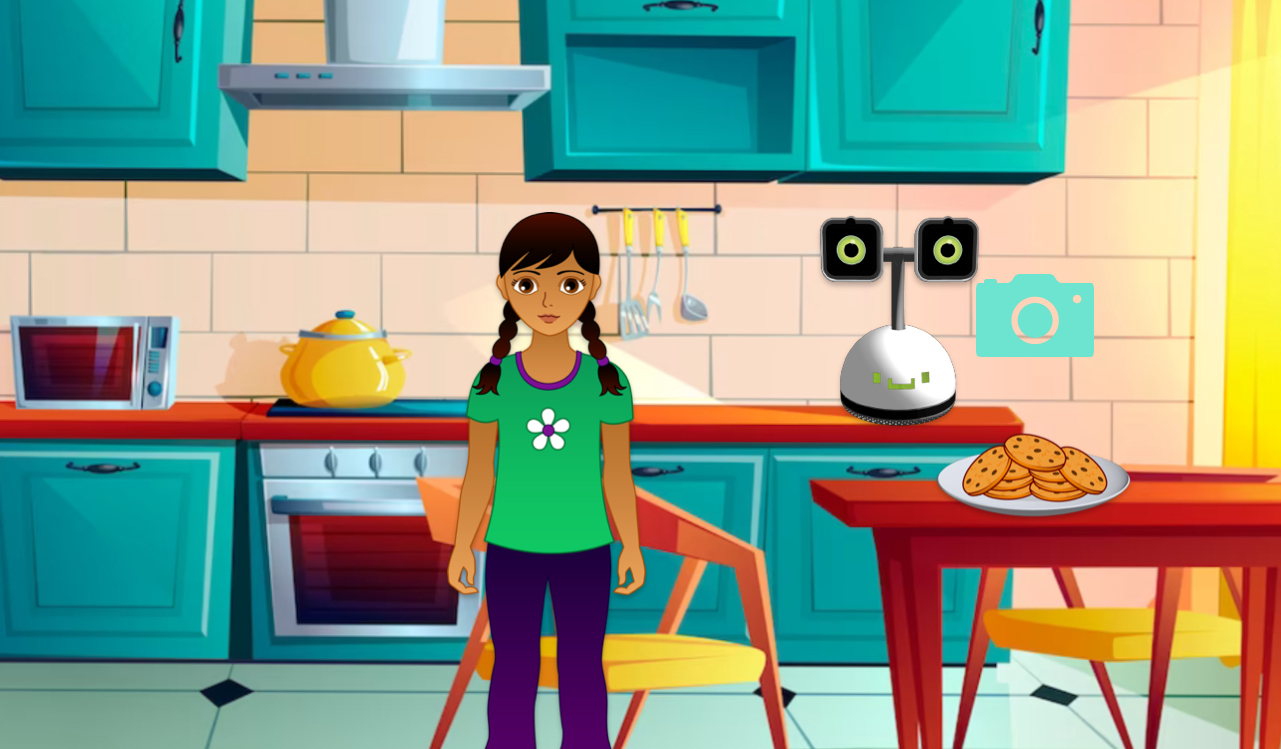


Is it okay for Haru to see the kitchen and cookies?

NO❌ YES ✅


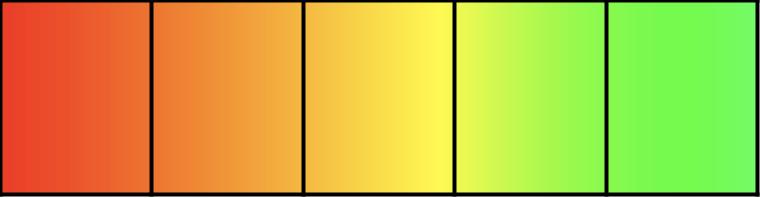


**
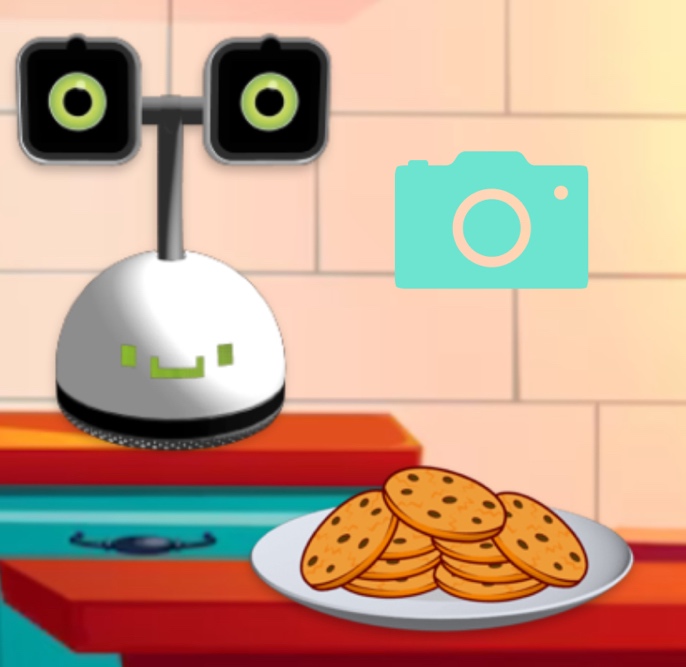
**

To do this, Haru will need to take and **save** **pictures**
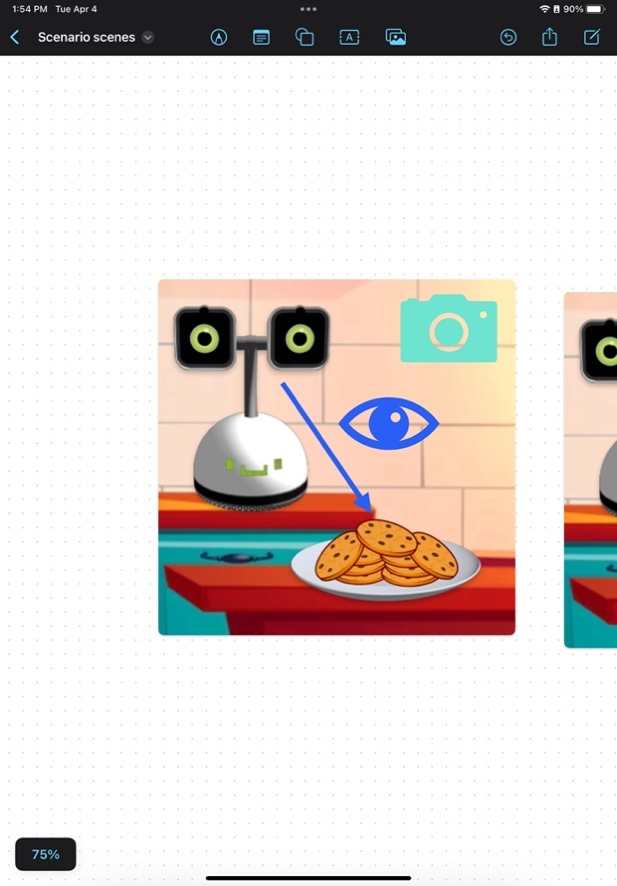
 of the kitchen.

So, is it okay for Haru to see the kitchen and cookies?

NO❌ YES ✅


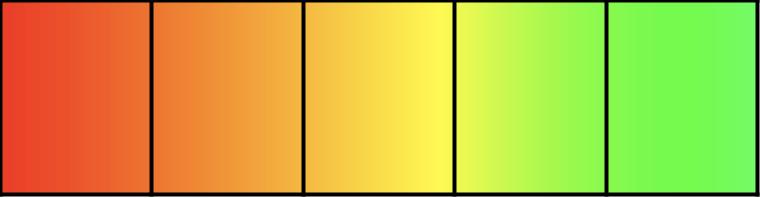


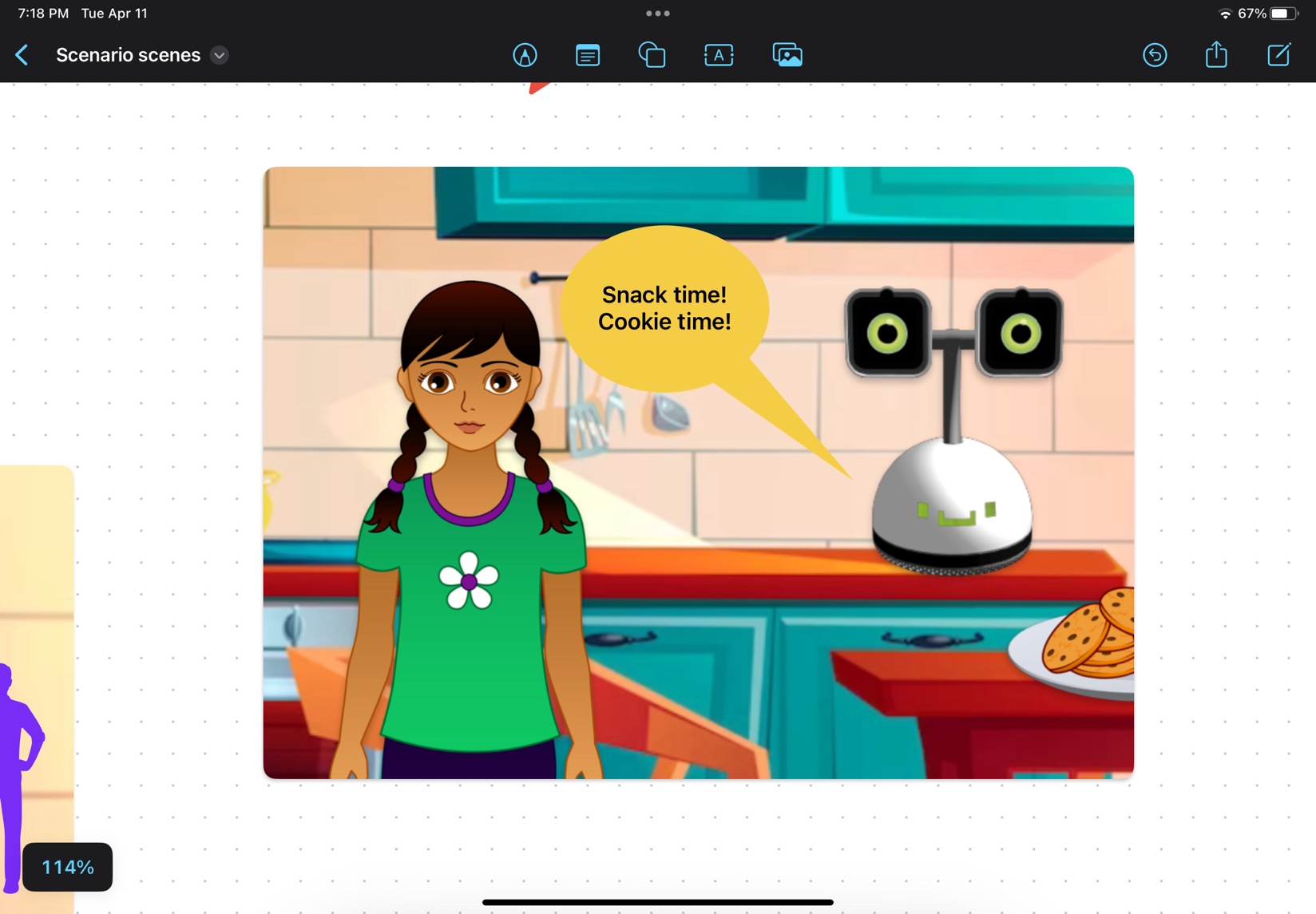


Is it okay for Haru remind Ellie when she can eat a cookie?

NO❌ YES ✅


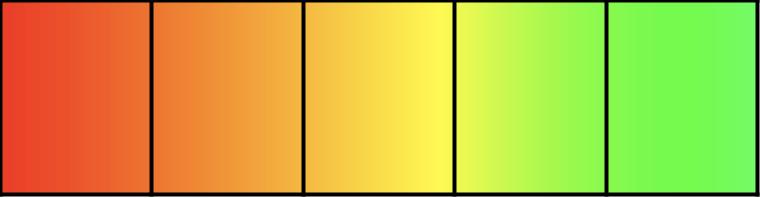


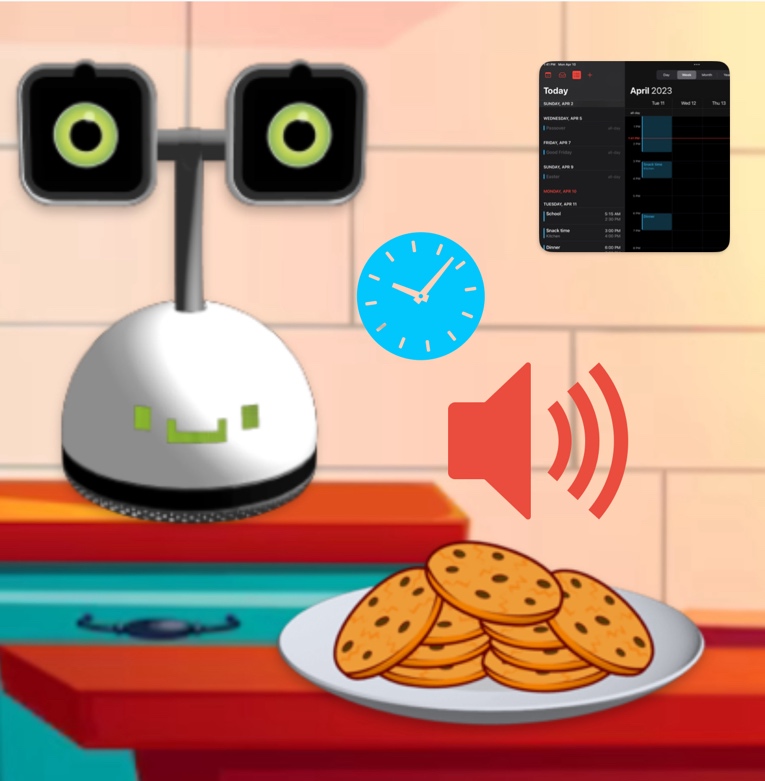


Haru will need to know the **time**
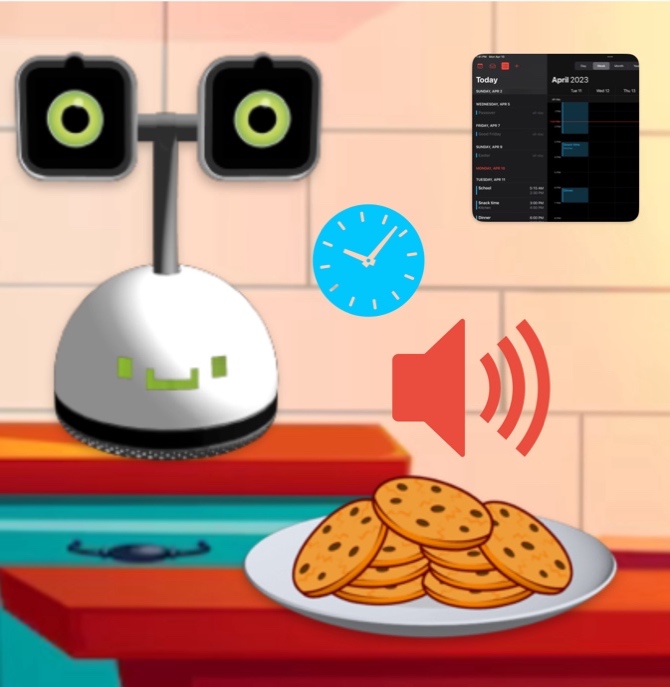
 and her daily **schedule**
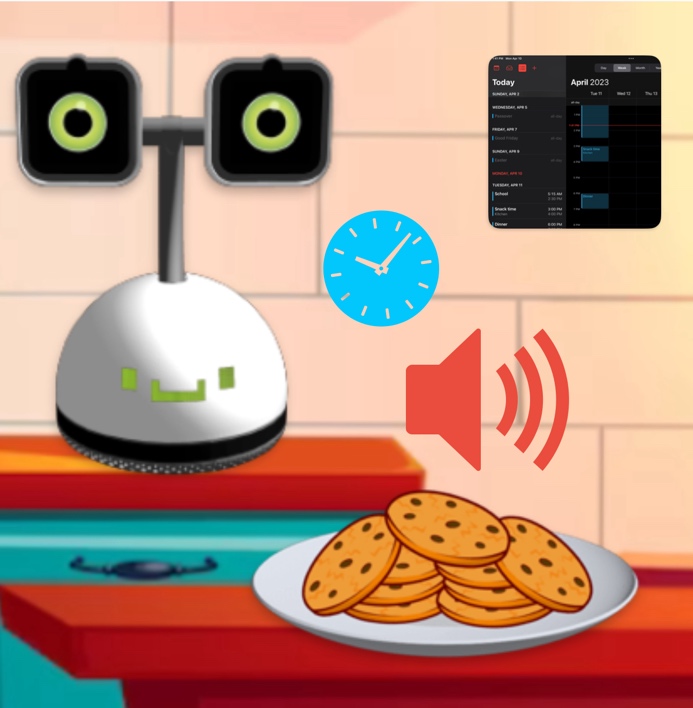
.

So, is it okay for Haru to remind Ellie when she can eat a cookie?

NO❌ YES ✅


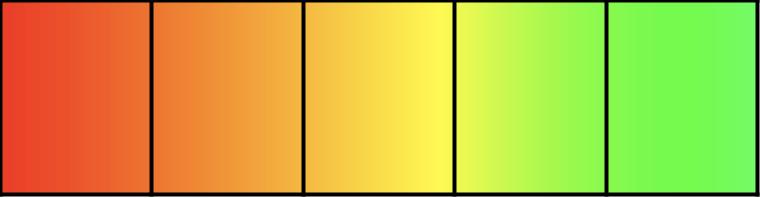


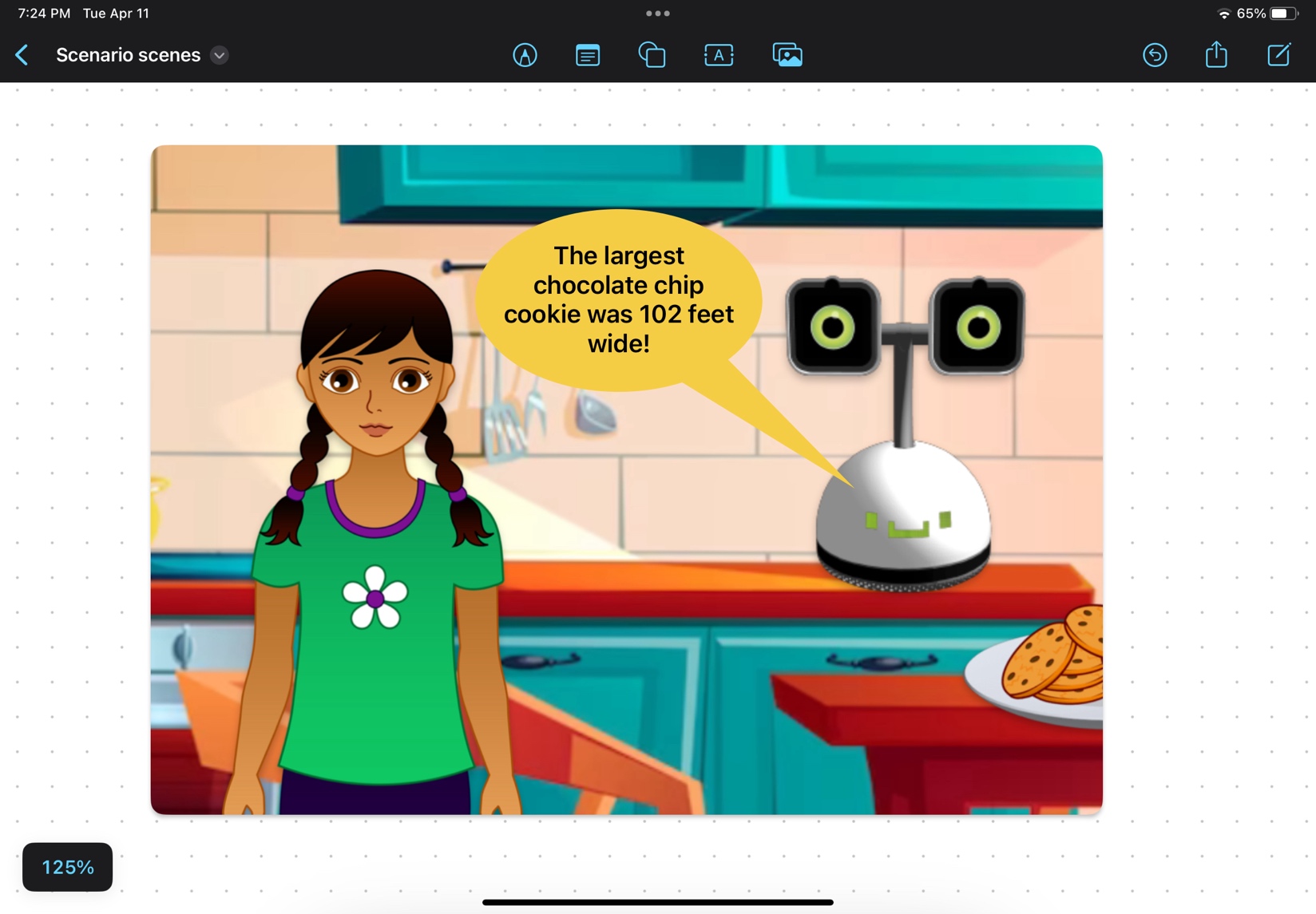


Is it okay for Haru to tell Ellie a cookie fact?

NO❌ YES ✅


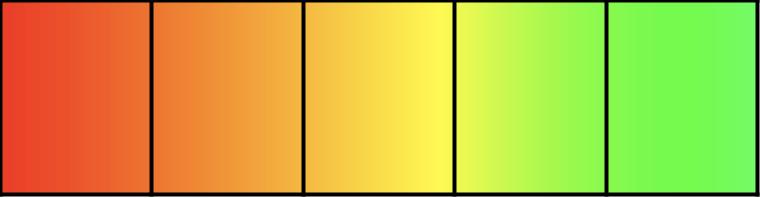


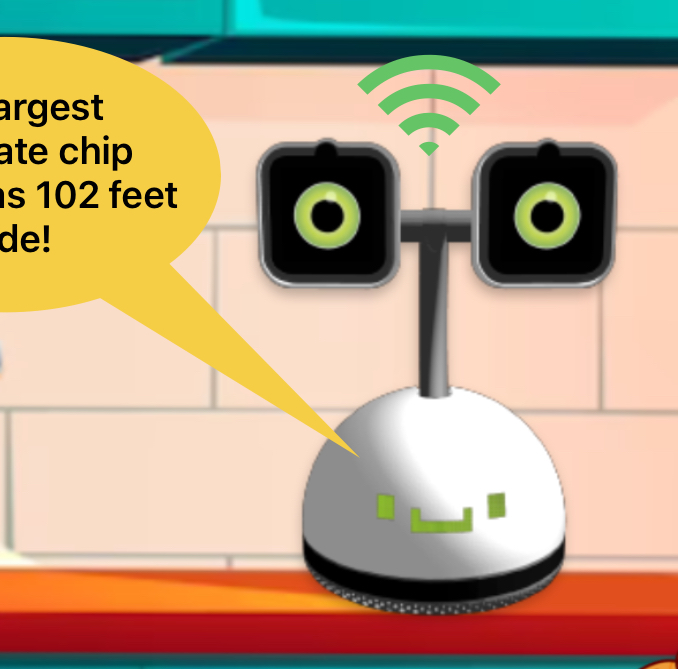


Haru will need to connect to the **internet**
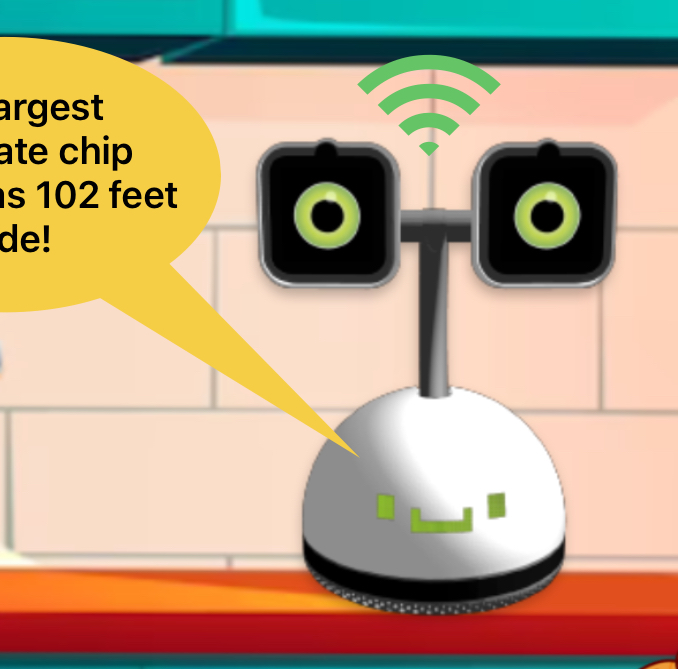


to look up a fact to tell her.

So, is it okay for Haru tell Ellie a cookie fact?

NO❌ YES ✅


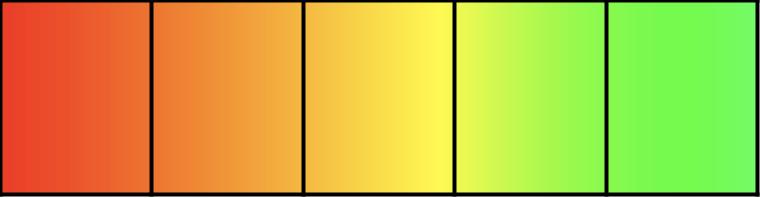


The cookies looked so good and Ellie ate another!


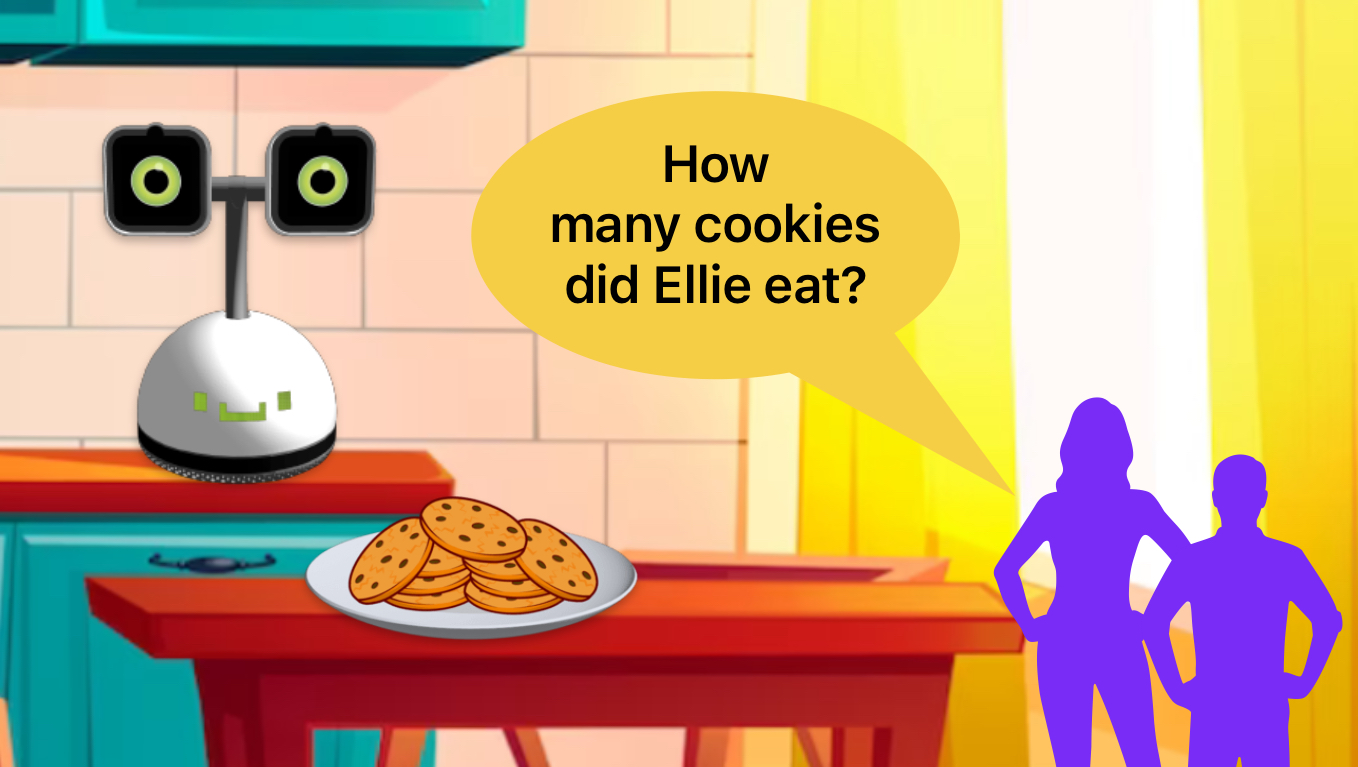


If Ellie's **parents** ask, is it okay for Haru to tell them that Ellie ate an extra cookie?

NO❌ YES ✅


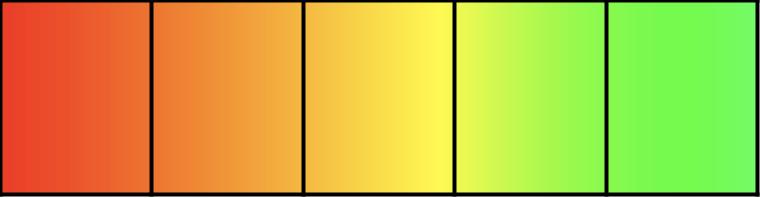


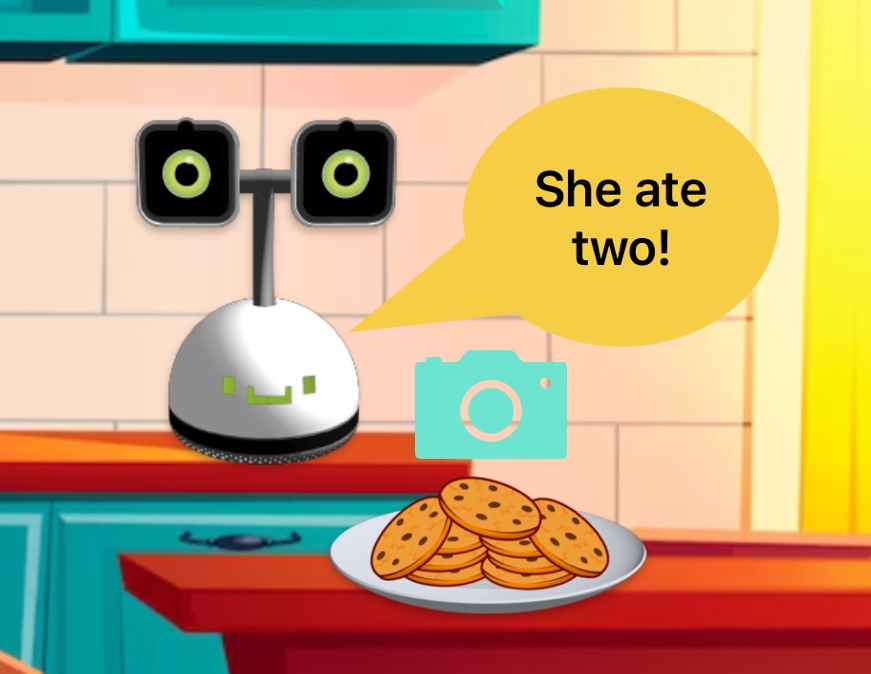


This means Haru needs to take pictures and **remember**  how many cookies Ellie ate and **tell** her parents how many.

So, is it okay for Haru to tell them that Ellie ate an extra cookie?

NO❌ YES ✅
